# Sustainable engineered cementitious composites incorporating recycled materials: Experimental validation and life cycle assessment

**DOI:** 10.1371/journal.pone.0355961

**Published:** 2026-08-28

**Authors:** Aneel Manan, Jawad Ahmad, Fawad Ahmad, Hisham Jahangir Qureshi

**Affiliations:** 1 Faculty of Civil and Environmental Engineering and Architecture, Bydgoszcz University of Science and Technology, Bydgoszcz, Poland; 2 Department of Civil and Environmental Engineering, College of Engineering, King Faisal University, Al-Ahsa, Saudi Arabia; 3 School of Civil Engineering, Iqra National University Peshawar, Peshawar, Pakistan; Graphic Era Deemed to be University, INDIA

## Abstract

The use of recycled constituents in engineering cementitious composites (ECC) offers a promising pathway to reduce the environmental burden of cement-based materials while maintaining high mechanical performance. In this study, recycled concrete powder (RCP) and waste tire steel fiber (WTSF) were incorporated into ECC, and their mechanical and environmental performance was evaluated through experimental testing, Digital Image Correlation (DIC), Scanning Electron Microscopy (SEM) and life cycle assessment (LCA). DIC was used during flexural testing to quantify surface deformation and characterize crack initiation and propagation, while SEM was employed to evaluate fiber–matrix interaction, microstructural features, and crack-bridging mechanisms. A cradle-to-gate LCA was compare the environmental impacts of cement, RCP, industrial steel fibers, WTSF, and sustainable ECC mixtures. The experimental results show that the sustainable ECC mixtures maintained competitive flexural performance, with the highest flexural strengths reaching 44.5 MPa for a mixture containing 2% WTSF and 43.5 MPa with 2% polyethylene fiber. The LCA results for the complete sustainable ECC mixtures demonstrated reductions in climate change potential and fossil resource consumption compared with conventional ECC mixtures. These findings demonstrate the potential of recycled-material-based ECC to support sustainable, low-carbon, and circular construction practices without compromising structural performance. Overall, the findings support RCP and WTSF as effective low-carbon alternatives for ECC, enabling improved sustainability without compromising structural performance.

## Introduction

Construction and demolition waste has increased significantly due to the rapid growth of urbanization and construction activities [1]. According to the reports, billions of tons of construction waste are produced, which makes a significant contribution to the global waste production [2]. Waste is primarily landfilled, with a considerable portion of the waste being concrete waste, and other materials having reactive chemical properties that cause additional environmental degradation. Conversely, fast construction boosts the need for new construction materials, such as aggregates and cement. This leads to the use of natural mountain and river reserves as construction material and is also extremely harmful to the environment. To overcome these two critical problems, researchers have studied recycled materials, and the use of RCP as coarse, fine and powder has emerged as a potential solution and alternative to both problems [3–5]. Recent studies on recycled and waste-derived cementitious composites are summarized in Table 1, highlighting the growing interest in recycled aggregate, polymer waste, and modified concrete systems for sustainable concrete. As RCP is a byproduct of crushing concrete waste into coarse and fine aggregate, due to adhered mortar, it also retains pozzolanic properties and can be used as a replacement for new cement in fresh concrete. SCMs are materials that possess pozzolanic properties and can be used as a replacement for cement, at specific proportions [6–8]. Furthermore, the researchers have reported on the improvement of mechanical and durability properties of RCP, particularly in its shrinkage control [9,10].

**Table 1:** Recent advances in recycled and sustainable cementitious composition.

Study	Cementitious system	Research focus	Key contribution and limitation
Taki et al. [11]	FA/GGBFS-based geopolymer concrete	Thermal cycling, durability, and predictive modelling	Demonstrated the performance sensitivity of sustainable binders under repeated heating–cooling cycles, but the study did not address ECC, recycled fibers, or LCA.
Su et al. [23]	Concrete with recycled tire steel fibers	Mechanical and durability performance of recycled tire steel fiber concrete	Supports the use of recycled tire steel fibers, but the system is conventional concrete rather than an integrated sustainable ECC assessment.
Sabetifar et al. [12]	PET/rubber-modified concrete with CFRP confinement	Residual axial performance after thermal exposure	Showed that recycled PET and rubber influence strength, stiffness, ductility, and toughness, but the system was based on conventional concrete rather than ECC.
Sasania et al. [13]	High-strength concrete with recycled nylon granules	Thermo-mechanical response and predictive modelling	Confirmed that polymer waste can support waste valorization, although excessive replacement may reduce mechanical performance.
Nematzadeh and Hasan-Ghasemi [14]	PET-based self-consolidating concrete	Size effect, damage behavior, and high-temperature performance	Highlighted the need for detailed mechanical validation of recycled-material concretes, especially when damage mechanisms are affected by specimen size and exposure conditions.
Valizadeh Kiamahalleh et al. [15]	PVA-treated recycled concrete aggregate concrete	Improvement of RCA properties and concrete performance	Demonstrated that recycled concrete-derived materials can be improved through pretreatment, but dosage optimization remains necessary to balance workability, strength, and durability.

ECC has been recognized for its high tensile capacity, strain hardening responses, and ability to form multiple fine cracks under loading [16–18]. However, these benefits are commonly achieved through high cement and fiber contents, which can increase the environmental burden of ECC production. However, SCMs like fly ash, ground granulated blast furnace slag (GGBS), silica fume and RCP have shown a potential to be used in ECC as cement replacements. Among these materials, RCP offers additional benefits by reducing cement consumption, utilizing construction waste, and limiting the demand for newly extracted raw materials [19–21]. The environmental performance of ECC is also influenced by the type of fiber reinforcement. Industrial steel fiber requires energy intensive production, whereas waste tire steel fiber offers a potential recycled alternative that can reduce tire waste while maintaining crack resistance, flexural capacity, and energy absorption [22,23]. More recently, the use of supplementary cementitious materials and recycled constituents has also been shown to have a substantial contribution towards sustainability in ECC without compromising the required mechanical properties. It has been reported that recycled concrete powder can improve the matrix densification, filler effect and enhancement of the hydration process of the cementitious composites, which are beneficial to the crack resistance and strength development. Likewise, for flexural loading, ECC made with hybrid fibres has exhibited better strain-hardening characteristics, energy absorption and crack-bridging ability [24].

Moreover, the ability of DIC techniques to provide full-field deformation measurements and detailed characterization of damage has attracted attention for the evaluation of the strain localization and crack propagation in ECC systems. WTSF also has environmental advantages because it is a cost-effective material when compared to industrial steel fibre, which emits carbon emissions into the environment. Previous studies by the authors have addressed the sustainability and structural behavior of recycled concrete aggregate (RCA) systems [25], including a comprehensive life cycle assessment of RCA [26] and the evaluation of RCA concrete structural members such as columns [27]. Nevertheless, the use of waste derived constituents cannot automatically be considered environmentally beneficial because their collection, processing, transportation, and manufacturing may create impacts in categories other than carbon emissions [28]. LCA is therefore necessary to determine whether relacing cement with RCP and industrial steel fiber with WTSF provides genuine environmental benefits without transferring burdens to fossil resource use, toxicity, particulate emissions, acidification, eutrophication, land occupation or water consumption.

Therefore, this study is conducted to examine the crack propagation and crack pattern to gain deeper knowledge of the mechanisms inside the sustainable ECC material with the inclusion of RCP and WTSF. Specifically, crack behavior is investigated in conjunction with SEM based microstructural characterization, to gain an understanding of the effect of RCP on matrix densification, fiber–matrix interaction, crack-bridging behaviour and damage evolution in sustainable ECC systems. A detailed LCA is also conducted to quantify the environmental impacts resulting from material substitutions and reinforcement options. The LCA scope includes 18 impact categories and takes into account the impacts at the manufacturing stage of the constituents used in the cement: RCP, industrial steel fibre and waste steel fibre. This will allow for comparative analysis of the conventional and waste-derived constituents and provide a framework for making mix design decisions in a way that is more sustainable. An environmental assessment was performed for the ECC (cradle to gate) with a functional unit of 1 m^3^ ECC produced. The LCA framework was created by importing LCI data for the conventional and recycled ECC components from the life cycle inventory database of the ecoinvent platform, included in the openLCA framework and presents the environmental impacts of the manufacturing stage. Hence, this research will focus on gaining a complete knowledge of the mechanical properties, crack propagation behavior, microstructure, and sustainability of ECC using recycled materials to develop sustainable and low-carbon cementitious composites.

### Research significance

The significant of this study lies in establishing an integrated framework that connects structural performance, damage mechanism, and environmental sustainability in ECC incorporating RCP and WTSF. Instead of considering these recycled constituents only as replacements for cement and industrial fibers, the study examines how they influence matrix quality, fiber and matrix interaction, strain localization, crack initiation, crack propagation, flexural resistance, and post cracking ductility. Flexural testing is combined with DIC and SEM observations to determine not only whether the sustainable mixtures achieve competitive performance, but also the mechanisms responsible for their behavior.

The environmental contribution extends beyond a simple comparison of carbon emissions. A cradle to gate LCA, based on a functional unit of 1 m^3^ of ECC, evaluates raw material extraction, processing, transportation, recycling, and manufacturing using the ReCiPe 2016 midpoint method. Environmental impacts are examined at three interconnected level: cement compared with RCP, industrial steel fiber compared with WTSF, and conventional ECC compared with complete sustainable ECC mixtures. The assessment includes climate change, fossil resource depletion, toxicity, particulate matter formation, acidification, eutrophication, and water consumption. By linking mechanical efficiency, crack control, microstructural behavior, waste utilization, and multiple environmental indicators, the study provides a comprehensive basis for identifying ECC mixtures that achieve an effective balance between structural performance, carbon reduction, and circular resource use.

## Research methodology

**Fig 1:**
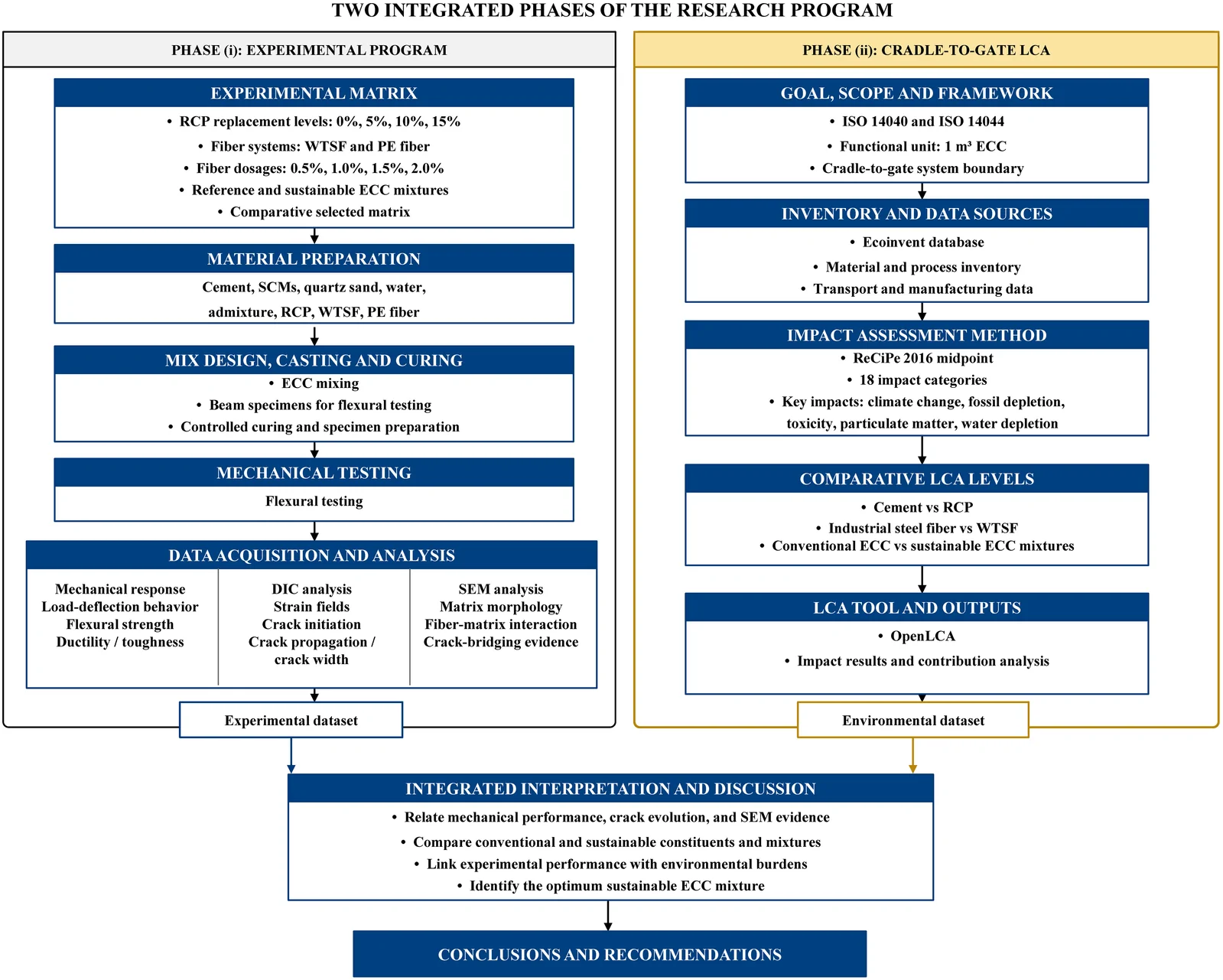
Overview of the two-phase research methodology.

The experimental programme aimed at testing the mechanical properties, crack propagation characteristics and environmental sustainability of ECC with the addition of RCP and WTSF. The methodology was divided into two integrated phases, as shown in Fig 1: (i) experimental characterization, which included preparation of materials, casting of specimens, curing, flexural testing, monitoring by DIC, and analysis by SEM; and (ii) cradle to gate LCA for evaluation of the environmental impacts of the proposed sustainable ECC mixtures. Adopted as partial replacement of cement at 5% level, 10% level, 15% level; and as a replacement of industrial steel fiber at 0.5%, 1.0%, 1.5% and 2.0% level respectively. Furthermore, to strengthen the bridging effect, the same proportion of PE fiber as in the WTSF mix is added to the mix. In the testing procedure, the surface deformation and crack patterns will be monitored and analyzed during loading, using DIC.

At last, the internal morphology is investigated using SEM. The sustainable ECC mixtures were particularly studied by SEM to assess the matrix densification characteristics, fiber–matrix bonding characteristics, microcrack development and interfacial transition behavior. The second phase is an LCA study to gain an understanding of the environmental impacts of a detailed use of recycled materials versus new materials, with a list of 18 impact categories. In this LCA study, the software program openLCA is applied, and the procedures are taken from the Ecoinvent database, which contain up-to-date and detailed procedures for a cradle-to-gate analysis. The Life Cycle Assessment framework is based on ISO 14040 and ISO 14044 standards. The evaluated key impact categories are climate change, fossil fuel depletion, water depletion and human toxicity.

### Materials

The material used to develop sustainable ECC includes cement, RCP (partial cement replacement), silica fume (SF), sand, water, WTSF and polyethene (PE) fibers. OPC served as the primary binder, while slag and silica fume were incorporated as supplementary cementitious materials to improve matrix densification, durability, and sustainability. Quartz sand was used as the fine aggregate to enhance particle packing and support the formation of a dense ECC matrix. RCP was used as a partial cement replacement to reduce cement consumption and the associated CO_2_ emissions from cement production. WTSF and PE fibers were added to enhance ductility, crack resistance, and flexural performance.

Different WTSF and PE fibre contents were evaluated to assess their individual and combined effects in sustainable RCP-based ECC. Both fibers were incorporated at dosage levels corresponding to 0.5%, 1%, 1.5%, and 2.0% by mass of binder for comparative experimental evaluation. It should be noted that due to the significant density difference between WTSF and PE fibers, the corresponding fiber volume fractions differ, which may influence fiber spacing, crack-bridging behavior, and strain distribution within the ECC matrix. A systematic programme was adopted to evaluate tensile strength, ductility, and crack control. WTSF was selected for its sustainability benefits and mechanical contribution, while PE fiber was included for its crack-bridging capacity and tensile strain performance. The fiber properties are summarized in Table 2.

**Table 2:** Physical and mechanical properties of fibers [26].

Fiber Type	Diameter (μm)	Length (mm)	Density (g/cm^3^)	Tensile Strength (MPa)	Modulus of Elasticity (GPa)
PE Fiber	25	12	0.95	≥3500	125
WTSF	190	13	7.85	≥2900	215

It is recognized that increasing fiber content, particularly WTSF incorporation, may influence the fresh-state workability and flow characteristics of ECC mixtures due to fiber interlocking and increased internal friction.

### Mix design and experimental setup

The making process of sustainable ECC samples were systemically design as ECC requires extra care and attention in the preparation process. The process started from dry mixing of material which includes cement slag, SF and RCP, which were mixed for 2–3 minutes to make it uniform. While in the water a high-range water-reducing admixture was added and mixed for 10–12 minutes for a consistent paste. WTSF and PE fibers were incrementally incorporated into the mixture and blended for 5–6 minutes to achieve consistent dispersion. Subsequently, the newly developed sustainable ECC mixtures were poured into molds, and the workability of each mixture was assessed using a mini-slump apparatus according to ASTM C1437 guidelines [29]. The sample was cast and after 24 hours it was demolded and set for accelerated curing in the water of 85^o^C for 9 days to active the pozzolanic property within the SCMs. The accelerated curing is very useful when using SCMs because the hydration process is optimized and after the curing the samples were stored at the room temperature of (20-24^o^C).

The mix design for sustainable ECC with different materials and their proportions is presented in Table 3. RCP, WTSF and PE fibers are the main variable constituents in this study, while slag, silica fume, quartz sand, water, and superplasticizer were kept constant unless otherwise stated. key materials with different percentages, which are highlighted in the nomenclature of the samples. For instance, RCP-10-ECC-WTSF (0.5-2) indicates that RCP refers to recycled concrete powder, and 10 denotes the amount of RCP in percentage replacement level with cement in sustainable ECC. As indicated in the sample set, there are four types of mixes with 0%, 5%, 10% and 15% RCP. Two fiber types were used, namely WTSF and PE fiber. However, when the WTSF percentage was varied, the PE fiber content was kept constant, and the same approach was applied when PE fiber was varied. The fiber content was varying at 0.5%, 1.0%, 1.5% and 2.0%, while the other fiber was maintained at a constant 2.0% to isolate the effect of fiber variation on the performance of sustainable-ECC. The water-to-binder ratio was 0.318, and the superplasticizer dosage was kept constant at 2.25 kg/m^3^, corresponding to 0.15% by cement mass.

**Table 3:** Mix design of sustainable ECC [26].

Sample	Cement	RCP	Slag	Silica Fume	Quartz sand	Water	Super- plasticizer	PE fiber	WTSF fiber
UHP-ECC-Ref (ratio)	1	0	0.125	0.25	0.375	0.318	0.0015	0.02	0
UHP-ECC-Ref (kg/m^3^)	1500	0	187.5	375	562.5	477	2.25	30	0
RCP-5-ECC-SF0.5	1425	75	187.5	375	562.5	477	2.25	30	7.5
RCP-5-ECC- WTSF 1	1425	75	187.5	375	562.5	477	2.25	30	15
RCP-5-ECC- WTSF 1.5	1425	75	187.5	375	562.5	477	2.25	30	22.5
RCP-5-ECC- WTSF 2	1425	75	187.5	375	562.5	477	2.25	30	30
RCP-10-ECC- WTSF 0.5	1350	150	187.5	375	562.5	477	2.25	30	7.5
RCP-10-ECC- WTSF 1	1350	150	187.5	375	562.5	477	2.25	30	15
RCP-10-ECC- WTSF 1.5	1350	150	187.5	375	562.5	477	2.25	30	22.5
RCP-10-ECC- WTSF 2	1350	150	187.5	375	562.5	477	2.25	30	30
RCP-15-ECC- WTSF 0.5	1275	225	187.5	375	562.5	477	2.25	30	7.5
RCP-15-ECC- WTSF 1	1275	225	187.5	375	562.5	477	2.25	30	15
RCP-15-ECC- WTSF 1.5	1275	225	187.5	375	562.5	477	2.25	30	22.5
RCP-15-ECC- WTSF 2	1275	225	187.5	375	562.5	477	2.25	30	30
RCP-5-ECC-PE0.5	1425	75	187.5	375	562.5	477	2.25	7.5	30
RCP-5-ECC-PE1	1425	75	187.5	375	562.5	477	2.25	15	30
RCP-5-ECC-PE1.5	1425	75	187.5	375	562.5	477	2.25	22.5	30
RCP-5-ECC-PE2	1425	75	187.5	375	562.5	477	2.25	30	30
RCP-10-ECC-PE0.5	1350	150	187.5	375	562.5	477	2.25	7.5	30
RCP-10-ECC-PE1	1350	150	187.5	375	562.5	477	2.25	15	30
RCP-10-ECC-PE1.5	1350	150	187.5	375	562.5	477	2.25	22.5	30
RCP-10-ECC-PE2	1350	150	187.5	375	562.5	477	2.25	30	30
RCP-15-ECC-PE0.5	1275	225	187.5	375	562.5	477	2.25	7.5	30
RCP-15-ECC-PE1	1275	225	187.5	375	562.5	477	2.25	15	30
RCP-15-ECC-PE1.5	1275	225	187.5	375	562.5	477	2.25	22.5	30
RCP-15-ECC-PE2	1275	225	187.5	375	562.5	477	2.25	30	30

### Experimental setup and procedure

The flexural performance of sustainable ECC was evaluated using beam specimens under three-point bending conditions in accordance with ASTM C348. A total of 75 beam specimens were prepared and tested, corresponding to 25 mixture groups with three replicate specimens for each mixture. Each beam specimen was of a rectangular size with a cross-section of 40 mm x 40 mm and 160 mm in length. Before testing a speckle, a pattern was used to cover the testing side of the sample for DIC, which was used during the test to determine and understand a crack pattern, mainly in the central region.

**Fig 2:**
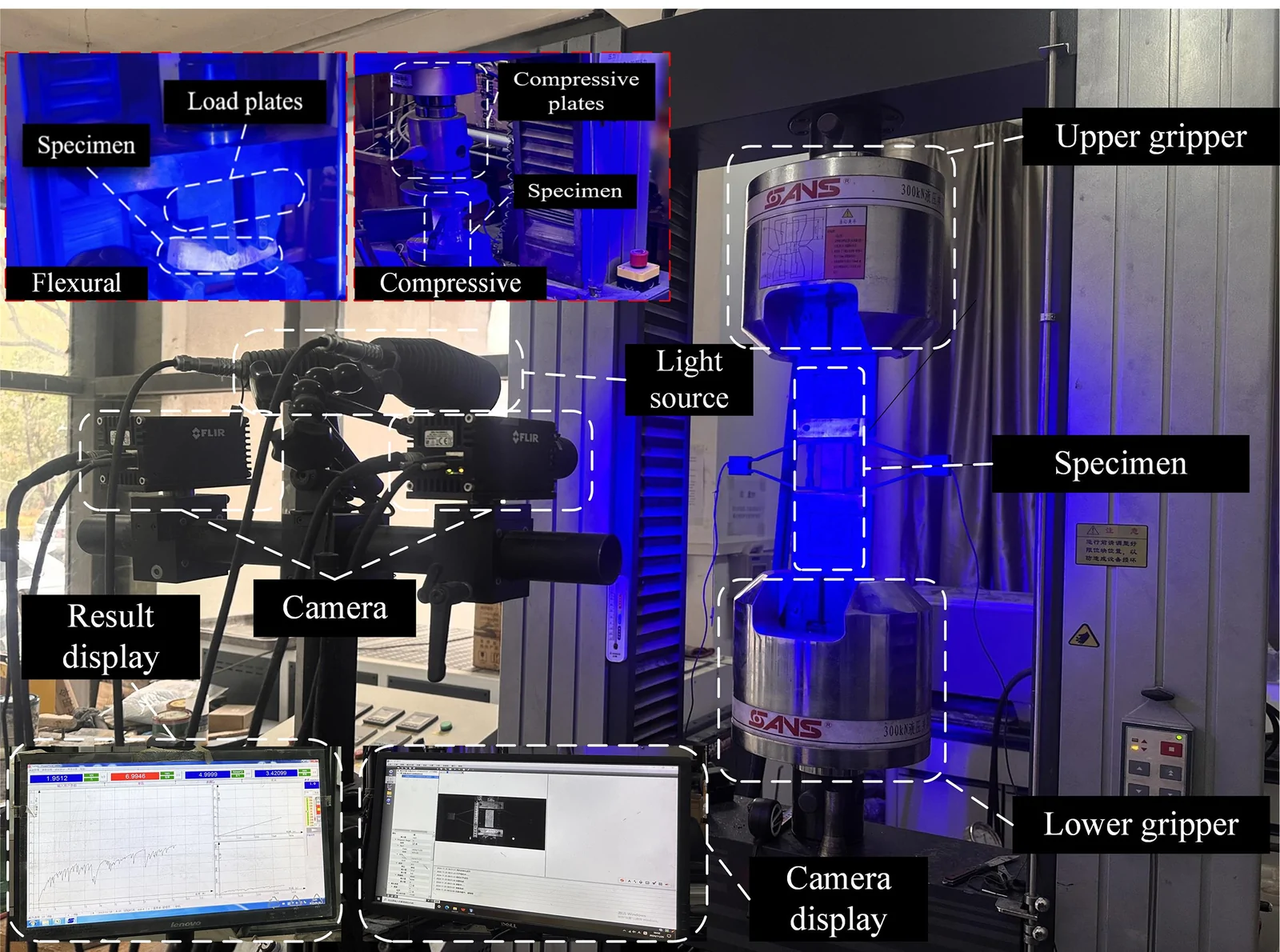
Experimental setup and instrument details.

The DIC system enabled full-field strain monitoring and real-time crack propagation analysis during flexural loading. The technique was used to evaluate strain localization, crack initiation, crack-bridging behavior, and deformation distribution within the ECC specimens. DIC is designed to capture an image every 3 seconds during the testing with a high image resolution of approximately 15 µm per pixel, as shown in Fig 2. To ensure experimental reliability and repeatability, multiple specimens were prepared and tested for each mixture configuration, and all specimens were cured under identical laboratory conditions before testing.

## Results and discussion

**Fig 3:**
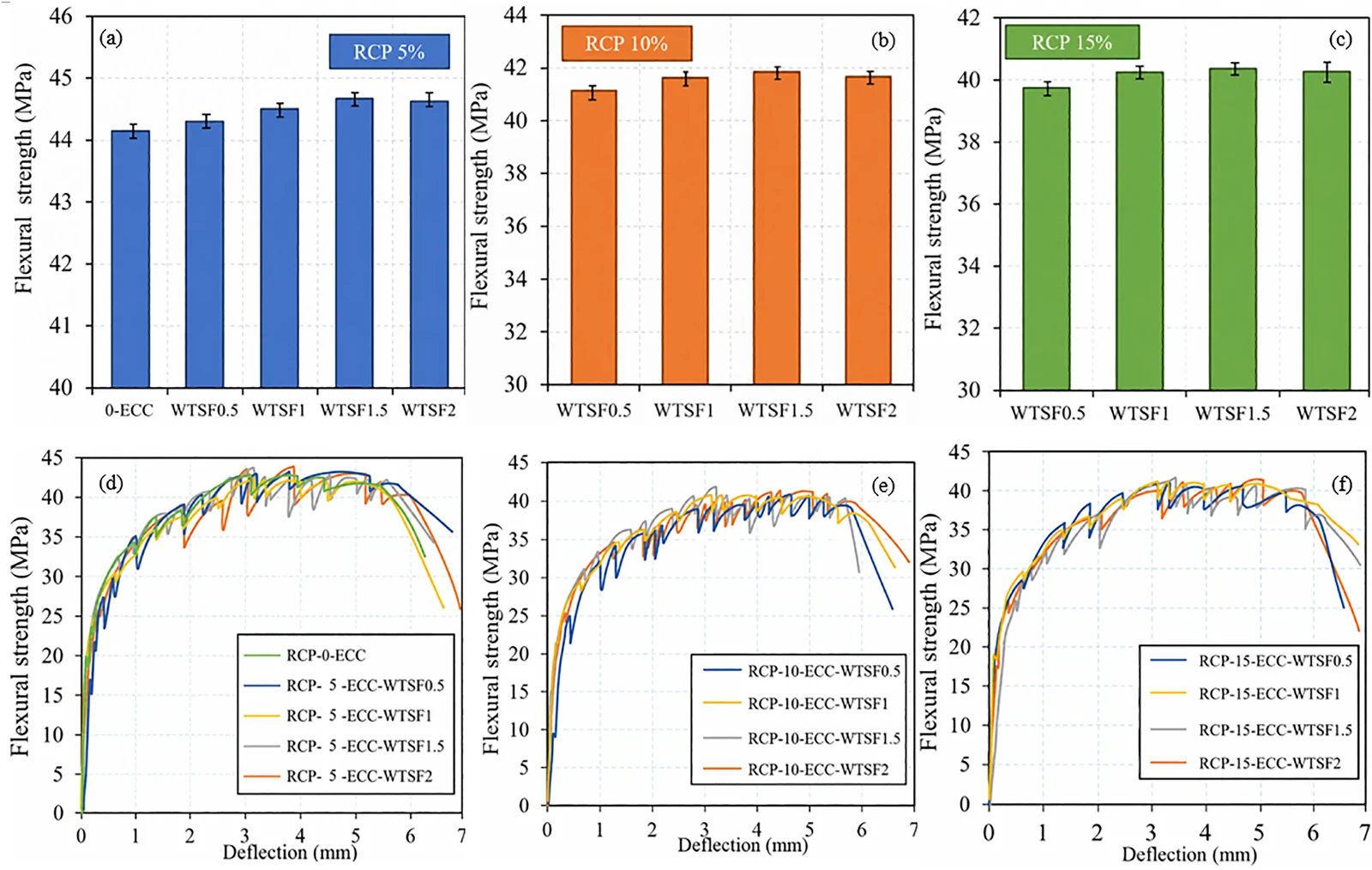
Flexural strength results with varying WTSF content and a constant 2% PE fiber content: (a) 5% RCP, (b) 10% RPC, (c) 15% RCP, (d) 5% RCP deflection curve, (e) 10% RCP deflection curve, and (f) 15% RCP deflection curve.

The effect of the incorporation of WTSF and PE fibers on the flexural strength and load-deflection behavior of sustainable ECC with different amount of RCP replacements are illustrated in Fig 3 and the results show that such incorporation has significant effects. The flexural strength at all replacement levels with RCP is consistently higher when the fiber dosage is increased from 0.5% to 2% for WTSF. The SF2% replacement is found to have the highest flexural strength of 44.5MPa, and SF1.5% replacement and SF1% replacement have flexural strength of 42.2MPa and 41.7MPa respectively at 5% replacement. The same trend is seen at a 10% replacement level, with the SF2% mix research showing 42MPa, followed by 41.5MPa for SF1.5% and 40.8MPa for SF1%. The flexural strength of SF2%, 40.5MPa, is the highest under 15% RCP replacement, showing this the fibers’ ability to alleviate the reduction of flexural strength due to increasing RCP content [30]. This behavior agrees to that previously found by other studies indicating that moderate RCP contents can help to densify the filler and enhance its hydration while too high contents can have detrimental effects on the matrix structure and on the mechanical properties. The load deflection curve also demonstrates that the higher SF dose results in better peak load, post peak ductility and crack-bridging ability, particularly at higher RCP levels [31]. The obtained flexural strength values are consistent with previous ECC studies incorporating recycled fibers and supplementary cementitious materials, where enhanced crack-bridging behavior and strain-hardening response contributed to improved post-cracking performance [17,18,26]. Compared with conventional fiber-reinforced cementitious composites, the present ECC mixtures demonstrated competitive flexural performance while incorporating environmentally sustainable recycled constituents.

**Fig 4:**
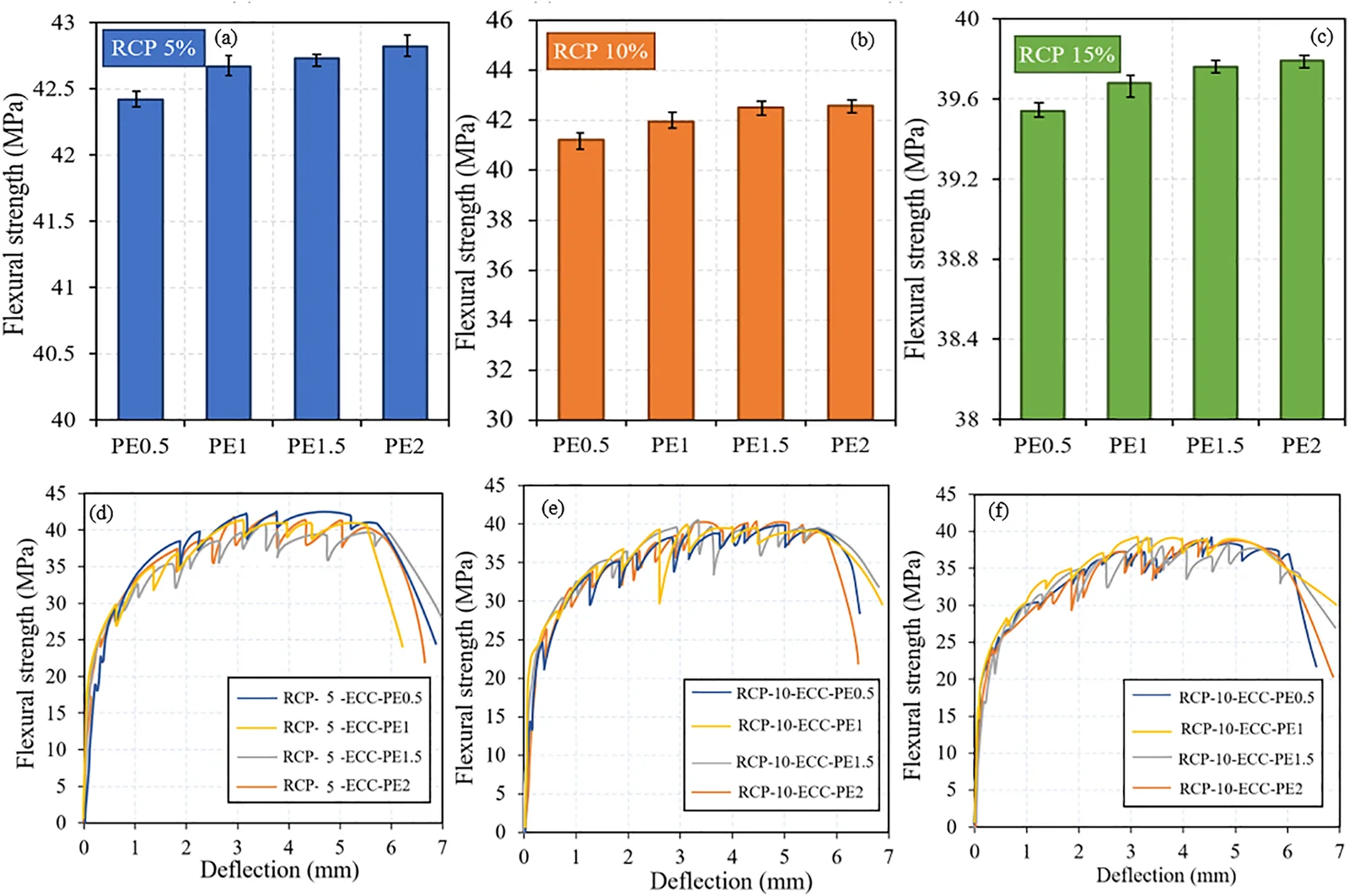
Flexural strength results with varying PE fiber content and a constant 2% WTSF content: (a) 5% RCP, (b) 10% RPC, (c) 15% RCP, (d) 5% RCP deflection curve, (e) 10% RCP deflection curve, and (f) 15% RCP deflection curve.

Similarly, in Fig 4 for PE fibers, increasing the fiber dosage from 0.5% to 2% combined with a constant 2% WTSF enhances the flexural strength. At 5% RCP replacement, PE2% achieves the maximum strength of 43.5MPa, followed by PE1.5% and PE1% with 42.8MPa and 42MPa, respectively. This trend continues at 10% RCP replacement, where PE2% reaches 41.8MPa, and at 15% RCP replacement, where PE2% reach the highest strength of 40MPa. The load-deflection behavior for PE fibers shows improved peak load and energy absorption with increasing fiber dosage, attributed to the fibers ability to delay crack propagation and enhance composite ductility [32]. It is observed that the strength loss associated with RCP replacement is mitigated by higher fiber contents (WTSF and PE fiber). However, the gradual reduction in flexural strength observed with increasing RCP content indicates that excessive replacement levels adversely influence matrix compactness and interfacial bonding characteristics. The presence of residual old mortar particles, increased porosity, and reduced cementitious reactivity in RCP may weaken the microstructure and reduce stress-transfer efficiency within the ECC matrix. Although higher fiber contents improved crack-bridging behavior and delayed crack propagation, the adverse influence of excessive RCP replacement could not be eliminated at higher replacement levels. Flexural strength is highest when both fibers are used at their maximum dosage of 2% of each, all RCP replacement levels. The observed strain localization and multiple-cracking behavior are comparable with previously reported DIC-based ECC investigations, where distributed microcracking and improved crack-bridging capacity were associated with enhanced ductility and energy dissipation characteristics [11,12].

### Microstructural analysis

#### Crack patterns and DIC results.

**Fig 5:**
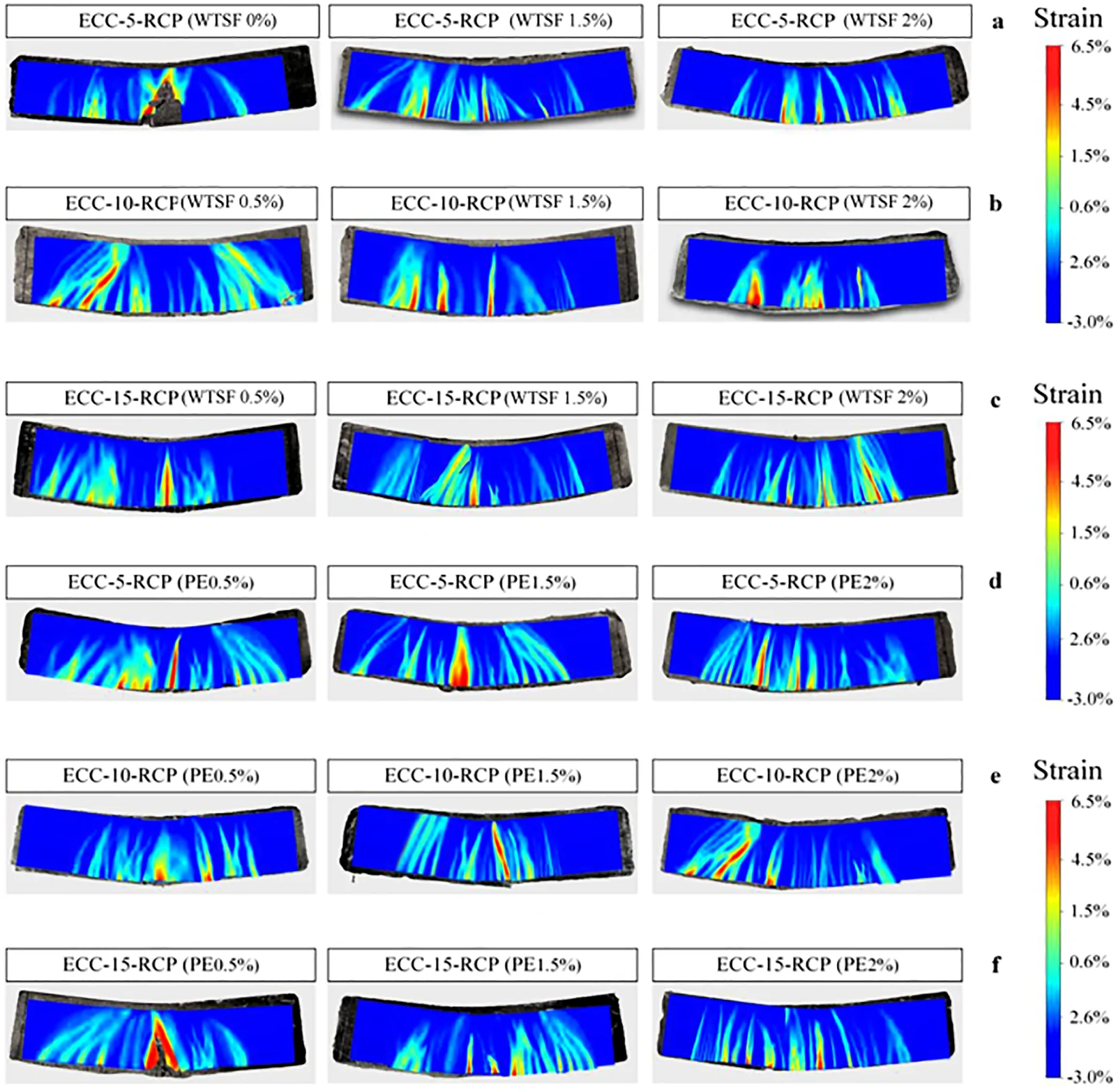
DIC strain contour maps illustrating the flexural behavior of sustainable ECC mixtures with varying WTSF, PE fiber and RCP contents: (a) 5% RCP with varying WTSF content, (b) 10% RCP with varying WTSF content, (c) 15% RCP with varying WTSF content, (d) 5% RCP with varying PE fiber content, (e) 10% RCP with varying PE fiber content, and (f) 15% RCP with varying PE fiber content.

Fig 5 presents DIC results for flexural strength under varying proportions of WTSF, PE fiber, and ECP in sustainable ECC. Increasing the WTSF and PE fiber content promoted crack initiation in the mid-span region at first but increasing fiber content after that strengthened strain redistribution and multiple development of microcracks throughout the specimen, resulting from the improved ability of the fibers to bridge cracks. More specifically, with higher fibre content (increasing from 0.5% to 2%), the strain is more distributed over the sample, implying better crack bridging and energy absorption capabilities. The flexural strength is increased by increased content of fiber also mitigates the ductility of crack growth and distributes stresses more effectively [33]. The transition from 5% to 15% RCP shows strain localization becoming more prominent at higher RCP levels, particularly in low fiber content. This behaviour is due to the decrease of the matrix integrity due to the increase of the RCP, which negatively influences load transfer mechanisms between fibres and matrix. Additional amount of fibres, however, can compensate this degradation by introducing the bridging effects in cracks [34].

To support the DIC interpretation, quantitative strain parameters were extracted from the contour maps and summarized in Table 4. These values help clarify the extent of strain localization, crack distribution, and crack-bridging behavior. Overall, higher WTSF and PE fiber contents promoted more distributed cracking and improved strain redistribution, while higher RCP replacement with lower fiber content showed more localized strain development.

**Table 4:** Quantitative DIC parameters, strain contour maps of sustainable ECC mixtures.

Mix ID	Approx. εmax (%)	Avg. tensile strain εavg (%)	SLF = εmax/εavg	Active crack bands	High-strain area ratio (%)
ECC-5-RCP-WTSF0%	6.5	2.8	2.3	4	4.9
ECC-5-RCP-WTSF1.5%	4.5	2.4	1.9	6	3.7
ECC-5-RCP-WTSF2%	4.5	2.5	1.8	4	3.8
ECC-10-RCP-WTSF0.5%	6.4	2.2	2.9	7	8.1
ECC-10-RCP-WTSF1.5%	4.5	2.6	1.7	3	4.8
ECC-10-RCP-WTSF2%	6.5	2.7	2.4	6	5.2
ECC-15-RCP-WTSF0.5%	4.5	2.2	2	6	5
ECC-15-RCP-WTSF1.5%	3.9	2.2	1.8	4	3.6
ECC-15-RCP-WTSF2%	6.5	2.5	2.6	10	5.5
ECC-5-RCP-PE0.5%	6.4	2.3	2.8	7	5
ECC-5-RCP-PE1.5%	6.5	2.8	2.3	6	5.9
ECC-5-RCP-PE2%	6.5	2.7	2.4	6	4.5
ECC-10-RCP-PE0.5%	4.1	2.2	1.9	6	3.6
ECC-10-RCP-PE1.5%	4.5	2.3	2	5	3.3
ECC-10-RCP-PE2%	6.4	2.6	2.5	4	4.7
ECC-15-RCP-PE0.5%	6.5	4.1	1.6	3	6.2
ECC-15-RCP-PE1.5%	3.5	1.9	1.9	7	3.1
ECC-15-RCP-PE2%	4.5	2.4	1.9	7	3.9

Note: εmax represents the maximum principal strain observed from the DIC contour map, εavg represents the average tensile strain within the high-strain region, and SLF = εmax/εavg is the strain localization factor. The high-strain area ratio was estimated using a strain threshold of approximately ε > 1.5%.

#### SEM analysis.

**Fig 6:**
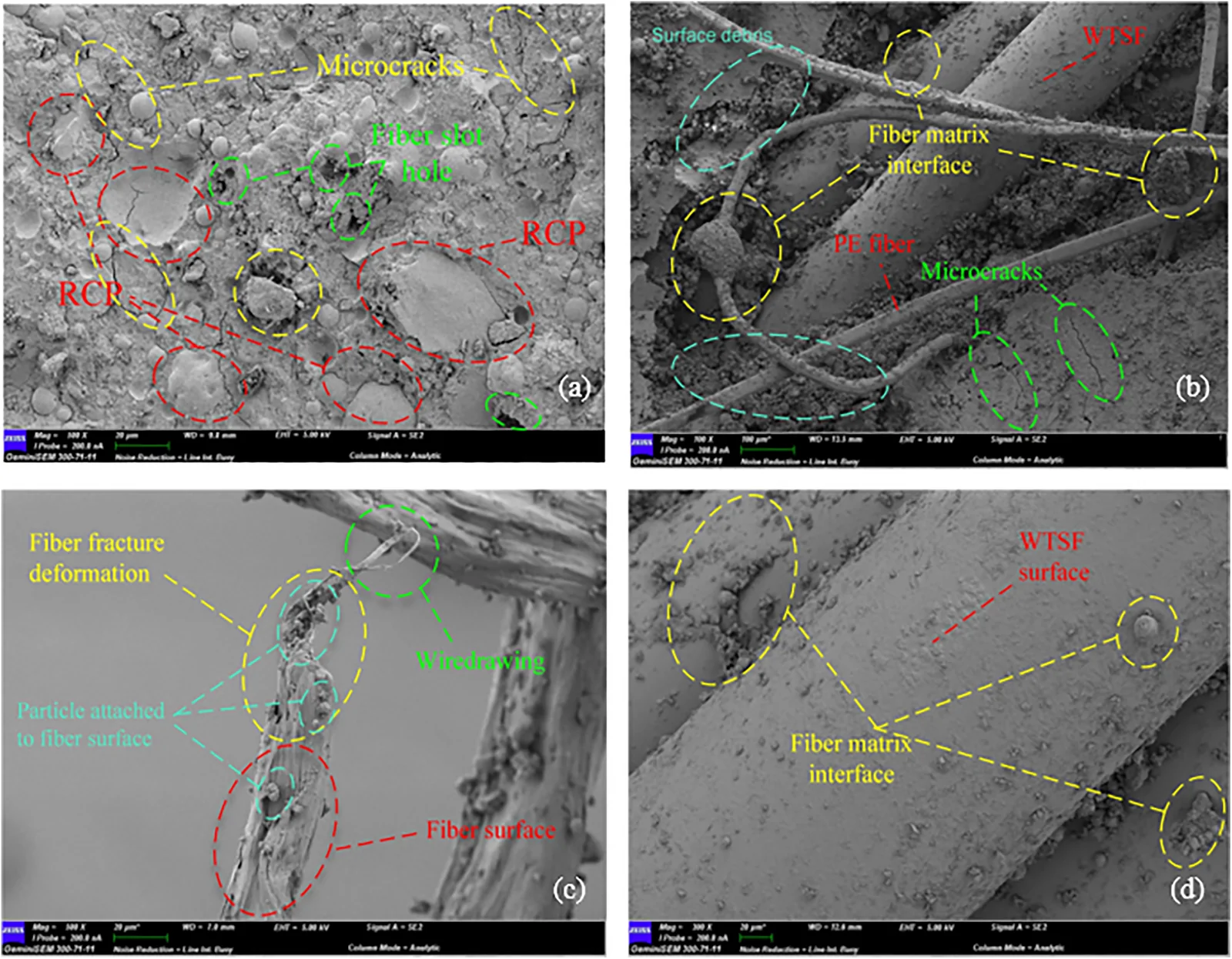
SEM images of sustainable ECC: (a) RCP particles, microcracks, and fiber slot holes; (b) fiber-matrix interface, PE fiber, WTSF, and microcracks; (c) fiber fracture deformation, wire drawing, and particles attached to the fiber surface; and (d) WTSF surface and fiber-matrix interface.

The microstructural behavior of sustainable ECC is shown in Fig 6, highlighting the bonding mechanisms and failure modes within the composite. Fig 6(a), shows RCP particles are well integrated into the matrix, with visible microcracks indicating potential stress localization matrix discontinuities, and relatively porous regions due to the partial cement replacement by RCP. The fiber slot holes in the figure show effective stress transfer between fibers and the matrix, while porosity in the matrix reflects the area of incomplete compaction or hydration, which could impact mechanical performance [35]. In Fig 6(b), the interaction between the matrix and fibers is emphasized, with a strong fiber-matrix interface with good adhesion, which is important for effective stress dissipation and tensile strength improvement [36]. The microcracks surrounding fibers highlight stress localization during tensile loading, while surface debris and hydration byproducts further indicate robust interaction between fibers and the matrix.

Fiber deformation and fracture are evident in Fig 6(c), demonstrating the composite strain-hardening capabilities and energy dissipation during mechanical loading. The attachment of hydration byproducts on the fiber surface suggests strong bonding, while the fiber withdrawal marks reveal that fibers were actively engaged in absorbing tensile stresses before failure. Moreover, Fig 6(d) highlights the rough surface of WTSF and its strong integration within the matrix. The clear fiber-matrix interface and residual matrix particles on the fiber surface indicate excellent mechanical interlocking, which enhances stress transfer and reinforces the composite [37]. Overall, the SEM analysis demonstrates that the use of RCP effectively reduces cement content while maintaining bonding strength, and inclusion of WTSF significantly improves tensile performance by facilitating crack bridging and strain-hardening behaviour.

## Life cycle assessment (LCA)

The LCA study was conducted using open-source life cycle assessment software with the ecoinvent database [32,33]. In this study, the functional unit for the Life Cycle Assessment (LCA) was defined as 1 m^3^ of ECC mixture to enable a consistent environmental comparison between conventional ECC and sustainable ECC mixtures incorporating recycled concrete powder (RCP) and waste tire steel fiber (WTSF). The adopted cradle-to-gate system boundary includes raw material extraction, material processing, transportation, recycling activities, and manufacturing-stage operations associated with ECC production. However, construction-stage activities, operational service life, maintenance, demolition, and end-of-life disposal stages were excluded from the present assessment. This software can create a scenario based on the processing of ecoinvent databases and assessment methods. The quality and reliability of the database are essential for the LCA model because the process results depend on the inputs and outputs of each process step [38]. There are four stages in an LCA study: first, the definition of the goal, which defines the objective of the study; second, the inventory analysis, which covers the processes and materials used across the whole cycle; third, impact evaluation, which quantifies the selected impact categories required by the study; and finally, interpretation, which involves analyzing and explaining the results. In LCA studies, using different software can result in different outcomes for the same data or procedure. The main reason is inconsistencies among the databases and how they are collected and compile. However, with appropriate control procedures and clear inclusion and exclusion criteria, these differences are unlikely to significantly affect the outcomes [39]. The working principle of this software is to assess the inventoried flows by applying characterization factors, as shown in Equation (1), mainly for the CO_2_-equivalent greenhouse gas emissions.


$$\begin{array}{c} I_{k}=\sum_{i=\text{1}}^{n}\left(F_{i}\times CF_{i,k}\right) \end{array}$$ (1)

In the formula, *I*_*k*_ indicates the impact category, where *k* represents the global warming potential (GWP), which is used to quantify climate change impacts. This is Followed by *Fi,* which represents the substance flow amount for emissions such as CO_2_ and CH_4_, and finally *CF*_*i,k*_*,* which is the characterization factor for substance *i* in impact category *k*. The number flows is denoted by *n* in the inventory. The reliability of the ecoinvent database used in this study is justified by its strong reputation and widespread recognition among global life cycle inventory (LCI) databases. The inventory is developed by trained experts and provides reliable, accessible, and transparent information across thousands of LCI datasets [40]. There are approximately 4087 processes, which are mainly related to human activities, and the system is categorized by regions and grouped into economic and product type sections [41].

The ReCiPe2016 (V1.03) midpoint (H) has been used for the impact categories as the hierarchical perspective on this selection based on both short-term and long-term emission effects to bring balanced in the investigation. One of the key impact categories in the ReCiPe2016 is greenhouse gases (GHGs) which leads to the global warming potential (GWP) in the climate change section as presented in Equation (2) [42].


$$\begin{array}{c} \text{GWP}{\;}_{t}=\sum_{i=\text{1}}^{n}\left(M_{i}\times {CF}_{i,t}\times {\text{e}}^{-\lambda_{i}t}\right) \end{array}$$ (2)

Equation (3) is expressed in terms of gas mass, indicated as *Mi* (mass of gas *i*), and ${CF}_{i,t}$ which represents the gas characterization factor, where *t* denotes the time horizon. The *t*erm ${\text{e}}^{-\lambda_{i}t}$ Indicate the decay function for gas *i* over the time horizon *t*. Another impact category included is Fossil Depletion Potential (FDP), reported in oil-equivalen*t* units, which focuses on resource depletion by the comparison between crude oil and fossil fuel energy [43], as presented in Equation (3).


$$\begin{array}{c} \text{FDP}=\sum_{i=\text{1}}^{n}\left(R_{i}\times {EC}_{i}\times {SF}_{i}\right) \end{array}$$ (3)

The $R_{i}$ In the equation, represents the amount of fossil resource *i,* and ${EC}_{i}$ indicates the energy content per unit mass (MJ/kg). Finally, the scarcity factor, ${SF}_{i}$ is included in the equation and depends on resource availability and depletion rates. Moreover, human toxicity is included and measured in kg 1,4-dichlorobenze equivalents (1,4 DCB-eq) to quantify carcinogenic and non-carcinogenic effects on human health in terms of disease incidence [44,45].


$$\begin{array}{c} \text{FEP}=\sum_{i=\text{1}}^{n}\left(N_{i}\times {CF}_{i,\text{FEP}}\times T_{i}\right) \end{array}$$ (4)

In the Freshwater Eutrophication Potential (FEP) formula, as presented in Equation (18), different factors have been considered. $N_{i}$, indicates the nutrient content within the freshwater flow. In addition, Marine Eutrophication Potential (MEP) is included and reported in the kg-nitrogen-equivalents, reflecting impacts such as algal blooms and oxygen depletion. Another category included Ozone Depletion Potential (ODP), expressed in kg CFC-11-equivalents, which quantifies stratospheric ozone loss. Furthermore, Terrestrial Acidification potential (TAP20) is included, representing acid deposition caused by pollutants such as NOx, NH_3_, and SO_2,_ which can harm terrestrial ecosystems [46].

ODP is calculated using Equation (5), where the characterization includes $M_{i}$, and the characterization factor ${CF}_{i,ODP}$, and $L_{i}$, represents the lifetime factor for substance *i.* In addition, impact categories related to metal and mineral resource depletion are included [47]. The reason for including these categories is that resource extraction and depletion can indirectly influence other environmental impacts, including ODP emission and ecosystem degradation. This category is measured in kg copper-equivalent (kg Cu-Eq), which reflects challenges of declining ore grades. Furthermore, Particulate Matter Formation (PMFP) is included to account for fine particulate emission (e,g., PM2.5) from industrial processes, which pose risks to human health and the environment [48].


$$\begin{array}{c} \text{ODP}=\sum_{i=\text{1}}^{n}\left(M_{i}\times {CF}_{i,ODP}\times L_{i}\right) \end{array}$$ (5)

$$\begin{array}{c} \text{PMFP}=\sum_{i=\text{1}}^{n}\left(M_{i}\times {CF}_{i,\text{PMFP}}\times C_{i}\right) \end{array}$$ (6)

In Equations (5) and (6), $M_{i}$ represents the mass of precursor substance i, while $CF_{\left(i,PMFP\right)}$ denotes the characterization factor for particulate matter formation potential. In addition, $C_{i}$ represents the conversion efficiency used to account for the transformation of precursor emissions into particulate matter. The related health impacts are assessed using DALYs (disability-adjusted life years), which quantify the burden of particulate matter on human health [49].

### LCA of cement vs RCP

Table 5 highlights the resource-intensive nature of producing 1 kg of Portland cement. The primary input, clinker (955g), is produced through the energy-intensive calcination of limestone, releasing significant CO_2_ emissions. The process also relies on electricity (31.7g) to operate machinery, reflecting the energy demand of cement manufacturing. To regulate the setting time of cement, gypsum (45g) is added during the grinding stage, where ethylene glycol (0.19g) serves as a chemical aid to enhance grinding efficiency. Additionally, low-alloyed steel (0.11g) is used in machinery and equipment, contributing indirectly to energy consumption and emissions during its production. While minimal, the input for the cement factory (5.36 x 10^-8^g) represents operational components within the manufacturing process. Overall, the data underscores the energy demands and environmental burdens inherent to cement production.

**Table 5:** 1 kg cement Portland cement production process.

Step/Process Name	Input (Inflow)	Amount	Unit
Cement Production	Cement factory (input material)	5.36 x 10^−8^	g
	Clinker (Input material)	955	g
	Electricity, medium voltage (for equipment operation)	31.7	g
	Ethylene glycol (for chemical processes)	0.19	g
	Gypsum, mineral (input material)	45	g
	Steel, low-alloyed (for machinery and equipment)	0.11	g

Table 6 shows the inputs and outputs for several stages of a process involved with construction. The inputs for this step are diesel for a building machine (0.0438MJ), hydraulic digger excavation (5.56 x 10−4 m3) and low voltage electricity (0.00125 kWh). The production of inert waste: 88.4g and 312g, PM2.5: 0.0166g are the outputs for this step.

**Table 6:** RCP production process from site preparation to production.

Step/Process Name	Input (Inflow)	Amount	Unit	Output (Outflow)	Amount	Unit
1. Site Preparation	Diesel, for the machine	0.0437	MJ	Inert waste	88.4	g
	Hydraulic Excavation	5.56 x 10^−4^	m3	Inert waste	312	g
	Low-voltage electricity	0.00125	kWh	Particulates, < 2.5 µm	0.0166	g
2. Waste Treatment	Waste concrete gravel	1	kg	Particulates, > 10 µm	0.0835	g
3. Waste Disposal	Sorting facility	1.00 x 10^−10^	Item	Particulates, > 2.5 µm, and < 10µm	0.0634	g

During waste treatment, waste concrete gravel (1 kg) is treated and particulates larger than 10 µm (0.0835g) are emitted. Finally, in the waste disposal step, one type of construction waste treatment facility is a sorting facility for construction waste, which produces particulates with a range of 2.5 µm to 10 µm (0.0634g). The environmental impacts of different cement types, traditional cement versus RCP, are presented in Table 7 and are summarized below. Table 7 shows an extensive comparison of the impacts of the use of traditional cement and RCP as a cement replacement for both, across a wide range of environmental impacts. The outcomes can be considered as significant environmental findings in replacing cement with RCP. For example, the land stress reduces significantly for ALOP, with RCP requiring only 2.75 x 10−4 m2a in comparison to cement which requires 3.82 x 10−4 m^2^a. This reduction stems from RCP’s production, which utilizes waste concrete instead of extracting and processing raw materials like limestone and clay required for cement manufacturing. In terms of climate change (GWP20). At the individual material level, RCP shows approximately 99% lower climate change impact than cement, indicating its potential as a low-carbon cement replacement material. This reduction is mainly because cement production is highly energy-intensive and generates substantial CO_2_ emissions during clinker production, calcination, and fuel combustion [50]. In contrast, RCP is derived from recycled concrete waste and requires comparatively lower processing energy. However, this reduction should be interpreted as a material substitution-level benefit, while the environmental reduction in the complete ECC mixture depends on the replacement ratio, total binder content, fiber type, and other mixture constituents.

**Table 7:** Environmental Impact Comparison of Cement vs RCP.

Impact category	Cement	RCP	Unit
Agricultural land occupation (ALOP)	3.82 x 10^−3^	2.75 x 10^−4^	m^2^a
Climate change (GWP20)	943 (849-1037)^*^	9.41 *(8.47-10.37)* ^*^	g CO2-Eq
Fossil depletion (FDP)	125 *(113-138)* ^*^	3.45 *(3.11-3.80)* ^*^	g oil-Eq
Freshwater ecotoxicity (FETP100)	3.43	0.566	g 1,4-DCB-Eq
Freshwater eutrophication (FEP)	0.228	2.42 x 10^−3^	g P-Eq
Human toxicity (HTP100)	22.1	1.14	g 1,4-DCB-Eq
Ionising radiation (IRP_I)	0.614	0.285	g U235-Eq
Marine ecotoxicity (METP100)	2.72	0.424	g 1,4-DCB-Eq
Marine eutrophication (MEP)	0.924	2.81 x 10^−2^	g N-Eq
Metal depletion (MDP)	0.162	1.52 x 10^−3^	g Fe-Eq
Natural land transformation (NLTP)	3.64 x 10^−2^	3.27 x 10^−3^	m^2^
Ozone depletion (ODPinf)	1.32 x 10^−5^	1.36 x 10^−6^	g CFC-11-Eq
Particulate matter formation (PMFP)	1.23	0.112	g PM10-Eq
Photochemical oxidant formation (POFP)	3.11	8.51 x 10^−4^	g NMVOC
Terrestrial acidification (TAP20)	2.1	4.81 x 10^−2^	g SO2-Eq
Terrestrial ecotoxicity (TETP100)	1.43 x 10^−2^	9.6 x 10^−4^	g 1,4-DCB-Eq
Urban land occupation (ULOP)	43.2	0.72	m^2^a
Water depletion (WDP)	1.04	1.74 x 10^−2^	m^3^

Note: *(±10% sensitivity ranges).

**Fig 7:**
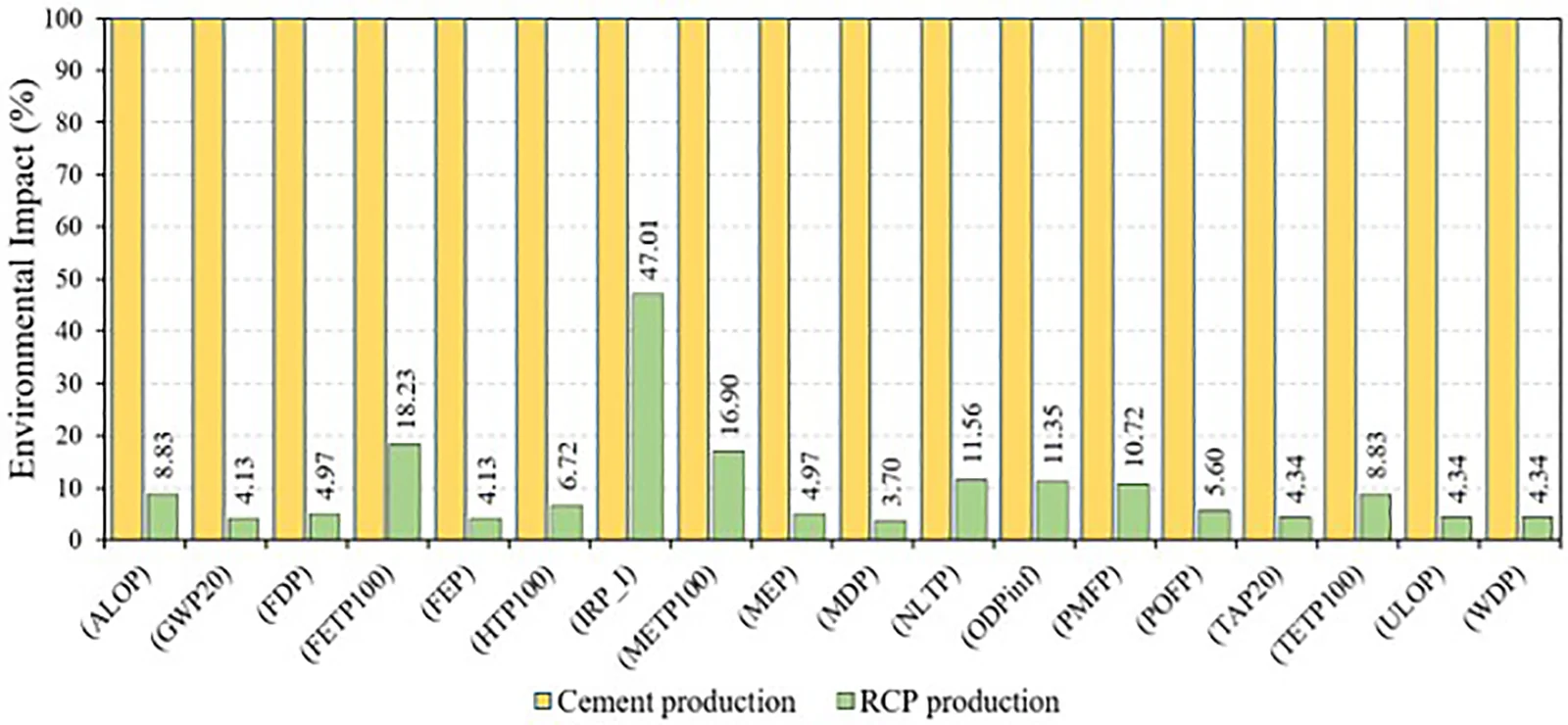
Environmental impact of cement and RCP production.

Additionally, FDP is reduced by over 97%, demonstrating the minimal reliance on fossil resources with RCP. Similar trends are observed for freshwater and marine ecotoxicity, where toxic emissions into water bodies are significantly minimized due to the reduced energy and material input in RCP production. HTP100 also show a 94% reduction with RCP, further emphasizing its reduced health risks. Moreover, RCP decreases ODPinf by over 89% and drastically lowers impacts on terrestrial and eutrophication. The most striking reduction is seen in ULOP, where RCP requires only 0.72 m^2^a, an impressive 98% reduction compared to cement’s 43.2 m^2^a as shown in Fig 7. Finally, WDP drops dramatically, with RCP consuming just 0.0174 m^3^ versus cement’s 1.04 m^3^, highlighting its water sustainability.

Comparison of Ordinary Portland Cement (OPC) with RCP has been reported in several studies. Li et al. [51] reported that OPC emits 0.83 kg-CO_2_/kg, whereas RCP significantly reduces emissions to 0.004 kg-CO_2_/kg. Similarly, Xiao et al. [52] quantified emissions in terms of carbon equivalent, with OPC at 1095 kg-Ce/t and RCP at 48.8 kg-Ce/t indicating a substantial reduction. In other studies, Tan et al. [53] observed that emission from OPC reached 0.93 kg-CO_2_/kg, while RCP emission were reduced to 0.24 kg-CO_2_/kg. Additionally, He et al. [54] reported OPC at 930 kg-CO_2_/t, compared to RCP emission of 19.6 kg-CO_2_/t. Therefore, these studies collectively demonstrate that replacing OPC with RCP significantly reduces CO_2_ emissions. It should be noted that these environmental impacts represent material-level production comparisons only and do not directly correspond to the overall ECC mixture-level environmental reductions discussed later in the manuscript.

### LCA of steel fiber vs waste rubber steel fiber

Table 8 present the inflow and outflow details of the steel fiber production process, capturing each step from raw material preparation to final heat treatment. The process begins with raw material preparation, where 42.8g of unalloyed steel is cleaned to produce steel rods, and 0.0316g of chemicals are used for pickling, resulting in the removal of scale or rust as waste. During the wire-drawing process, 1000g of steel is rolled and drawn to decrease the diameter of the steel and 2.58g of lubricating oil is supplied to facilitate the process, with heat loss and friction loss as the outputs. The intermediate annealing process then comes into play, in which 1000g of drawn steel wire is heated by 392g of natural gas (MJ) to create ductility, along with heat exhaust, which is the by-product. The next step in the process is the coating and surface treatment process, where 1000g of annealed wire is coated with protective material like zinc (galvanizing) or plastic to stop corrosion or make it more durable. Depending on the coating method used, a certain amount of waste chemical may result [55].

**Table 8:** Life cycle inventory for production of 1 kg industrial steel fiber inflow and outflow.

Step/Process Name	Input (Inflow)	Amount	Unit	Output (Outflow)	Amount	Unit
1. Raw Material Preparation	Steel, unalloyed (for billets or wire rods)	1000	g	Cleaned steel rods	–	–
	Chemicals (inorganic & organic) (for pickling)	0.0316	g	Removed scale/rust (waste)	–	–
2. Wire Drawing	Rolling mill & wire drawing (steel) (raw material)	1000	g	Drawn steel wire (reduced diameter)	1000	g
	Lubricating oil (for drawing)	2.58	g	Heat/friction losses	–	–
3. Intermediate Annealing	Drawn steel wire (input material)	1000	g	Annealed wire (improved ductility)	1000	g
	Heat (natural gas) (for annealing process)	392	g (MJ)	Heat exhaust	–	–
4. Coating and Surface Treatment	Annealed wire (input material)	1000	g	Coated wire	1000	g
	Zinc (for galvanizing) or Plastic (for coating)	–	g	Waste chemicals (if applicable)	–	–
5. Cutting and Spooling	Coated wire (spooled or cut)	1000	g	Finished wire product (on reels or cut lengths)	1000	g
	Corrugated board box (for packaging)	4.71E-09	g	Waste scraps (if any)	–	–
6. Final Heat Treatment	Finished wire (input material)	1000	g	Final wire product (desired mechanical properties)	1000	g
	Heat (natural gas) (for final heat treatment)	392	g (MJ)	Heat exhaust	–	–

In the cutting and spooling step, the coated wire, weighing 1000g, is either spooled or cut into desired lengths, with a negligible input of corrugated board box (4.71 x 10^−9^ g) for packing, while minimal waste scraps may occur during processing. Finally, the final heat treatment step uses 392 g (MJ) of natural gas to achieve the required mechanical properties for 1000g of finished wire, producing heat exhaust as an unavoidable output [56].

The extraction process of waste rubber tires for their embedded steel fiber extraction process and its energy flow is presented in Table 9. The process involves five steps begins with the collection of waste tires from the disposal sites, after the collection, the waste tires undergo shredding or cryogenic processing which uses 392g (MJ) of natural gas and result in scrap rubber. The shredding process of 1000g of waste tires results in 500g of shredded rubber mixed with embedded steel wire, with 2.58g of lubrication oil used for equipment, producing a small amount of waste oil as an outflow.

**Table 9:** Life cycle inventory for production of 1 kg WTSF inflow and outflow.

Step/Process Name	Input (Inflow)	Amount	Unit	Output (Outflow)	Amount	Unit
1. Tire Collection & Preprocessing	Waste rubber tires	1000	g	Scrap rubber (post-removal of non-metallic parts)	–	–
	Heat (natural gas) (for shredding/cryogenic processing)	392	g (MJ)	Heat exhaust (from shredding process)	–	–
2. Shredding & Separation	Waste rubber tires (input material)	1000	g	Shredded rubber and steel wire	500	g
	Lubricating oil (for equipment lubrication)	2.58	g	Waste oil (small fraction)	–	–
3. Magnetic Separation	Shredded rubber and steel wire (input material)	500	g	Extracted steel wire	100	g
	Electricity (for magnetic separation machinery)	0.14	kWh	Heat (due to electrical machinery)	–	–
4. Rubber Removal	Steel wire (input material)	100	g	Cleaned steel wire	100	g
	Chemicals (for rubber removal)	0.0316	g	Rubber waste (after chemical process)	–	–
5. Final Sorting & Packaging	Cleaned steel wire (input material)	100	g	Packaged steel wire (ready for recycling or reuse)	100	g
	Packaging materials (e.g., plastic film, cardboard)	4.71 x 10^−9^	g	Waste packaging materials	–	–

The separation process of steel wire from the waste-tired use magnetic separator, in which 500g of shredded tried can extract 100g of WTSF using only 0.14 kWh of electricity. As multiple stages are involved in this process, from magnetic machinery normally used for minor heating due to the electrical usage outflow. Finally, the rubber removal as it is the focus to extract the steel fiber from the rubber. Here, 100g of steel wire treated with 0.0316g of chemicals to remove residual rubber, leading to clean steel wire as the final product and rubber waste as a byproduct. Lastly, in the final sorting and packaging step, 100g of cleaned steel wire is prepared for recycling or reuse, using a negligible amount (4.71 x 10^-9^g) of packaging materials, such as plastic film or cardboard.

The environmental impacts of conventionally produced steel fiber and waste tire-derived steel fiber are compared across various impact categories, highlighting the sustainability advantages of using recycled materials, as shown in Table 10. In terms of GWP20, steel fiber production generates 1350 g CO_2_-Eq, whereas waste tire steel fiber results in significantly lower emission at 348 g CO_2_-Eq, representing a 74% reduction. Similarly, in FDP, waste tire steel fiber exhibits a much smaller footprint of 70.7 g oil-Eq, compared to 236 g oil-Eq for traditional steel fiber, indicating a more efficient fossil resource.

**Table 10:** Environmental Impact Comparison of steel fiber vs waste rubber steel fiber.

Impact Category	Steel Fiber	Waste Tire Steel Fiber	Unit
Agricultural Land Occupation (ALOP)	8.76 x 10−1	9.15 x 10−2	m^2^a
Climate Change (GWP20)	1350 (1215–1485)*	348 (313–383)*	g CO_2_-Eq
Fossil Depletion (FDP)	236 (212–260)*	70.7 (63.6–77.8)*	g oil-Eq
Freshwater Ecotoxicity (FETP100)	27.4	9.51	g 1,4-DCB-Eq
Freshwater Eutrophication (FEP)	1.44	3.43 × 10^−2^	g P-Eq
Human Toxicity (HTP100)	221	2.68	g 1,4-DCB-Eq
Ionising Radiation (IRP_I)	19.9	9.23	g U235-Eq
Marine Ecotoxicity (METP100)	13.2	8.59	g 1,4-DCB-Eq
Marine Eutrophication (MEP)	1.18	0.136	g N-Eq
Metal Depletion (MDP)	0.116	5.49 × 10^−2^	g Fe-Eq
Natural Land Transformation (NLTP)	5.31 x 10−5	1.73 x 10−5	m^2^
Ozone Depletion (ODPinf)	1.99 x 10−5	3.27 x 10−5	g CFC-11-Eq
Particulate Matter Formation (PMFP)	6.22 × 10^3^	200	g PM₁₀-Eq
Photochemical Oxidant Formation (POFP)	3.45 × 10^3^	696	g NMVOC
Terrestrial Acidification (TAP20)	5.11 × 10^3^	309	g SO_2_-Eq
Terrestrial Ecotoxicity (TETP100)	4.23 × 10^−2^	1.8	g 1,4-DCB-Eq
Urban Land Occupation (ULOP)	1.62 x 10−2	1.35 x 10−3	m^2^a
Water Depletion (WDP)	0.455	1.32	g H_2_O (m^3^)

Note: *(±10% sensitivity ranges).

The FETP100 and METP100 impacts are also substantially reduced for waste tire steel fiber, with values of 9.51g and 8.59g 1,4-DCB-Eq, respectively, compared to 27.4g and 13.2g for conventional steel fiber. These reductions are due to the avoidance of energy-intensive steel production processes, which typically release toxic emissions into aquatic systems.

**Fig 8:**
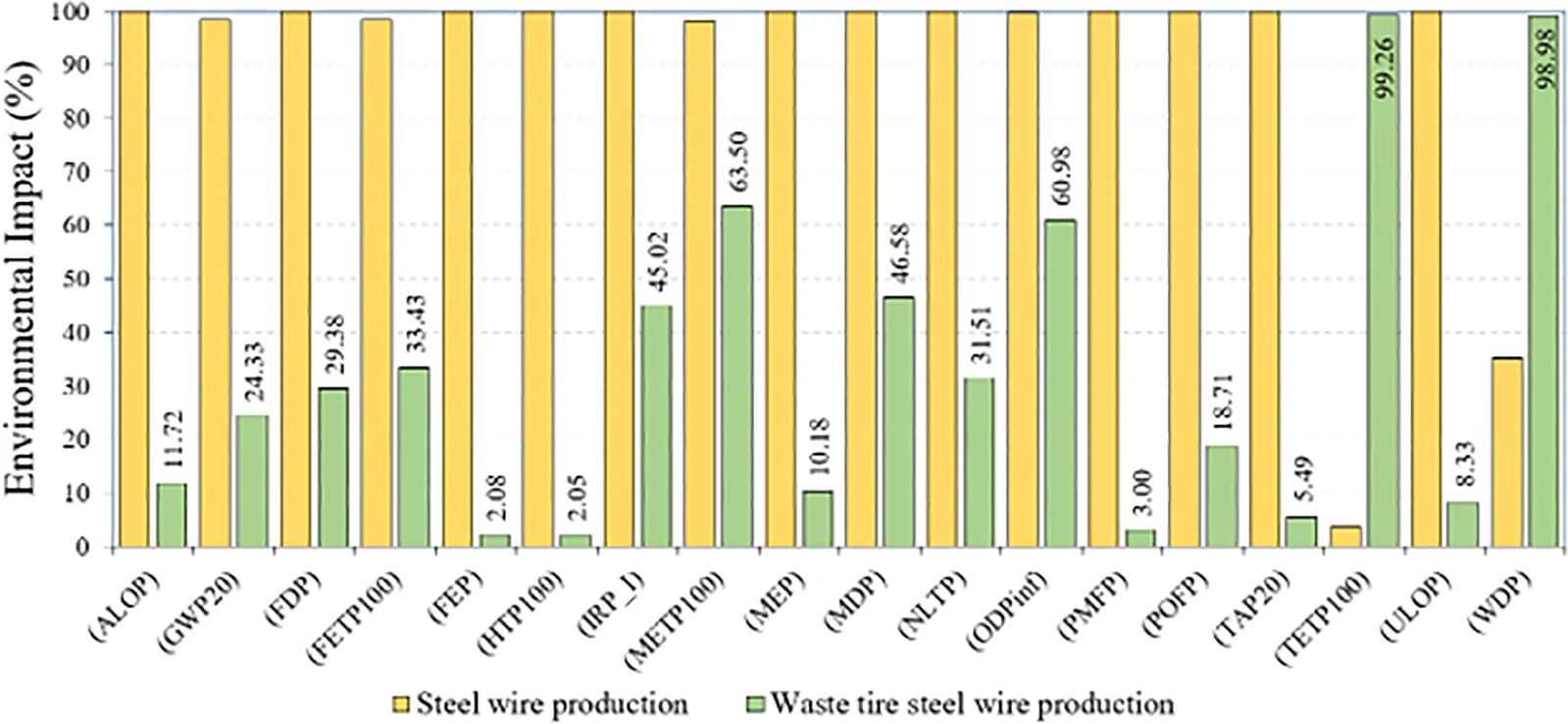
Environmental impacts of steel fiber vs waste tire steel fiber production.

A significant reduction is noticed in HTP100, with 2.68 g w,4-DCB-Eq as compared to the steel fiber as seen in Fig 8. As these results shows an improvement that underscores the production of chemical exposure with the recycled processes of waste materials. Other categories also show a significant difference. TAP20 and PMFP are much lower for waste tire steel fiber (309g SO_2_-Eq and 200g PM_10_-Eq) compared to steel fiber (5110g SO_2_-Eq and 6220 g PM_10_-Eq), highlighting its environmental efficiency. However, ODPinf slightly increases for waste tire steel fiber 3.27 x 10^-5^g CFC-11-Eq, versus steel fiber 1.99 x 10^-5^g CFC-11-Eq, likely due to chemical treatments. Land use metric favor waste tire steel fiber, requiring less urban and agricultural land compared to steel fiber. However, ULOP and WDP is higher, indicating room for improvement.

### LCA of sustainable ECC vs conventional ECC

In the LCA study, using recycled materials, where RCP and WTSF were used to partially replace cement and industrial steel fiber, resulted in a notable reduction in environmental impact, particularly when 2% WTSF and 2% PE fibers were used. Table 11 presents a sustainable ECC mixture with 5% RCP replacement of cement, 2% of WTSF, and 2% of PE fiber, which shows a significant reduction in environmental benefits. The results for the ALOP category show a reduction from 0.887 m^2^a to 0.842 m^2^a, which may appear small; however, with large-scale use and higher production volume, this reduction can become significant. This reduction is attributed to replacing cement with RCP, which lowers land use. Similarly, the GWP20 result shows the same trend, decreasing from 1365 g CO_2_-Eq to 1293.5 g CO_2_-Eq for the sustainable mixture. As is well known, cement is a major contributor to CO_2_ emissions, and replacing cement with recycled material reduces the overall carbon footprint.

**Table 11:** Environmental Impact Comparison of ECC vs Sustainable ECC.

Impact Category	Conventional ECC (0% RCP)	Sustainable ECC (5% RCP)	Sustainable ECC (10% RCP)	Sustainable ECC (15% RCP)	Unit
Agricultural Land Occupation (ALOP)	0.887	0.84	0.80	0.75	m^2^a
Climate Change (GWP20)	1365 (1228–1501)*	1294 (1164–1423)*	1187 (1068–1306)*	1135 (1022–1249)*	g CO_2_-Eq
Fossil Depletion (FDP)	238 *(214–262)*	223 *(201–245)*	206 *(185–226)*	192 *(173–211)*	g oil-Eq
Freshwater Ecotoxicity (FETP100)	27.23	26.41	24.58	24.26	g 1,4-DCB-Eq
Freshwater Eutrophication (FEP)	1.48	1.37	1.31	1.17	g P-Eq
Human Toxicity (HTP100)	220.6	206.32	201.13	182.26	g 1,4-DCB-Eq
Ionising Radiation (IRP_I)	19.81	19.43	18.15	18.22	g U235-Eq
Marine Ecotoxicity (METP100)	13.31	12.53	12.12	11.15	g 1,4-DCB-Eq
Marine Eutrophication (MEP)	1.22	1.14	1.03	0.96	g N-Eq
Metal Depletion (MDP)	0.113	0.11	0.10	0.10	g Fe-Eq
Natural Land Transformation (NLTP)	5.20 x 10^−5^	5.20 x 10^−5^	4.80 x 10^−5^	4.60 x 10^−5^	m^2^
Ozone Depletion (ODPinf)	2.00 x 10^−5^	1.90 x 10^−5^	1.80 x 10^−5^	1.70 x 10^−5^	g CFC-11-Eq
Particulate Matter Formation (PMFP)	6.27 × 10^3^	5.86 × 10^3^	5.52 × 10^3^	5.08 × 10^3^	g PM₁₀-Eq
Photochemical Oxidant Formation (POFP)	3.43 × 10^3^	3.24 × 10^3^	3.05 × 10^3^	2.86 × 10^3^	g NMVOC
Terrestrial Acidification (TAP20)	5.20 × 10^3^	4.79 × 10^3^	4.70 × 10^3^	4.13 × 10^3^	g SO_2_-Eq
Terrestrial Ecotoxicity (TETP100)	4.40 × 10^−2^	4.10 × 10^−2^	3.80 × 10^−2^	3.50 × 10^−2^	g 1,4-DCB-Eq
Urban Land Occupation (ULOP)	1.53 × 10^−2^	1.50 × 10^−2^	1.40 × 10^−2^	1.40 × 10^−2^	m^2^a
Water Depletion (WDP)	0.467	0.432	0.418	0.372	g H_2_O (m^3^)

Note: *(±**10% sensitivity ranges).**

Likewise, other impact categories such as FDP, FETP100 and PMFP exhibited similar trends when RCP replaced cement. The FDP decreased from 236 g oil-Eq to 223.2 g oil-Eq, as RCP production does not require the high energy demand associated with clinker production. The FETP100 decreased from 27.4 g 1,4-DCB-Eq to 26.4 g 1,4-DCB-Eq, as lower toxic emissions are released during RCP production. A significant reduction in the PMFP category was recorded, decreasing from 220 g PM10-eq to 200 g PM10-eq, which is attributed to the fact that WTSF production is less energy-intensive than industrial steel fiber production. In addition, TAP20 showed a major reduction from 5110 g SO_2_-eq to 309 g SO_2_-eq, reflecting the environmental benefits of using recycled materials in the mixture.

## Conclusions

This study developed a sustainable ECC by incorporating RCP as partial cement replacement and WTSF with industrial steel fibers. The LCA analysis demonstrated that the use of RCP and WTSF resulted in considerable environmental benefits. The major findings of this study are summarized as follows:

1. ECC mixtures with 5% RCP replacement combined with 2% WTSF and 2% PE fiber achieved the highest flexural strengths of 44.5 MPa and 43.5 MPa, respectively.
2. The DIC results indicate that increasing SF and PE fiber content up to 2% improves strain distribution and promotes stable crack development under flexural loading.
3. The SEM results confirm enhanced fiber-matrix interaction and crack-bridging capacity, which contributes to improved ductility and overall flexural performance.
4. Substituting cement with RCP and using WTSF significantly reduces climate change potential (16% reduction), fossil resource use (19% reduction), toxic emissions, and water consumption, promoting more sustainable construction practices.

The DIC results present a critical balance required between RCP content and fiber reinforcement. While fibers enhance tensile performance by bridging cracks and improve strain distribution, excessive RCP content undermines matrix quality, limiting the effectiveness of fiber reinforcement. The combined DIC and SEM observations further confirmed that the interaction between matrix densification and fiber-bridging mechanisms plays a critical role in controlling crack propagation, strain distribution, and post-cracking ductility in sustainable ECC systems.

### Future research directions

Future studies should investigate the long-term durability, structural-scale behavior, and field implementation potential of RCP-based ECC mixtures under different environmental exposure conditions. Future studies should comprehensively investigate the long-term durability and service-life performance of sustainable ECC mixtures under aggressive environmental exposure conditions to validate their practical structural applicability. Further quantitative DIC analysis and advanced microstructural characterization may provide a deeper understanding of crack propagation and fiber–matrix interaction mechanisms.

### Study limitations

Despite the promising mechanical and environmental performance observed in this study, several limitations should be acknowledged. The present investigation primarily focused on flexural behavior and manufacturing-stage environmental impacts under controlled laboratory conditions. In addition, the fiber dosages in the present study were controlled based on mass proportions rather than equivalent volume fractions, which may influence crack-bridging efficiency and fiber distribution characteristics due to density differences between steel and polymer fibers.

## Supporting information

S1 File LCA. PDF
