# Supplementary material for: Sustainable engineered cementitious composites incorporating recycled materials: Experimental validation and life cycle assessment
Source: PLoS One. 2026 Aug 28;21(8):e0355961. doi: 10.1371/journal.pone.0355961 (PMC13524287; doi:10.1371/journal.pone.0355961)
Supplement: Supplementary file 1 [file pone.0355961.s001.pdf]

SUPPLEMENTARY INFORMATION

Life Cycle Assessment Data for Sustainable ECC Incorporating Recycled Concrete Powder and Waste Tyre Steel Fiber

Organized environmental inventory, nomenclature, impact categories, units, and results

This document is designed to be read independently of the main manuscript.

Table of Contents

S1. Scope and LCA reporting basis **3**

S2. Nomenclature, impact categories, and units **4**

S3. Constituent-level environmental results **5**

S4. Complete ECC mixture-level environmental results **6**

S5. Reproducibility and interpretation notes **7**

Table numbers use the prefix S to distinguish supplementary tables from those in the main manuscript.

S1. Scope and LCA reporting basis

This Supplementary Information presents the environmental results used to compare conventional and recycled constituents and complete ECC mixtures. Constituent-level comparisons use a reference flow of 1 kg of material, while mixture-level comparisons use a functional unit of 1 m3 of ECC. The cradle-to-gate boundary includes raw material extraction, processing, recycling, transportation, and manufacturing. Construction, service life, maintenance, demolition, and end-of-life stages are excluded.

Table S1. LCA framework, model settings, and reporting basis.

Reporting item	Specification
**Goal**	Compare the environmental burdens of conventional and recycled ECC constituents and mixtures
**Functional unit**	1 m3 of complete ECC mixture
**Constituent reference flow**	1 kg of cementitious or fiber material
**System boundary**	Cradle-to-gate
**Included stages**	Raw material extraction, processing, recycling, transportation, and ECC manufacturing
**Excluded stages**	Construction, operation, maintenance, demolition, and end-of-life treatment
**LCA software**	openLCA, version [insert version used]
**LCI database**	ecoinvent, version [insert version used]
**Impact assessment method**	ReCiPe 2016 Midpoint (H), version 1.03
**Number of impact categories**	18 midpoint categories
**Sensitivity reporting**	Plus or minus 10% ranges where reported in the main manuscript

Important: the openLCA and ecoinvent version numbers were not stated in the supplied source file and must be completed by the authors before submission.

S2. Nomenclature, impact categories, and units

Table S2. Mixture and constituent identifiers used in the supplementary results.

Identifier	Definition	Assessment level	Reference basis
**ISF**	Industrial steel fiber	Constituent-level comparison	1 kg
**WTSF**	Waste tyre steel fiber	Constituent-level comparison	1 kg
**C-ECC**	Conventional ECC with 0% RCP	Mixture-level baseline	1 m3
**RCP5-ECC**	Sustainable ECC containing 5% RCP	Mixture-level scenario	1 m3
**RCP10-ECC**	Sustainable ECC containing 10% RCP	Mixture-level scenario	1 m3
**RCP15-ECC**	Sustainable ECC containing 15% RCP	Mixture-level scenario	1 m3

Table S3. Impact category abbreviations and reporting units.

Code	Impact category	Unit
**ALOP**	Agricultural land occupation	m2 a
**GWP20**	Climate change potential, 20-year horizon	g CO2-eq
**FDP**	Fossil depletion potential	g oil-eq
**FETP100**	Freshwater ecotoxicity potential	g 1,4-DCB-eq
**FEP**	Freshwater eutrophication potential	g P-eq
**HTP100**	Human toxicity potential	g 1,4-DCB-eq
**IRP_I**	Ionising radiation potential	g U235-eq
**METP100**	Marine ecotoxicity potential	g 1,4-DCB-eq
**MEP**	Marine eutrophication potential	g N-eq
**MDP**	Metal depletion potential	g Fe-eq
**NLTP**	Natural land transformation potential	m2
**ODPinf**	Ozone depletion potential	g CFC-11-eq
**PMFP**	Particulate matter formation potential	g PM10-eq
**POFP**	Photochemical oxidant formation potential	g NMVOC
**TAP20**	Terrestrial acidification potential	g SO2-eq
**TETP100**	Terrestrial ecotoxicity potential	g 1,4-DCB-eq
**ULOP**	Urban land occupation	m2 a
**WDP**	Water depletion potential	m3 H2O-eq

S3. Constituent-level environmental results

Table S4 compares the production-stage environmental impacts of 1 kg of industrial steel fiber and 1 kg of waste tyre steel fiber. Lower values indicate lower environmental burden within the same impact category. The constituent-level results should not be directly compared with the complete mixture results in Section S4 because the reference bases differ.

Table S4. Environmental impact comparison of industrial steel fiber and waste tyre steel fiber per 1 kg of material.

Impact category	ISF	WTSF	Unit
**Agricultural Land Occupation (ALOP)**	0.876	0.0915	m2 a
**Climate Change (GWP20)**	1350	348	g CO2-eq
**Fossil Depletion (FDP)**	236	70.7	g oil-eq
**Freshwater Ecotoxicity (FETP100)**	27.4	9.51	g 1,4-DCB-eq
**Freshwater Eutrophication (FEP)**	1.44	0.0343	g P-eq
**Human Toxicity (HTP100)**	221	2.68	g 1,4-DCB-eq
**Ionising Radiation (IRP_I)**	19.9	9.23	g U235-eq
**Marine Ecotoxicity (METP100)**	13.2	8.59	g 1,4-DCB-eq
**Marine Eutrophication (MEP)**	1.18	0.136	g N-eq
**Metal Depletion (MDP)**	0.116	0.0549	g Fe-eq
**Natural Land Transformation (NLTP)**	5.31E-05	1.73E-05	m2
**Ozone Depletion (ODPinf)**	1.99E-05	3.27E-05	g CFC-11-eq
**Particulate Matter Formation (PMFP)**	6220	200	g PM10-eq
**Photochemical Oxidant Formation (POFP)**	3450	696	g NMVOC
**Terrestrial Acidification (TAP20)**	5110	309	g SO2-eq
**Terrestrial Ecotoxicity (TETP100)**	0.0423	1.8	g 1,4-DCB-eq
**Urban Land Occupation (ULOP)**	0.0162	0.00135	m2 a
**Water Depletion (WDP)**	0.455	1.32	m3 H2O-eq

Note: the constituent labels have been aligned with the values reported in the main manuscript, where industrial steel fiber has a climate change impact of 1350 g CO2-eq and WTSF has 348 g CO2-eq per kg.

S4. Complete ECC mixture-level environmental results

Table S5 reports the cradle-to-gate environmental impacts of the conventional ECC baseline and sustainable ECC mixtures containing 5%, 10%, and 15% RCP. All results are reported for the same functional unit of 1 m3 of ECC, enabling direct comparison across the four mixture scenarios.

Table S5. Cradle-to-gate environmental impacts of conventional and sustainable ECC mixtures per 1 m3 of ECC.

Impact category	C-ECC<br>0% RCP	RCP5-ECC<br>5% RCP	RCP10-ECC<br>10% RCP	RCP15-ECC<br>15% RCP	Unit
**Agricultural Land Occupation (ALOP)**	0.876	0.840	0.800	0.750	m2 a
**Climate Change (GWP20)**	1350	1293.52	1187.12	1135.38	g CO2-eq
**Fossil Depletion (FDP)**	236	223.16	205.61	191.76	g oil-eq
**Freshwater Ecotoxicity (FETP100)**	27.4	26.41	24.58	24.26	g 1,4-DCB-eq
**Freshwater Eutrophication (FEP)**	1.44	1.37	1.31	1.17	g P-eq
**Human Toxicity (HTP100)**	221	206.32	201.13	182.26	g 1,4-DCB-eq
**Ionising Radiation (IRP_I)**	19.9	19.43	18.15	18.22	g U235-eq
**Marine Ecotoxicity (METP100)**	13.2	12.53	12.12	11.15	g 1,4-DCB-eq
**Marine Eutrophication (MEP)**	1.18	1.14	1.03	0.96	g N-eq
**Metal Depletion (MDP)**	0.116	0.110	0.100	0.100	g Fe-eq
**Natural Land Transformation (NLTP)**	5.31E-05	5.20E-05	4.80E-05	4.60E-05	m2
**Ozone Depletion (ODPinf)**	1.99E-05	1.90E-05	1.80E-05	1.70E-05	g CFC-11-eq
**Particulate Matter Formation (PMFP)**	6220	5859.23	5515.74	5083.55	g PM10-eq
**Photochemical Oxidant Formation (POFP)**	3450	3238.46	3049.57	2857.67	g NMVOC
**Terrestrial Acidification (TAP20)**	5110	4788.87	4702.16	4132.56	g SO2-eq
**Terrestrial Ecotoxicity (TETP100)**	0.0423	0.041	0.038	0.035	g 1,4-DCB-eq
**Urban Land Occupation (ULOP)**	0.0162	0.015	0.014	0.014	m2 a
**Water Depletion (WDP)**	0.455	0.432	0.418	0.372	m3 H2O-eq

Note: lower values indicate lower burden within each impact category. Values are reproduced from the supplied final mixture-level table and presented with standardized identifiers and units.

S5. Reproducibility and interpretation notes

Reporting element	Reproducibility statement
**Data structure**	Constituent-level results and mixture-level results are reported in separate sections because they use different reference bases.
**Traceability**	Each table has a unique supplementary number, caption, identifier key, and explicit unit column.
**Functional unit**	All complete ECC mixture comparisons refer to 1 m3 of ECC.
**System boundary**	The results cover cradle-to-gate processes only and should not be interpreted as cradle-to-grave or cradle-to-cradle impacts.
**Impact interpretation**	Results should be compared within the same impact category. Values from different categories have different units and cannot be added directly.
**Rounding**	Scientific notation is used for values below 0.001. Other values are shown with sufficient precision to preserve the supplied data.
**Sensitivity**	Where uncertainty ranges are reported in the main manuscript, they represent a plus or minus 10% sensitivity analysis.
**Software and database versions**	The exact openLCA and ecoinvent version numbers must be inserted by the authors before final submission.
**Data and code availability**	All supporting data are provided in the Supplementary Information. Analysis code and supporting calculation files are available from the corresponding author upon reasonable request.

This organization replaces duplicate or ambiguous intermediate tables with a single constituent-level comparison and a single complete mixture-level comparison. The resulting file is self-contained and directly links all identifiers, units, methods, and environmental results.
