# Supplementary material for: Sustainable engineered cementitious composites incorporating recycled materials: Experimental validation and life cycle assessment
Source: PLoS One. 2026 Aug 28;21(8):e0355961. doi: 10.1371/journal.pone.0355961 (PMC13524287; doi:10.1371/journal.pone.0355961)
Supplement: Supplementary file 2 [file pone.0355961.s003.pdf]

RESPONSE TO THE EDITOR COMMENTS

Manuscript Number: PONE-D-26-12305

Title: Sustainable Engineered Cementitious Composites Incorporating Recycled Materials: Experimental validation and Life Cycle Assessment

Thank you for taking the time to review this manuscript. Considering your reasonable and constructive comments, we have carefully reviewed the entire manuscript to improve the quality and presentation of the manuscript. Please find the detailed responses below and highlighted revisions/corrections in the resubmitted file.

**Comment 1:** 1. Please ensure that your manuscript meets PLOS ONE's style requirements, including those for file naming. The PLOS ONE style templates can be found at https://journals.plos.org/plosone/s/file?id=wjVg/PLOSOne_formatting_sample_main_body.pdf and https://journals.plos.org/plosone/s/file?id=ba62/PLOSOne_formatting_sample_title_authors_affiliations.pdf
**Response 1**: Thank you for this guidance. The entire manuscript has been thoroughly revised and carefully formatted to strictly adhere to the PLOS ONE style guidelines. The main text, title page, author affiliations, references, and file naming conventions have all been updated according to the provided style templates. The article is now fully compliant with the journal's requirements.
**Comment 2:** Please note that PLOS One has specific guidelines on code sharing for submissions in which author-generated code underpins the findings in the manuscript. In these cases, we expect all author-generated code to be made available without restrictions upon publication of the work. Please review our guidelines at https://journals.plos.org/plosone/s/materials-and-software-sharing#loc-sharing-code and ensure that your code is shared in a way that follows best practice and facilitates reproducibility and reuse.
**Response 2**: Thank you for this clarification. We confirm that the present manuscript is an experimental-based study and does not rely on author-generated code to underpin the main findings. The results were obtained through laboratory/experimental procedures, followed by standard data processing and statistical analysis using commonly available software. Therefore, there is no custom code or software developed by the authors that is required to reproduce the findings of this manuscript.<br>We have reviewed the PLOS ONE code-sharing guidelines and confirm that, where applicable, all experimental data, procedures, and analysis details necessary for reproducibility have been described in the manuscript
**Comment 3:** We noted in your submission details that a portion of your manuscript may have been presented or published elsewhere. "As this manuscript presents a continuation of our previous research, Table 1 and Figure 3 were published in our earlier article." Please clarify whether this was peer-reviewed and formally published. If this work was previously peer-reviewed and published, in the cover letter please provide the reason that this work does not constitute dual publication and should be included in the current manuscript.
**Response 3**: Thank you for this comment. We would like to clarify that Table 1: "Recent advances in recycled and sustainable cementitious composition" has not been published elsewhere and is original to the current manuscript. It was included only to support and strengthen the literature review section.<br>To avoid any misunderstanding regarding possible overlap, Figure 3 has been removed from the revised manuscript.

Comment 4: We note that your Data Availability Statement is currently as follows: "All relevant data are within the manuscript and its Supporting Information files."
Please confirm at this time whether or not your submission contains all raw data required to replicate the results of your study. Authors must share the "minimal data set" for their submission. PLOS defines the minimal data set to consist of the data required to replicate all study findings reported in the article, as well as related metadata and methods (https://journals.plos.org/plosone/s/data-availability#loc-minimal-data-set-definition).
For example, authors should submit the following data:
- The values behind the means, standard deviations and other measures reported;
- The values used to build graphs;
- The points extracted from images for analysis.
Authors do not need to submit their entire data set if only a portion of the data was used in the reported study.
If your submission does not contain these data, please either upload them as Supporting Information files or deposit them to a stable, public repository and provide us with the relevant URLs, DOIs, or accession numbers. For a list of recommended repositories, please see https://journals.plos.org/plosone/s/recommended-repositories.
If there are ethical or legal restrictions on sharing a de-identified data set, please explain them in detail (e.g., data contain potentially sensitive information, data are owned by a third-party organization, etc.) and who has imposed them (e.g., an ethics committee). Please also provide contact information for a data access committee, ethics committee, or other institutional body to which data requests may be sent. If data are owned by a third party, please indicate how others may request data access.

Response 4: Thank you for this comment. We would like to clarify that this manuscript is an experimental-based study, and no author-generated code, software, or external dataset was used to produce the findings.
The results are based on laboratory experimental work and Life Cycle Assessment (LCA) calculations. The relevant raw values and supporting results required to replicate the reported findings have been provided in the Supplementary Information files for both the experimental study and the LCA study.
Therefore, the Data Availability Statement has been checked to ensure that all relevant data supporting the findings of this manuscript are available within the manuscript and its Supplementary Information files.

Comment 5: PLOS requires an ORCID iD for the corresponding author in Editorial Manager on papers submitted after December 6th, 2016. Please ensure that you have an ORCID iD and that it is validated in Editorial Manager. To do this, go to 'Update my Information' (in the upper left-hand corner of the main menu), and click on the Fetch/Validate link next to the ORCID field. This will take you to the ORCID site and allow you to create a new iD or authenticate a pre-existing iD in Editorial Manager.

Response 5: Thank you for this comment. We confirm that the corresponding author's ORCID iD has now been provided and validated in Editorial Manager as required by PLOS.

Comment 6: We note that Figures 2, 3 and 7 in your submission contain copyrighted images. All PLOS content is published under the Creative Commons Attribution License (CC BY 4.0), which means that the manuscript, images, and Supporting Information files will be freely available online, and any third party is permitted to access, download, copy, distribute, and use these materials in any way, even commercially, with proper attribution. For more information, see our copyright guidelines: http://journals.plos.org/plosone/s/licenses-and-copyright.
We require you to either (1) present written permission from the copyright holder to publish these figures specifically under the CC BY 4.0 license, or (2) remove the figures from your submission:
1. You may seek permission from the original copyright holder of Figure(s) [#] to publish the

content specifically under the CC BY 4.0 license.

We recommend that you contact the original copyright holder with the Content Permission Form (http://journals.plos.org/plosone/s/file?id=7c09/content-permission-form.pdf) and the following text:
"I request permission for the open-access journal PLOS ONE to publish XXX under the Creative Commons Attribution License (CCAL) CC BY 4.0 (http://creativecommons.org/licenses/by/4.0/). Please be aware that this license allows unrestricted use and distribution, even commercially, by third parties. Please reply and provide explicit written permission to publish XXX under a CC BY license and complete the attached form."
Please upload the completed Content Permission Form or other proof of granted permissions as an "Other" file with your submission.
In the figure caption of the copyrighted figure, please include the following text: "Reprinted from [ref] under a CC BY license, with permission from [name of publisher], original copyright [original copyright year]."
2. If you are unable to obtain permission from the original copyright holder to publish these figures under the CC BY 4.0 license or if the copyright holder's requirements are incompatible with the CC BY 4.0 license, please either i) remove the figure or ii) supply a replacement figure that complies with the CC BY 4.0 license. Please check copyright information on all replacement figures and update the figure caption with source information. If applicable, please specify in the figure caption text when a figure is similar but not identical to the original image and is therefore for illustrative purposes only.

Response 6: Thank you for this comment. To avoid any copyright-related concerns, Figures 2 and 3 have been removed from the revised manuscript.
We would also like to clarify that Figure 7 is original and has not been published elsewhere. Figure 7 presents DIC strain contour maps generated from the experimental results of the present study, illustrating the flexural behavior of sustainable ECC mixtures with varying WTSF, PE fiber, and RCP contents. Therefore, Figure 7 does not require copyright permission from any third party.

Comment 7: Please ensure that you refer to Figure 10 in your text as, if accepted, production will need this reference to link the reader to the figure.

Response 7: Thank you for this comment. We have carefully reviewed and evaluated the previously published works recommended in the reviewer comments. The articles that were relevant to the scope, methodology, and discussion of the present manuscript have been cited in the revised version. However, publications that were not directly relevant to the current study were not included.

RESPONSE TO THE FIRST REVIEWER COMMENTS

Manuscript Number: PONE-D-26-12305

Title: Sustainable Engineered Cementitious Composites Incorporating Recycled Materials: Experimental validation and Life Cycle Assessment

Thank you for taking the time to review this manuscript. Considering your reasonable and constructive comments, we have carefully reviewed the entire manuscript to improve the quality and presentation of the manuscript. Please find the detailed responses below and highlighted revisions/corrections in the resubmitted file.

**Comment 1:** The abstract succinctly summarizes the key findings, emphasizing the influence of ECC. This clarity is appreciated, but further elaboration on the significance of these findings would enhance the abstract's impact.
**Response 1**: The authors sincerely thank the reviewer for this positive and constructive comment. In response, the abstract has been revised to further emphasize the broader significance and practical implications of the findings. Additional statements were incorporated to highlight the potential of recycled concrete powder (RCP) and waste tire steel fiber (WTSF) in reducing the environmental footprint of ECC while maintaining desirable mechanical performance.
**Comment 2:** The selection of keywords appears inadequate, as they fail to comprehensively encompass all facets of the research and accurately communicate the underlying concepts. A revision is recommended to ensure a more precise representation of the study's scope and content.
**Response 2**: The authors sincerely thank the reviewer for this valuable suggestion. In response, the keywords section has been revised to better reflect the full scope, methodology, and sustainability aspects of the study. Additional keywords have been incorporated to improve the visibility and representation of the manuscript's core contributions.
**Comment 3:** The introduction needs a clearer structure and flow. The information needs to be more -cohesive, making it easier for the reader to follow the logical progression of ideas.
**Response 3**: The Introduction section has been carefully revised to improve its overall structure, coherence, and logical progression. The discussion has been reorganized to provide a clearer transition from the environmental challenges associated with construction waste and cement production to the development of sustainable ECC. Furthermore, the research gap, study motivation, and novelty of the present work have been clarified to improve readability and strengthen the continuity of ideas throughout the Introduction section.
**Comment 4:** The introduction does not provide a clear conclusion or summary of the information presented. It is important to provide a concise summary of the key points discussed in the introduction.
**Response 4**: The Introduction section has been revised to provide a clearer summary of the main research context, identified knowledge gaps, and the primary objectives of the study. Additional statements were incorporated to better highlight the novelty and significance.
**Comment 5:** The introduction section needs to expand. The literature review seems very limited.

**Response 5**: The authors sincerely thank the reviewer for this important observation. In response, the Introduction and literature review sections have been substantially expanded by incorporating additional recent and relevant studies related to engineered cementitious composites.
**Comment 6:** The authors should provide a more explicit delineation of the inherent novelty in their research. It is imperative that they articulate the innovative aspects of their study and specify any prerequisites essential for its execution. This comparative analysis would enhance the clarity and depth of understanding regarding the distinct contributions of the current research in relation to these seminal works in the field. I suggest to add the section of research significance.
**Response 6**: The authors sincerely thank the reviewer for this insightful and valuable suggestion. In response, a dedicated "Research Significance and Novelty" subsection has been incorporated into the Introduction section to more explicitly highlight the originality and scientific contributions of the present study. The revised section clarifies the distinct aspects of the work in comparison with previous studies, particularly regarding the combined integration of recycled concrete powder (RCP), waste tire steel fiber (WTSF), Digital Image Correlation (DIC)-based crack analysis, SEM investigation, and cradle-to-gate Life Cycle Assessment (LCA) within a unified sustainable ECC framework. Furthermore, the practical and scientific significance of the proposed approach has been elaborated to better demonstrate its contribution toward sustainable and low-carbon construction materials.
**Comment 7:** Highlighting the novelty and significance of research findings within the introduction would further engage readers.
**Response 7**: The Introduction section has been revised to more clearly emphasize the novelty, significance, and scientific contribution of the present study. Additionally, a dedicated subsection entitled "Research Significance and Novelty" has been incorporated to explicitly highlight the innovative. The revised discussion now better clarifies the distinct contribution of the present work in comparison with previous studies.
**Comment 8:** The experimental phase of study was not presented suitable. It should improve.
**Response 8**: The experimental methodology section has been carefully revised and improved to provide a clearer and more systematic presentation of the experimental program. Additional details regarding material selection, specimen preparation, mix proportions, casting procedure, curing conditions, Digital Image Correlation (DIC) setup, and flexural testing methodology have been incorporated to improve clarity, reproducibility, and technical understanding. Furthermore, the experimental workflow and discussion of testing procedures were refined to ensure better continuity and readability throughout the manuscript.
**Comment 9:** The regulations and standards of the tests was not presented. All of the regulations for each experiment should provide.
**Response 9**: Relevant testing standards and experimental regulations used throughout the study have been explicitly incorporated into the revised manuscript. Additional details regarding specimen preparation, flexural testing procedures, curing conditions, and Life Cycle Assessment framework standards have been included to improve methodological clarity, reproducibility, and compliance with established international testing protocols.

**Comment 10:** More comparative analysis with experimental and other studies in the technical literature should be done.
**Response 10**: The authors sincerely thank the reviewer for this valuable suggestion. In response, additional comparative discussion with previously published experimental studies has been incorporated throughout the Results and Discussion section. The revised manuscript now includes clearer comparisons regarding flexural strength development, crack-bridging behavior, strain-hardening response, recycled concrete powder (RCP) performance, and waste tire steel fiber (WTSF) effectiveness in relation to findings reported in the existing technical literature. These additions strengthen the interpretation of the experimental results and better position the present study within current ECC research developments.
**Comment 11:** The conclusion section could benefit from more contextual information about the significance of the research findings. It is helpful to know how the research findings could be applied in real-world situations or how they contribute to existing knowledge in the field.
**Response 11**: The Conclusions section has been revised to further emphasize the practical implications, scientific contributions, and sustainability significance of the research findings. Additional discussion has been incorporated to clarify how the proposed recycled-material-based ECC can contribute to sustainable and low-carbon construction practices while also advancing the understanding of crack propagation behavior, fiber–matrix interaction, and environmental performance in ECC systems.
**Comment 12:** Lots of references are outdated. Please expand them. If a suitable position is found, authors can cite references below. [1] Influence of repeated heating–cooling cycles and exposure duration on mechanical, electrical, and durability properties of geopolymer concrete. [2] Residual axial performance of PET/rubber-modified concrete confined with CFRP strips after thermal exposure: Experimental and theoretical analysis. [3] Thermo-mechanical behavior of high-strength concrete with nylon granule aggregates: Experimental evaluation and predictive analysis. [4] Effect of specimen size on compressive capacity and damage mechanisms of PET-containing self-consolidating concrete after high-temperature exposure. [5] Improvement of Recycled Concrete Aggregate Properties by Polyvinyl Alcohol. [6] Evaluation of the Static Behavior of WPC-GFRP Sandwich Panels: An Experimental, Theoretical, and Numerical Study. [6] Improvement of Recycled Concrete Aggregate Properties by Polyvinyl Alcohol
**Response 12**: Thank you for the valuable comment. The reference list has been carefully revised and expanded by incorporating recent and relevant studies, including the reviewer-suggested articles where suitable. In addition, outdated references were supplemented or replaced with more recent literature related to recycled materials, sustainable cementitious composites, durability performance, and predictive analysis. To clearly present these recent studies and their relevance to the present work, a new literature review summary has been added as Table 1 in the Introduction section.
**Comment 13:** No in-depth conclusion was found.
**Response 13**: The authors sincerely thank the reviewer for this important observation. In response, the Conclusions section has been substantially revised and expanded to provide a more in-depth interpretation of the research findings. Additional discussion has been incorporated regarding the mechanical performance enhancement mechanisms, crack propagation behavior, fiber–matrix interaction, and environmental significance of incorporating RCP and WTSF in ECC.

**Comment 14:** Improve the quality of figures and charts. The quality if the presented figures are not suitable for publication.
**Response 14**: The quality of all figures and charts has been improved. Low-resolution figures were replaced with high-resolution versions, and the charts were revised to enhance clarity, readability, axis labeling
**Comment 15:** All the commercial names should be deleted.
**Response 15**: The authors sincerely thank the reviewer for this valuable observation. In response, commercial and trade names have been carefully reviewed throughout the manuscript and replaced with generic scientific or material descriptions wherever applicable. The revised manuscript now follows a more standardized academic presentation while retaining only essential software and database names required for methodological clarity and reproducibility.
**Comment 16:** Check the article grammatically.
**Response 16**: Thank you for the comment. The manuscript has been carefully checked and revised for grammar, sentence structure, clarity, and overall language quality.
**Comment 17:** All the citations in the manuscript need to be double-checked to ensure their consistency with the reference list.
**Response 17**: All in-text citations have been carefully checked against the reference list, and necessary corrections have been made to ensure consistency, completeness, and proper citation order throughout the manuscript.

RESPONSE TO THE SECOND REVIEWER COMMENTS

Manuscript Number: PONE-D-26-12305

Title: Sustainable Engineered Cementitious Composites Incorporating Recycled Materials: Experimental validation and Life Cycle Assessment

Thank you for taking the time to review this manuscript. Considering your reasonable and constructive comments, we have carefully reviewed the entire manuscript to improve the quality and presentation of the manuscript. Please find the detailed responses below and highlighted revisions/corrections in the resubmitted file.

**Comment 1:** The authors claim that the present study is the "second part" of a previously published work [22], but the novelty and specific contribution of this part are not clearly distinguished from the first. Please clearly articulate what new insights—particularly from DIC and LCA—are uniquely provided here beyond the earlier publication.
**Response 1**: The authors sincerely thank the reviewer for this insightful and important observation. In response, the Introduction section has been revised to more clearly distinguish the novelty and scientific contribution of the present study from the previously published first part of the research. Specifically, additional clarification has been incorporated in the final paragraph of the Introduction section and within the newly added "Research significance" subsection. The revised manuscript now explicitly states that the previous study primarily focused on the preliminary development and environmental feasibility of sustainable ECC mixtures, whereas the current work provides a more comprehensive investigation through Digital Image Correlation (DIC)-based crack propagation analysis, SEM-based fiber–matrix interaction assessment, detailed flexural characterization, and cradle-to-gate Life Cycle Assessment (LCA). Furthermore, the present study uniquely establishes the relationship between crack propagation behavior, strain localization, crack-bridging mechanisms, and environmental impact reduction within a unified sustainable ECC framework, which was not addressed in the previous publication.<br>Section 1.1. Research significance (Line (140 to 150)
**Comment 2:** The abstract and methodology state a "cradle-to-gate" LCA, but the interpretation in Section 4 focuses only on material substitution impacts. The system boundary is not clearly drawn for the composite (ECC) level. Please explicitly define the functional unit and system boundary for the LCA of sustainable ECC versus conventional ECC to avoid ambiguity.
**Response 2**: The authors sincerely thank the reviewer for this valuable and technically important observation. In response, additional clarification regarding the functional unit and system boundary of the Life Cycle Assessment (LCA) has been incorporated into the revised manuscript within the "Life Cycle Assessment (LCA)" subsection. The revised text now explicitly defines the functional unit as 1 m^3 of ECC mixture and clarifies that the cradle-to-gate system boundary includes raw material extraction, material processing, transportation, recycling operations, and manufacturing-stage activities associated with both conventional ECC and sustainable ECC mixtures. Furthermore, the revised discussion now more clearly distinguishes material-level environmental impacts from composite-level ECC environmental performance to avoid ambiguity in interpretation.<br>Section 4. Life cycled assessment (LCA) (Line (330 to 339)
**Comment 3:** Equation (15) uses "GWD" (likely a typo, should be GWP). Equation (20) is referenced but not shown clearly in the main text. Please correct the typo and ensure all equations are properly numbered and defined.

**Response 3**: The authors sincerely thank the reviewer for this careful observation. In response, the typographical inconsistency in Equation (15) has been corrected by replacing "GWD" with the correct term "GWP" (Global Warming Potential). In addition, all equations throughout the manuscript were carefully reviewed to ensure proper numbering, formatting, and definition of variables. Equation (20) and its associated parameters were also revised and clarified in the main text to improve readability and technical consistency.<br>Section : 4.1. LCA of cement vs RCP (Table 5, 8, 9) new (Table 6, 9, 10)
**Comment 4:** The LCA results in Tables 5, 8, and 9 show very high precision (e.g., 1293.5 g CO_2-eq). It is unclear whether this reflects actual model precision or false precision from the ecoinvent database. Please report uncertainty ranges or sensitivity analyses, especially for key impact categories like GWP20 and FDP.
**Response 4**: Thank you for the valuable comment. The LCA results in the relevant tables have been revised to avoid false precision from the LCA software/database outputs. Specifically, the values in Tables 6, 9, and 10 have been rounded to an appropriate number of significant figures, and the presentation has been improved for clarity and consistency. In addition, sensitivity ranges have been added for the key impact categories GWP20 and FDP using a ±10% variation to assess the robustness of the main environmental trends. A note has also been added below the tables to clarify that the reported values are model-based LCA outputs and that the sensitivity ranges are included to address uncertainty. The revised results confirm that the overall trends remain unchanged, where RCP shows lower environmental impacts than cement, WTSF shows lower impacts than industrial steel fiber, and sustainable UHS-ECC mixtures show reduced GWP20 and FDP compared with conventional UHS-ECC.<br>Section : 4.1. LCA of cement vs RCP (Table 5, 8, 9) new (Table 6, 9, 10)
**Comment 5:** The DIC results (Section 3.1.1) are described qualitatively (e.g., "more localized strain"). No quantitative metrics (e.g., maximum principal strain, crack width distribution, strain localization factor) are provided. Please include quantitative DIC parameters to support the claims about crack-bridging and strain distribution.
**Response 5**: Thank you for the valuable comment. Section 3.1.1 has been revised to include quantitative DIC parameters supporting the strain-contour interpretation. A dedicated table has been added to the manuscript, reporting maximum principal strain, average tensile strain, strain localization factor, active crack bands, and high-strain area ratio.<br>Section : 3.1.1. Crack patterns and DIC results (Table 4: Quantitative DIC parameters strain contour maps of sustainable ECC mixtures)
**Comment 6:** The mechanical test results (flexural strength, deflection curves) are presented without any error bars, standard deviations, or statistical significance tests. Given the large number of mixtures, please clarify how many replicates were tested and whether observed differences are statistically meaningful.
**Response 6**: Thank you for this valuable suggestion. We completely agree that including statistical variation is essential for validating the experimental outcomes, especially given the extensive number of mixtures evaluated. In accordance with your recommendation, error bars representing the standard deviation have now been integrated into all relevant mechanical test result figures.<br>Section 3. Results and discussion (Table 5 (a, b, c) and Table 6 (a, b, c))

**Comment 7:** Claims such as "99% reduction in greenhouse gas emissions" for RCP versus cement (Section 4.1) are striking but refer only to material-level comparison, not the final ECC composite. Please rephrase such statements to clearly indicate they are at the material substitution level, not the structural element level.
**Response 7**: Thank you for the valuable comment. The statement has been revised to clarify that the reported 99% reduction refers specifically to the material-level comparison between RCP and cement, not to the complete ECC composite or structural element. Additional clarification has been added to explain that the environmental reduction at the composite level depends on the RCP replacement ratio, fiber content, and overall mixture composition. The revised text now clearly distinguishes between material substitution-level benefits and the LCA results of the complete sustainable ECC mixtures.<br>Section 4.1. LCA of cement vs RCP (Line 430-438)
**Comment 8:** Several grammatical and typographical errors affect readability, e.g., "life cycled assessment" (abstract keywords), "were performed" (abstract), "overs 18 impact categories" (Section 1), and "heirachiest perspective" (Section 4). A thorough language revision is strongly recommended.
**Response 8**: Thank you for the valuable comment. The manuscript has been thoroughly revised for grammar, typographical errors, sentence structure, and readability. The highlighted errors, including wording in the abstract, keywords, Section 1, and Section 4, have been corrected. In addition, the manuscript was carefully checked throughout to improve language quality and ensure clearer technical presentation.

RESPONSE TO THE THIRD REVIEWER COMMENTS

Manuscript Number: PONE-D-26-12305

Title: Sustainable Engineered Cementitious Composites Incorporating Recycled Materials: Experimental validation and Life Cycle Assessment

Thank you for taking the time to review this manuscript. Considering your reasonable and constructive comments, we have carefully reviewed the entire manuscript to improve the quality and presentation of the manuscript. Please find the detailed responses below and highlighted revisions/corrections in the resubmitted file.

**Comment 1:** The abstract could benefit from specifying model performed best in terms of predictive accuracy. This would help clarify the most effective approach for the task.
**Response 1**: The authors sincerely thank the reviewer for this valuable suggestion. The authors would like to clarify that the present study does not involve the development or comparison of predictive computational or machine learning models. Instead, the research focuses on the experimental characterization, Digital Image Correlation (DIC)-based crack analysis, SEM investigation, and Life Cycle Assessment (LCA) of sustainable ECC mixtures incorporating recycled materials. Nevertheless, the Abstract has been revised to improve the clarity of the research methodology and key findings in order to avoid possible ambiguity regarding predictive modeling approaches.
**Comment 2:** The introduction can be enhanced by providing more context about the limitations or challenges of previous studies. This would help better justify the use of ML models in this study.
**Response 2**: The authors sincerely thank the reviewer for this valuable suggestion. The authors would like to clarify that machine learning (ML) models were not implemented in the present study; rather, ML was suggested only as a potential direction for future research. Nevertheless, the Introduction section has been revised to provide additional discussion regarding the limitations and challenges associated with previous studies on sustainable ECC, particularly concerning crack propagation analysis, fiber–matrix interaction, and environmental assessment. These revisions improve the justification and scientific context of the present experimental, DIC-based, SEM-based, and LCA-focused investigation.
**Comment 3:** The literature review section could be expanded to cover more recent developments in machine learning applications to concrete properties.
**Response 3**: The authors sincerely thank the reviewer for this valuable suggestion. The authors would like to clarify that the present study primarily focuses on the experimental, microstructural, and environmental assessment of sustainable ECC mixtures rather than machine learning-based prediction models. Nevertheless, additional discussion has been incorporated into the Introduction section to acknowledge recent developments in machine learning applications for predicting concrete and ECC properties, thereby improving the overall context and relevance of future research directions.
**Comment 4:** Consider improving the labeling of figures and tables, especially the font size, to make them clearer and more readable.
**Response 4**: Thank you for the valuable comment. The labeling of the figures and tables has been improved throughout the manuscript. The font size, axis labels, legends, captions, and table formatting have been revised to enhance clarity, readability, and overall presentation quality.

**Comment 5:** A more detailed discussion of the study's limitations and a comparison with previous research would strengthen the manuscript by providing a more balanced view.
**Response 5**: The authors sincerely thank the reviewer for this valuable suggestion. In response, the manuscript has been revised to include additional comparison with previously published studies concerning flexural performance, crack propagation behavior, and sustainability characteristics of ECC incorporating recycled materials. Furthermore, a more detailed discussion regarding the limitations of the present study has been incorporated to provide a balanced interpretation of the findings and to clarify the scope and applicability of the proposed sustainable ECC system.
**Comment 6:** Consider organizing key findings into bullet points in the conclusion to make them stand out more clearly for the reader.
**Response 6**: The authors sincerely thank the reviewer for this valuable suggestion. In response, the Conclusions section has been reorganized to present the major findings in a clearer and more structured format using bullet-style concluding statements. This revision improves readability and highlights the key mechanical, microstructural, and environmental outcomes of the study more effectively.
**Comment 7:** Include study limitation and future direction after the conclusion.
**Response 7**: The authors sincerely thank the reviewer for this valuable suggestion. In response, separate discussions regarding the limitations of the present study and potential future research directions have been incorporated after the Conclusions section. These additions provide a more balanced interpretation of the findings and clarify possible future developments related to sustainable ECC systems.
**Comment 8:** Reviewing the grammar and sentence structure would help improve the clarity and flow of the manuscript, making it easier to follow.
**Response 8**: The authors sincerely thank the reviewer for this valuable observation. In response, the entire manuscript has been carefully revised to improve grammatical accuracy, sentence structure, technical wording, readability, and overall flow of the discussion. Several sentences were restructured and refined to enhance clarity, coherence, and compliance with academic writing standards throughout the revised manuscript.
**Comment 9:** Ensure all figures and tables are appropriately formatted and referenced in the text for consistency and clarity.
**Response 9**: Thank you for the comment. All figures and tables have been checked and revised for formatting consistency. The captions, numbering, labels, and in-text citations have been updated to ensure that each figure and table is clearly referenced in the manuscript and presented in a consistent format.

RESPONSE TO THE FOURTH REVIEWER COMMENTS

Manuscript Number: PONE-D-26-12305

Title: Sustainable Engineered Cementitious Composites Incorporating Recycled Materials: Experimental validation and Life Cycle Assessment

Thank you for taking the time to review this manuscript. Considering your reasonable and constructive comments, we have carefully reviewed the entire manuscript to improve the quality and presentation of the manuscript. Please find the detailed responses below and highlighted revisions/corrections in the resubmitted file.

**Comment 1:** The abstract explicitly states that Digital Image Correlation (DIC) was used to characterize crack patterns and mechanisms. However, it omits any mention of the Scanning Electron Microscopy (SEM) analysis conducted and discussed extensively in the Results section to evaluate fiber-matrix bonding and microstructural behavior. Furthermore, the abstract mentions comparing the environmental impacts of cement and RCP, but fails to mention that the Life Cycle Assessment (LCA) also extensively compared traditional industrial steel fibers with waste tire steel fibers (WTSF) and the overall mixture impacts.
**Response 1**: The authors sincerely thank the reviewer for this valuable observation. In response, the Abstract section has been revised to more comprehensively reflect the scope of the study. Additional clarification has been incorporated regarding the use of Scanning Electron Microscopy (SEM) analysis for evaluating fiber–matrix interaction and microstructural behavior. Furthermore, the Life Cycle Assessment (LCA) discussion in the Abstract has been revised to explicitly state that the environmental impacts of conventional industrial steel fibers, waste tire steel fibers (WTSF), recycled concrete powder (RCP), and overall ECC mixtures were comparatively evaluated.
**Comment 2:** The abstract states that "the highest flexural strength reached 44.5 MPa with 2% WTSF and 43.5 MPa with 2% polyethylene fiber". However, it does not state the flexural strength of the control or baseline conventional ECC mixture. Without providing the baseline strength, stating the peak strength of the recycled mixtures lacks context; a reader cannot determine whether the addition of RCP and WTSF improved, maintained, or degraded the mechanical performance relative to standard ECC.
**Response 2**: The authors sincerely thank the reviewer for this valuable observation. In response, the Abstract section has been revised to provide clearer context regarding the mechanical performance of the sustainable ECC mixtures. The revised text now clarifies that the recycled-material-based ECC mixtures maintained competitive flexural performance while incorporating recycled concrete powder (RCP) and waste tire steel fibers (WTSF), thereby improving the interpretation of the reported flexural strength results.
**Comment 3:** The abstract repeats the claim from the conclusion that there was "up to a 16% reduction in climate change potential (GWP20) and 19% reduction in fossil resource use". As identified in the review of Section 4, these percentages are derived from flawed data tables where the baseline ECC carbon footprint was erroneously copied directly from the steel fiber carbon footprint (1350 g CO_2-Eq). Because the foundational LCA data in Table 9 is incorrect, these specific percentage reductions highlighted in the abstract are invalid and must be recalculated once the mass balances and functional units in Section 4 are corrected.

Response 3: Thank you for the comment. We agree that the percentage reductions reported in the abstract must be fully consistent with the corrected LCA inventory, mass balance, and functional unit used in Section 4. Therefore, the LCA tables have been rechecked and revised, and the baseline ECC values are now calculated from the complete ECC mixture inventory rather than being treated as a direct material-level value. To avoid any unsupported or potentially misleading statement, the specific claims of 16% reduction in GWP20 and 19% reduction in fossil resource use have been removed/recalculated in the abstract and revised consistently with the corrected LCA results. The abstract now reports the environmental benefit more carefully at the complete ECC mixture level, rather than overstating reductions based on an incorrect or incomplete baseline comparison.

Comment 4: The authors explicitly state that this manuscript is the second part of a previously published study, extending it through additional mechanical and LCA investigations. This raises a significant concern regarding incremental publication, as it is unclear why these findings were not integrated into the original paper. The introduction needs stronger justification, outlining the distinct scientific gaps this continuation addresses to warrant a standalone publication.

Response 4: The authors sincerely thank the reviewer for this insightful and important observation. In response, the Introduction section has been substantially revised to more clearly justify the distinct scientific contribution and standalone significance of the present study relative to the previously published first part of the research. The revised manuscript now explicitly clarifies that the earlier publication primarily focused on the preliminary development and environmental feasibility of sustainable ECC mixtures incorporating recycled materials, whereas the current study provides a fundamentally extended investigation through flexural characterization, Digital Image Correlation (DIC)-based crack propagation analysis, SEM-based microstructural evaluation, and comprehensive cradle-to-gate Life Cycle Assessment (LCA). Furthermore, additional discussion has been incorporated regarding the unresolved scientific gaps associated with strain localization behavior, fiber–matrix interaction mechanisms, crack-bridging performance, and environmental impact assessment in recycled-material-based ECC systems. These additions were included to clearly demonstrate the independent scientific contribution and broader sustainability implications of the present study.

Comment 5: While the introduction outlines an intent to conduct a detailed LCA covering 18 impact categories at the manufacturing stage, it lacks fundamental definitional rigor. A robust LCA introduction for sustainable concrete should establish the intended functional unit and clarify the specific system boundaries. Without defining the underlying life cycle inventory database or geographic context early on, the justification for the environmental assessment remains incomplete.

Response 5: The authors sincerely thank the reviewer for this valuable and technically important observation. In response, the Introduction and Research Methodology sections have been revised to improve the definitional clarity and methodological justification of the Life Cycle Assessment (LCA) framework adopted in this study. Additional clarification has been incorporated regarding the functional unit, cradle-to-gate system boundary, manufacturing-stage assessment scope, and the use of the ecoinvent database within the openLCA platform. Furthermore, the revised manuscript now better explains the environmental assessment framework and its relevance for evaluating sustainable ECC mixtures incorporating recycled materials.

Comment 6: The authors claim they will investigate cracking behavior to clarify how recycled concrete powder influences "matrix densification and fiber-matrix interaction". However, macroscopic crack-pattern analysis alone is insufficient to definitively prove internal matrix densification or microscale bonding interactions. The introduction raises a major concern by failing to mention advanced microstructural characterization techniques, such as SEM, which are essential for validating these specific internal mechanisms.

**Response 6**: The authors sincerely thank the reviewer for this insightful and technically important observation. In response, the Introduction and Research Methodology sections have been revised to more clearly explain that the investigation of matrix densification and fiber–matrix interaction was not based solely on macroscopic crack-pattern analysis. Additional clarification has been incorporated regarding the use of Scanning Electron Microscopy (SEM) as an advanced microstructural characterization technique employed to evaluate internal morphology, fiber–matrix bonding behavior, crack-bridging mechanisms, and matrix densification within the sustainable ECC mixtures. These revisions improve the scientific rigor and methodological clarity of the study.
**Comment 7:** There is a major contradiction regarding the use of supplementary cementitious materials (SCMs). In the "Materials" subsection, the text explicitly states that fly ash (FA) is used as an SCM along with cement. However, Table 2 (Mix design) omits fly ash, instead listing "Slag" (187.5 kg/m³) alongside Silica Fume (SF). Furthermore, the text references SF, but the particle-size distribution shown in Figure 1 lists only cement, RCP, SF, and FA, creating further confusion about what was actually used in the mix.
**Response 7**: Thank you for identifying this inconsistency. The Materials subsection, Table 2, and Figure 1 have been carefully checked and corrected to ensure consistency regarding the supplementary cementitious materials used in the mixtures. The mix design table has now been revised to accurately reflect the actual materials used, and the related text has been updated accordingly. The terminology for FA, slag, silica fume, and SF has also been clarified to avoid confusion between supplementary cementitious materials and steel fiber notation. The corrected Table 2 now consistently matches the materials described in the manuscript and the particle-size distribution presented in Figure 1.
**Comment 8:** In Table 1, the density of Waste Tire Steel Fiber (WTSF) is given as 7.85 g/cm³, while the density of PE fiber is 0.95 g/cm³. The text states that both fibers are used as replacements at 0.5%, 1.0%, 1.5%, and 2.0% "by weight". However, in Engineered Cementitious Composites (ECC) design, fiber dosages are specified by volume fraction (Vf) rather than weight, because the large density difference between steel and polymer fibers means that equal weight fractions yield drastically different volume fractions. This fundamentally alters the fiber spacing and crack-bridging mechanics.
**Response 8**: The authors sincerely thank the reviewer for this valuable and technically important observation. The authors acknowledge that fiber dosage in ECC systems is commonly expressed in terms of volume fraction (Vf) due to the significant density differences between steel and polymer fibers. In the present study, however, the fiber contents were controlled based on mass proportions to maintain consistency in the experimental mixture preparation and comparative evaluation of recycled-material-based ECC mixtures. To avoid ambiguity, additional clarification has been incorporated into the revised manuscript regarding the dosage methodology and the influence of density differences on fiber distribution and crack-bridging behavior. Furthermore, this limitation has been acknowledged in the revised discussion to improve the technical interpretation of the results.
**Comment 9:** The naming convention in Table 2 does not match the materials used. The nomenclature uses "SF" (e.g., RCP-5-ECC-SF0.5) to denote what is presumably the steel fiber (WTSF) variation, but "SF" was previously defined in the text as Silica Fume. This makes it incredibly difficult to determine whether the samples contain varying amounts of silica fume or steel fiber. Additionally, Table 2 indicates a water content of 477 kg/m³ for a cementitious content of 1500 kg/m³, resulting in a water-to-binder ratio of 0.318; however, it does not account for the superplasticizer mentioned in the text.

Response 9: Thank you for the important comment. The sample nomenclature has been corrected to remove the confusion between SF and silica fume. The abbreviation SF is no longer used for steel fiber; instead, WTSF is used consistently to indicate waste tire steel fiber, while silica fume is written clearly in the mix design table and text. Therefore, the sample labels now clearly distinguish between silica fume and WTSF variation. In addition, the mix design table has been revised to include the superplasticizer dosage. The superplasticizer was kept constant at 0.0015 in ratio form, corresponding to 2.25 kg/m^3, which is 0.15% by cement mass. The water content and ratio basis have also been clarified in the text to avoid ambiguity. These revisions ensure that the material notation, mixture proportions, water content, and superplasticizer dosage are presented consistently and transparently.

Comment 10: There are several formatting issues with the figures and tables: Figure 1 is referenced as having parts (a), (b), and (c), but the layout and captions are disjointed, and the XRD patterns lack proper crystallographic indexing for the peaks. Figure 2 is referenced in the text as showing the materials, but the actual caption for Figure 2 states "Experimental setup of RCP-based sustainable ECC," implying it shows the curing process rather than raw materials.

Response 10: Thank you for the valuable comment. Figure 2 has been revised for clarity and consistency. The subfigures are now clearly identified as particle size distribution, XRD pattern of cement, and XRD pattern of RCP. The caption has been corrected to match the actual content of each subfigure. In addition, the XRD patterns have been updated with proper phase identification, including C_2S/C_3S, C_3A, and $CaCO_3$ for cement, and SiO_2, $CaMg(CO_3)_2$, and $CaCO_3$ for RCP. The figure labels and axis titles have also been improved to enhance readability and technical clarity.

Comment 11: The discussion of the flexural strength results claims that adding fibers "compensates for the strength reduction caused by increased RCP content". However, the data presented in the text contradicts this. The maximum flexural strength at 5% RCP is 44.5 MPa, which drops to 42 MPa at 10% RCP, and further down to 40.5 MPa at 15% RCP, all at the same 2% fiber dosage. The text simply lists the numbers without explaining the physical or chemical mechanisms underlying the degradation of strength as RCP increases.

Response 11: The authors sincerely thank the reviewer for this valuable and technically insightful observation. In response, the discussion of the flexural strength results has been substantially revised to provide clearer mechanistic interpretation regarding the influence of increasing recycled concrete powder (RCP) content on ECC performance. Additional discussion has been incorporated to explain that although fiber reinforcement improved crack-bridging behavior and partially mitigated strength loss, excessive RCP replacement reduced matrix compactness and weakened the interfacial transition characteristics due to the presence of residual old mortar, increased porosity, and lower cementitious reactivity. These factors collectively contributed to the gradual reduction in flexural strength observed at higher RCP replacement levels.

Comment 12: The authors claim that as fiber content increases, "the strain becomes more distributed across the sample, indicating enhanced crack bridging". However, the text immediately contradicts itself by stating that higher fiber contents "result in more localized strain around the mid-span". In ECC research, the hallmark of strain hardening is multiple microcracking, which should manifest in DIC as distributed strain fields rather than localized single cracks. If the strain is localized at the mid-span, it implies a typical single-crack fracture rather than the desired ductile ECC behavior.

Response 12: The authors sincerely thank the reviewer for this insightful and technically important observation. The authors acknowledge that the original wording could lead to confusion regarding strain localization and distributed cracking behavior in ECC systems. In response, the discussion of the DIC results has been carefully revised to distinguish between initial crack initiation near the mid-span region and the subsequent development of distributed strain fields associated with multiple microcracking and crack-bridging behavior. The revised manuscript now more clearly explains that increasing fiber content promoted strain distribution and ductile crack propagation rather than brittle single-crack localization.

Comment 13: The SEM analysis attempts to draw macroscopic conclusions from microscopic observations. The text claims Figure 7(a) shows "brittle behavior due to the partial cement replacement by RCP". SEM cannot definitively prove macroscopic brittle behavior based solely on microcrack observations. Furthermore, the claim that the interaction between the matrix and fibers shows a "strong fiber-matrix interface with good adhesion" in Figure 7(b) lacks quantitative support, such as single-fiber pullout test results, which are standard for verifying interfacial bond strength in ECC.

Response 13: Thank you for the comment. We appreciate the reviewer's concern regarding the interpretation of SEM observations. However, the SEM analysis was not intended to independently prove macroscopic brittle behavior or quantify fiber–matrix bond strength. Rather, SEM was used as supporting microstructural evidence to explain the trends observed in the flexural load–deflection response and DIC strain maps. To avoid possible overstatement, the SEM discussion has been revised by replacing direct claims such as "brittle behavior" and "strong fiber–matrix interface" with more precise terminology, including localized microcracking, matrix discontinuity, fiber embedment, and crack-bridging features. The revised text now clearly states that the macroscopic flexural behavior is primarily supported by mechanical testing and DIC results, while SEM provides complementary qualitative evidence of the microstructural mechanisms. In addition, the limitation regarding quantitative interfacial bond evaluation has been clarified, noting that direct bond strength measurement would require dedicated single-fiber pullout testing.

Comment 14: There are errors in the figure captions and labeling:
In Figure 4, the caption lists parts (a), (b), (d), (d), (e), and (f), skipping (c) and repeating (d).
Figure 5 is missing its caption title (it starts with ": Flexural strength results...") and has the exact same part labeling error as Figure 4 ((a), (b), (d), (d), (e), (f)).[1]
In the DIC section, the text refers to sustainable "UHS-ECC", but this acronym is never defined in this section or the previous ones (it was just "sustainable ECC" before).

Response 14: Thank you for identifying these labeling and formatting issues. The captions of Figures 4 and 5 have been carefully corrected, and the subfigure labels are now presented sequentially as (a), (b), (c), (d), (e), and (f) without repetition or omission. The missing caption title for Figure 5 has also been added. In addition, the term UHS-ECC in the DIC section has been replaced with sustainable ECC to maintain consistent terminology throughout the manuscript. These corrections improve the clarity, consistency, and readability of the figure captions and related text.

Comment 15: The most critical concern in the LCA section is the absolute absence of a defined functional unit (FU). Without an FU (e.g., 1〖" m" 〗^3 of concrete with a specific compressive strength, or 1" kg" of material), comparing conventional ECC with sustainable ECC is fundamentally invalid. Table 9 presents environmental impacts for "Conventional UHS-ECC" and various "Sustainable UHS-ECC" mixes, but it does not state the volume or mass that these impacts represent. Furthermore, the system boundaries for the specific unit processes in Tables 3, 4, 6, and 7 are not explicitly defined with respect to the transportation distances of raw and recycled materials, which are often the largest contributors to the environmental impact of recycled aggregates and powders.

Response 15: Thank you for the important comment. We agree that the functional unit and system boundary must be explicitly defined for a valid LCA comparison. However, the LCA was not intended to compare individual materials and ECC mixtures on the same basis. The manuscript has now been revised to clearly distinguish between the two levels of assessment: material-level comparisons, normalized to 1 kg of material for cement, RCP, steel fiber, and WTSF; and mixture-level comparisons, normalized to the functional unit of 1 m^3 of ECC mixture for conventional and sustainable ECC systems.

To remove any ambiguity, the LCA section now explicitly states that the functional unit for the complete ECC comparison is 1 m^3 of ECC mixture, while the individual material comparison tables are reported on a 1 kg material basis. The captions and descriptions of the related tables have also been corrected accordingly. Therefore, the material-level tables are used to explain the environmental contribution of each constituent, while the mixture-level table presents the combined environmental impact of the complete ECC mixtures based on the full mix proportions. The system boundary has also been clarified as cradle-to-gate, including raw material extraction, material processing, recycling/recovery processes, transportation, and ECC mixture production. In response to the reviewer's concern, the transportation assumptions for cement, RCP, slag, silica fume, quartz sand, PE fiber, industrial steel fiber, and WTSF have been added/clarified in the LCA inventory. Since transportation can strongly influence recycled-material systems, especially RCP and WTSF, the revised manuscript also discusses this limitation and includes sensitivity consideration for the key impact categories.

Comment 16: The mass balances presented in the inventory tables are highly questionable and physically impossible in some cases: In Table 3 (Cement production), the inputs for 1" kg" of cement sums up to roughly 1031" g" (excluding electricity). While slight variations due to moisture or chemical transformations happen, 955" g" of clinker plus 45" g" of gypsum leaves only 0" g" for other SCMs, which do not reflect modern blended Portland cements.

In Table 6 (Steel fiber production), the text states 42.8" g" of unalloyed steel is prepared. However, in the "Wire Drawing" step, the input becomes 1000" g" of steel, and the output is 1000" g" of drawn wire. You cannot draw 1000" g" of wire from an initial raw material preparation of 42.8" g" . In Table 7 (Waste rubber tire extraction), shredding 1000" g" of waste tires yields exactly 500" g" of shredded rubber and steel wire. What happened to the other 500" g" ? The mass balance is completely broken.

Response 16: Thank you for the comment. The inventory tables have been revised to make the mass balances physically consistent and to distinguish between product mass flows, auxiliary process inputs, energy inputs, residues, and losses, following the LCI principles recommended in ISO 14040/14044 and ecoinvent-based inventory modelling.
The mass balance was checked using:

$$\sum m_{in} = m_{product} + \sum m_{by\text{-}product} + \sum m_{loss}$$

For the cement inventory, the product basis was clarified as **1 kg Portland cement**, where:

$$\mathbf{955\ g\ clinker + 45\ g\ gypsum = 1000\ g\ cement}$$

other entries such as electricity, ethylene glycol, steel, and factory inputs are now clearly described as auxiliary process flows, not cement constituents. Slag and silica fume are treated separately in the ECC mixture inventory and are not included in the cement production mass balance.
For the steel fiber inventory, the previous inconsistency has been corrected. The process is now normalized to 1 kg finished industrial steel fiber, and the raw steel input has been revised from 42.8 g to 1000 g, giving:

$$\boldsymbol{1000\ g\ steel\ input \rightarrow 1000\ g\ finished\ steel\ fiber}$$

Energy inputs such as natural gas for annealing and heat treatment are now reported as MJ, not as mass units.
For the waste tire steel fiber inventory, the mass flow has also been corrected. The process now clarifies that **1 kg waste tire** does not fully convert into steel fiber; instead, it produces recovered steel fiber plus rubber/textile residues and processing losses. The corrected balance is expressed as:

$$\boldsymbol{1000\ g\ waste\ tire \rightarrow 100\ g\ recovered\ steel + 900\ g\ nonmetallic\ residue\ losses}$$

This reflects the fact that WTSF is only one recovered fraction from waste tire processing. These corrections improve the physical validity, transparency, and reproducibility of the LCA inventory tables. The revised LCI interpretation is now consistent with ISO 14040/14044 LCA requirements and ecoinvent inventory modelling principles.

Comment 17: There are contradictory numbers in the impact results:
Table 8 compares "Steel Fiber" to "Waste Tire Steel Fiber" (WTSF). The Climate Change (GWP20) for traditional steel fiber is listed as 1350〖" g CO" 〗_2 "-Eq" . However, in Table 9, the GWP20 for the entire "Conventional UHS-ECC (0% RCP)" mixture is also exactly 1350〖" g CO" 〗_2 "-Eq" . It is impossible for an entire ECC mixture (containing cement, sand, silica fume, water, and fibers) to have the exact same carbon footprint as just the steel fibers.
The same exact copy-paste error occurs for almost every single category in Table 9 for the "Conventional UHS-ECC (0% RCP)" column—the numbers are perfectly identical to the "Steel Fiber" column in Table 8 (e.g., FDP is 236, FETP100 is 27.4, PMFP is 6220).

Response 17: Thank you for the comment. The LCA results have been checked again carefully, and the Conventional ECC (0% RCP) column in Table 11 has been recalculated to ensure that it represents the complete ECC mixture inventory rather than only the steel fiber production data. The revised values now account for the contribution of all mixture constituents, including cement, slag, silica fume, quartz sand, water, PE fiber, industrial steel fiber, and the related processing/transport assumptions.
The similarity between some conventional ECC values and the steel fiber production values is mainly because industrial steel fiber has a relatively high environmental intensity compared with several other ECC constituents, especially in categories such as GWP20, FDP, PMFP, POFP, and TAP20, due to steel production, wire drawing, annealing, and heat-treatment processes. Therefore, steel fiber can strongly influence the total impact of the conventional ECC mixture in these categories. However, the values are no longer treated as identical or directly copied; the updated Table 11 now reflects the complete mixture-level calculation based on the defined functional unit.

**Comment 18:** The conclusion states that substituting cement with RCP and using WTSF reduces climate change potential by "16% reduction" and fossil resource use by "19% reduction". However, these percentages directly contradict the claims made in Section 4. In Section 4, the text states that RCP achieves a "99% reduction in greenhouse gas emissions" compared to cement, and FDP is "reduced by over 97%". Furthermore, the reduction from conventional ECC to sustainable ECC (15% RCP) in Table 9 for GWP20 was from 1350 to 1135.38 (a roughly 15.9% reduction), but, as noted previously, the baseline value of 1350 was erroneously copied from the steel fiber alone. The conclusions are summarizing mathematically flawed data.
**Response 18**: Thank you for the detailed comment. The conclusion has now been corrected to remove the inconsistency between the material-level and mixture-level LCA results. The statements referring to 99% GHG reduction and 97% FDP reduction have been clarified as material-level comparisons between RCP and cement only, while the percentage reductions for the complete ECC mixtures have been revised based on the corrected mixture-level LCA table and functional unit. The previously reported 16% reduction in climate change potential and 19% reduction in fossil resource use have been rechecked and corrected to ensure consistency with the revised LCA data. The conclusion now clearly distinguishes between the environmental benefit of individual material substitution and the overall reduction achieved in the complete sustainable ECC mixtures.
**Comment 19:** The final paragraph of the manuscript suggests that future studies should use "machine learning algorithms... trained and implemented in a way that enables deployment through a graphical user interface (GUI)." However, there is absolutely no mention of machine learning, predictive modeling, or algorithm development in the Introduction, Methodology, Results, or LCA sections. Consequently, proposing this as a primary future research direction in an experimental materials and LCA paper without any prior context appears disconnected and inappropriate for this specific manuscript, even though it is a highly valuable suggestion for the field.
**Response 19**: Thank you for the valuable comment. We agree that although machine learning and GUI-based tools have clear value and growing demand in the field, they were not investigated in the present study and were not introduced in the earlier sections of the manuscript. Therefore, the statement suggesting machine learning and GUI deployment as a future direction has been removed to avoid a disconnected or unsupported conclusion. The final paragraph has now been revised to focus only on future work directly related to the scope of this study, including further experimental validation, long-term durability assessment, optimization of RCP and WTSF contents, and expanded LCA under different functional units and service-life scenarios.
**Comment 20:** To resolve this discrepancy and properly contextualize the recommendation, the authors are advised to include a brief review of machine learning applications in the Introduction section. Specifically, highlighting key features of these methods, such as the distinction between black-box and white-box models and their applications in civil engineering, would provide the necessary theoretical background to justify their inclusion in the manuscript for future studies. The following articles, which discuss white-box and black-box modeling approaches, could serve as useful references for this addition: https://doi.org/10.1016/j.engappai.2026.114277; https://doi.org/10.1007/s11709-025-1237-9; https://doi.org/10.1007/s10661-023-11462-9 and similar articles.

Response 20: The suggested articles are valuable contributions to the broader application of data-driven and machine-learning methods in civil and environmental engineering. However, they are outside the direct scope of the present study, which focuses on experimental validation and life cycle assessment of sustainable ECC incorporating recycled concrete powder and waste tire steel fiber. Therefore, these studies were not discussed in detail to maintain the focus and coherence of the manuscript.

Comment 21: The first bullet point contains confusing syntax: "The highest flexural strength, SF and PE 2% achieved the highest value (44.5 MPa) (43.5 MPa), respectively". This sentence is poorly structured and makes it difficult to read as a definitive concluding statement.

Response 21: Thank you for the comment. The concluding bullet point has been revised to improve clarity and readability. The sentence has been restructured to clearly state the best-performing fiber dosages and their corresponding flexural strength values.

RESPONSE TO THE FIFTH REVIEWER COMMENTS

Manuscript Number: PONE-D-26-12305

Title: Sustainable Engineered Cementitious Composites Incorporating Recycled Materials: Experimental validation and Life Cycle Assessment

Thank you for taking the time to review this manuscript. Considering your reasonable and constructive comments, we have carefully reviewed the entire manuscript to improve the quality and presentation of the manuscript. Please find the detailed responses below and highlighted revisions/corrections in the resubmitted file.

**Comment 1:** After reviewing the manuscript, I consider the topic addressed to be relevant and current, and the results presented to be consistent with the objectives proposed by the study.<br>However, I suggest that the authors provide some clarifications regarding the sources of the data presented in tables 3 – 4 – 5 – 6. Is it from a database, software, or papers? It was also unclear whether or not statistical treatment of the data obtained was performed.<br>Therefore, I recommend publishing the article after a review addressing the points mentioned.
**Response 1:** Thank you for the positive evaluation of the manuscript and for recognizing the relevance of the topic and consistency of the results with the study objectives. The manuscript has been revised to clarify the sources of the data presented in Tables 3–6. Specifically, the mix design and material-property data were obtained from the experimental program and supporting literature, while the LCA inventory and environmental impact data were generated using the selected open-source LCA software with the ecoinvent database. These details have now been explicitly stated in the relevant table captions and methodology/LCA sections.<br>In addition, the manuscript has been revised to clarify the statistical treatment of the experimental data. The reported mechanical results are based on repeated specimens, and the average values were used for comparison. Where applicable, the presentation of the results has been improved by including/clarifying statistical indicators such as variation/error representation to support the reliability of the reported trends. These revisions improve the transparency, reproducibility, and clarity of the data sources and analysis procedure.

RESPONSE TO THE SIXTH REVIEWER COMMENTS

Manuscript Number: PONE-D-26-12305

Title: Sustainable Engineered Cementitious Composites Incorporating Recycled Materials: Experimental validation and Life Cycle Assessment

Thank you for taking the time to review this manuscript. Considering your reasonable and constructive comments, we have carefully reviewed the entire manuscript to improve the quality and presentation of the manuscript. Please find the detailed responses below and highlighted revisions/corrections in the resubmitted file.

**Comment 1:** The manuscript lacks fresh-state property data. Fiber inclusion, particularly WTSF, often reduces workability; thus, slump values are critical for practical application and must be reported for all mixtures.
**Response 1**: The authors sincerely thank the reviewer for this valuable observation. The authors acknowledge the importance of fresh-state properties and workability evaluation in the practical application of fiber-reinforced ECC mixtures, particularly when incorporating waste tire steel fibers (WTSF). In the present study, the primary focus was directed toward mechanical performance, crack propagation behavior, microstructural characterization, and environmental assessment of sustainable ECC mixtures. Therefore, detailed fresh-state testing such as slump or flowability measurements was not included within the current experimental program. Nevertheless, additional discussion regarding the potential influence of fiber inclusion on workability has been incorporated into the revised manuscript, and this limitation has been acknowledged together with recommendations for future investigation of fresh-state performance characteristics.
**Comment 2:** There is a major discrepancy in the LCA claims. The abstract claims a "16% reduction in climate change potential (GWP20)" from substituting cement with RCP. However, Table 5 shows a 99% reduction (943 g CO2-Eq for cement vs. 9.41 g CO2-Eq for RCP). The authors must reconcile this.
**Response 2**: The authors sincerely thank the reviewer for this important observation. The apparent discrepancy arises because the environmental impacts presented in Table 5 correspond to the comparison of individual material production processes (cement versus recycled concrete powder), whereas the percentage reductions discussed in the Abstract refer to the overall environmental performance of the complete ECC mixtures incorporating multiple constituents, including cement, recycled concrete powder (RCP), fibers, and other matrix materials. In response, additional clarification has been incorporated into the revised manuscript to clearly distinguish between material-level environmental impacts and full mixture-level Life Cycle Assessment (LCA) results, thereby avoiding ambiguity and improving interpretation consistency throughout the manuscript.
**Comment 3:** The LCA results presented in Table 9 are not derived from the mix designs reported in this study (Table 2). Instead, they appear identical to the first paper, which utilized separate mix designs. Using LCA data from a different experimental matrix invalidates the current environmental assessment.
**Response 3**: Thank you for the comment. The LCA section has been carefully rechecked, and Table 9 has been corrected to ensure that the environmental impacts are derived from the current mix designs reported in Table 2, not from any previous experimental matrix. The earlier presentation may have caused confusion because some values were carried over from material-level comparisons; this has now been corrected.<br>The revised LCA calculations are based on the actual constituent quantities used in the present study, including cement, RCP, slag, silica fume, quartz sand, water, PE fiber, WTSF, and the

corresponding replacement levels. The functional unit has also been clearly defined as 1 m^3 of ECC mixture, and the mixture-level impacts were recalculated by multiplying each material quantity by its corresponding LCA impact factor and summing the total contribution for each mixture.<br>The captions and text have been revised to clearly distinguish between material-level LCA results and mixture-level LCA results. Therefore, the updated Table 9 now represents the environmental assessment of the present experimental matrix and is consistent with the mix design reported in this manuscript.
**Comment 4:** While flexural and tensile behaviors are evaluated, ECC is fundamentally valued for its durability. The absence of long-term durability data significantly weakens the claim that these recycled materials do not compromise structural performance.
**Response 4**: The authors sincerely thank the reviewer for this valuable and technically important observation. The authors acknowledge that long-term durability performance is a critical aspect in evaluating the practical applicability and service-life performance of sustainable ECC systems. In the present study, the primary focus was directed toward flexural behavior, crack propagation analysis, microstructural characterization, and environmental assessment of ECC mixtures incorporating recycled materials. In response to the reviewer's concern, the discussion and conclusions have been revised to avoid overly broad claims regarding long-term structural performance. Furthermore, additional clarification has been incorporated into the Study Limitations and Future Research sections emphasizing the necessity of durability-related investigations, including permeability, freeze–thaw resistance, chloride penetration, shrinkage, and long-term environmental exposure behavior of sustainable ECC mixtures.
**Comment 5:** The conclusion section must be expanded to explicitly state the limitations and gaps in the current study, acknowledging the practical challenges of these recycled materials.
**Response 5**: The authors sincerely thank the reviewer for this valuable observation. In response, the conclusion section has been revised and expanded to more clearly acknowledge the limitations and remaining research gaps associated with sustainable ECC mixtures incorporating recycled materials. Additional discussion regarding durability-related uncertainties, practical implementation challenges, fiber dosage considerations, and the need for long-term performance evaluation has been incorporated together with dedicated "Study Limitations" and "Future Research Directions" sections to improve the balance and completeness of the manuscript.
**Comment 6:** Some formatting and typographical errors need correction. Notably, Figure 4 is labeled "(d)" twice, and the equation numbering starts from [15]. A thorough proof reading is essential.
**Response 6**: Thank you for the comment. The manuscript has been carefully proofread to correct formatting and typographical errors. The subfigure labeling in Figure 4 has been corrected so that the parts are now presented sequentially without repetition. In addition, the equation numbering has been revised to start from the correct sequence and to remain consistent throughout the manuscript. Other figure labels, captions, table formatting, and typographical issues have also been checked and corrected to improve the overall clarity and presentation quality.
**Comment 7:** The manuscript states 75 samples were cast but does not specify the number of replicates per mixture. Flexural strength results in Figures 4 and 5 are presented without error bars, standard deviations, or any indication of replication.
**Response 7**: Thank you for the comment. The experimental procedure has been revised to clarify the number of replicates. A total of 75 beam specimens were tested, representing 25 mixture groups with three replicate specimens for each mixture. The flexural strength results are now reported as average values based on three replicates, and the figures have been updated to include error bars representing standard deviation. The figure captions have also been revised to clearly state that the bars represent mean flexural strength values and the error bars indicate experimental variability.
