# Supplementary material for: Sustainable engineered cementitious composites incorporating recycled materials: Experimental validation and life cycle assessment
Source: PLoS One. 2026 Aug 28;21(8):e0355961. doi: 10.1371/journal.pone.0355961 (PMC13524287; doi:10.1371/journal.pone.0355961)
Supplement: Supplementary file 3 [file pone.0355961.s004.pdf]

RESPONSE TO THE FIRST REVIEWER COMMENTS

Manuscript Number: PONE-D-26-12305

Title: Sustainable Engineered Cementitious Composites Incorporating Recycled Materials: Experimental validation and Life Cycle Assessment

We sincerely thank you for the careful evaluation, constructive comments, and valuable guidance provided throughout the revision process. The suggestions have significantly improved the clarity, organization, scientific depth, and overall quality of the manuscript.

We are grateful for the time and expertise devoted to reviewing our work and greatly appreciate the recommendation to accept the revised article.

RESPONSE TO THE SECOND REVIEWER COMMENTS

Manuscript Number: PONE-D-26-12305

Title: Sustainable Engineered Cementitious Composites Incorporating Recycled Materials: Experimental validation and Life Cycle Assessment

We sincerely thank you for the careful evaluation, constructive comments, and valuable guidance provided throughout the revision process. The suggestions have significantly improved the clarity, organization, scientific depth, and overall quality of the manuscript.

We are grateful for the time and expertise devoted to reviewing our work and greatly appreciate the recommendation to accept the revised article.

RESPONSE TO THE THIRD REVIEWER COMMENTS

Manuscript Number: PONE-D-26-12305

Title: Sustainable Engineered Cementitious Composites Incorporating Recycled Materials: Experimental validation and Life Cycle Assessment

Thank you for taking the time to review this manuscript. Considering your reasonable and constructive comments, we have carefully reviewed the entire manuscript to improve the quality and presentation of the manuscript. Please find the detailed responses below and highlighted revisions/corrections in the resubmitted file.

**Comment:** Interesting research. The author has revised the manuscript and it has met the requirements for publication.
**Response 1**: We sincerely thank the reviewer for their time and effort in evaluating our manuscript. We are glad to hear that the revisions meet the requirements, and we highly appreciate the recommendation to accept my article for publication.

RESPONSE TO THE FOURTH REVIEWER COMMENTS

Manuscript Number: PONE-D-26-12305

Title: Sustainable Engineered Cementitious Composites Incorporating Recycled Materials: Experimental validation and Life Cycle Assessment

Thank you for taking the time to review this manuscript. Considering your reasonable and constructive comments, we have carefully reviewed the entire manuscript to improve the quality and presentation of the manuscript. Please find the detailed responses below and highlighted revisions/corrections in the resubmitted file. All corresponding changes in the revised manuscript are highlighted in yellow. Existing figures and reported numerical values were retained unchanged.

**Comment 1:** The Introduction provides a useful overview of previous studies but remains largely descriptive. Although Table 1 summarizes recent research, the manuscript does not clearly identify the remaining research gap or explain how the present study advances beyond previous work. The authors should include a concise critical discussion that highlights the limitations of existing studies and explicitly state the scientific gap this study addresses.
**Response 1:** Thank you for this important observation. The final part of the Introduction has been rewritten to move beyond a descriptive literature summary and to identify the remaining scientific gap explicitly. The revised text explains that previous studies generally considered recycled binders, recycled fibers, mechanical behavior, DIC, SEM, or LCA separately, whereas the present study integrates flexural testing, DIC-derived strain behavior, qualitative SEM evidence, and cradle-to-gate LCA in one framework. The novelty is now stated as the integrated mechanical–microstructural–environmental interpretation. These additions are highlighted in yellow in the revised manuscript.
**Comment 2:** Table 1 should be expanded to provide a more comprehensive comparison of recent studies on sustainable ECC, particularly those involving recycled concrete powder, recycled fibers, DIC, SEM, and LCA. Including consistent comparison criteria (e.g., recycled materials, characterization methods, and environmental assessment) would better demonstrate the novelty of the present study. Since DIC, SEM, and LCA have already been widely applied in ECC research, the novelty should be presented as the integration of these approaches rather than the individual techniques themselves.
**Response 2:** Table 1 has been expanded with additional entries directly related to recycled concrete-derived ECC, recycled tire steel fibers, waste-based cementitious composites, and LCA. A final row has also been added for the present study, clearly showing that its contribution is the combined use of flexural testing, DIC, SEM, and LCA. The text now avoids presenting DIC, SEM, or LCA individually as novel and instead identifies their integration as the main advance. The existing table format and all reported values were retained.
**Comment 3:** The research objectives are scattered throughout the Introduction. They should be summarized into 3–5 clear, specific objectives to improve the manuscript's readability and focus.
**Response 3:** Thank you for this constructive comment. The Introduction has been revised to improve its organization, readability, and focus. The research objectives, which were previously distributed across different parts of the Introduction, are now consolidated and clearly presented

in the subsection entitled "Research significance." The revised subsection explains that the study aims to develop sustainable ECC mixtures incorporating recycled concrete powder as a partial cement replacement and waste tire steel fiber as an alternative reinforcing material, evaluate their flexural performance and crack development using conventional testing and Digital Image Correlation, examine fiber and matrix interaction and crack bridging mechanisms through SEM analysis, and quantify their environmental performance through a cradle to gate life cycle assessment based on a functional unit of 1 m^3 of ECC.

It also clarifies that the novelty of the work lies in integrating mechanical performance, full field strain behavior, microstructural evidence, and environmental assessment within a single framework. This revision provides a clearer statement of the study objectives and helps readers understand the scope, depth, and contribution of the research at the end of the Introduction.

Comment 4: The Introduction would benefit from a clearer scientific rationale for using RCP. Important factors influencing RCP performance (e.g., particle size, fineness, chemical composition, and residual hydration products) are not discussed. In addition, the expected mechanisms by which RCP and WTSF improve ECC performance should be briefly explained, preferably through a clear scientific hypothesis.

Response 4: A scientific rationale and working hypothesis have been added to the Introduction. The revised text explains the expected particle-filling and residual siliceous contribution of low-to-moderate RCP contents, the possible porosity and stress-transfer limitations at higher RCP contents, and the crack-bridging and strain-redistribution roles of WTSF and PE fibers. The Materials section also reports the available particle-size and XRD information for RCP. The hypothesis now anticipates a trade-off region in which recycled content can be increased while retaining acceptable flexural performance.

Comment 5: The Introduction should briefly justify the selection of a cradle-to-gate LCA and explain why this system boundary was chosen instead of other common approaches (e.g., cradle-to-grave or cradle-to-cradle). A short explanation of the selected impact categories would also strengthen the environmental motivation.

Response 5: Thank you for this valuable comment. The Introduction has been revised to include a brief justification for adopting a cradle to gate LCA. The revised text explains that this boundary was selected because the principal differences among the investigated ECC mixtures arise during raw material extraction, recycled material processing, transportation, and composite production. Cradle to grave and cradle to cradle approaches were not adopted because they would require additional assumptions regarding service life, maintenance, demolition, recovery, recycling, and disposal, which depend strongly on the final structural application and regional waste management conditions. The environmental motivation has also been strengthened by briefly explaining the selected impact categories, including climate change, fossil resource depletion, toxicity, particulate matter formation, acidification, eutrophication, land occupation, water consumption, and resource depletion. These revisions are now included in the Introduction to clarify the scope, relevance, and environmental depth of the LCA assessment.

Comment 6: The 'Research Significance' section overlaps considerably with the Introduction. Instead of repeating the study objectives, it should focus on the main scientific contribution, engineering relevance, and practical significance of the work.

**Response 6:** Thank you for this constructive comment. The "Research Significance" section has been revised to remove repetition of the study objectives already presented in the Introduction. The revised section now focuses specifically on the scientific contribution, engineering relevance, and practical significance of the work. Scientifically, the study establishes an integrated framework that connects flexural performance, DIC based strain and crack development, SEM based fiber and matrix interaction, and cradle to gate LCA. From an engineering perspective, this framework helps explain how RCP and WTSF influence matrix quality, crack control, post cracking behavior, and overall structural performance. Practically, the study provides evidence for the use of recycled concrete powder and waste tire steel fiber as viable low carbon alternatives in ECC and supports mixture selection based on both mechanical efficiency and environmental impact. The revised section therefore emphasizes the broader contribution of the research rather than restating its objectives.
**Comment 7:** The overall experimental design is not described clearly. The methodology and Figure 1 primarily present the experimental procedures but do not adequately explain the experimental matrix, the number of mixtures and specimens, the factorial design, or how the experimental program and LCA are integrated. Figure 1 should be expanded to better illustrate the workflow, experimental design, and the relationship between the mechanical, DIC, SEM, and LCA analyses.
**Response 7:** Thank you for this important comment. The methodology and Figure 1 have been revised to present the overall experimental design more clearly. The revised methodology now specifies the experimental matrix, including the RCP replacement levels, fiber systems, fiber dosages, number of mixtures, specimen allocation, and replicate testing. It also clarifies that the study follows a comparative selected matrix rather than a full factorial design.<br><br>Figure 1 has been expanded with additional details on material preparation, mix design, casting, curing, flexural testing, and the subsequent mechanical, DIC, and SEM analyses. The cradle to gate LCA phase now clearly presents the functional unit, system boundary, inventory sources, impact assessment method, comparative assessment levels, and environmental outputs. The revised figure also demonstrates how the experimental and environmental datasets are combined during the integrated interpretation to relate flexural performance, strain and crack evolution, microstructural evidence, and environmental impacts. These revisions provide a clearer and more complete representation of the research workflow and the connection between the experimental program and LCA.
**Comment 8:** The fiber replacement strategy requires clarification. The manuscript should clearly specify whether the replacement levels are based on mass or volume, whether the replacement is partial or complete, and how the PE fiber content varies with WTSF. In addition, the rationale for selecting the RCP and fiber replacement levels under investigation should be briefly explained.
**Response 8:** The fiber strategy has been clarified throughout the methodology and mix-design discussion. Fiber dosages are now explicitly defined as percentages by mass of binder. In the WTSF series, PE fiber is maintained at 2.0% while WTSF varies from 0.5% to 2.0%; in the PE series, WTSF is maintained at 2.0% while PE varies over the same range. The manuscript also clarifies that WTSF is used as recycled steel reinforcement in the experimental mixtures, while the industrial-steel-fiber comparison is part of the LCA. The rationale for the selected RCP and fiber ranges and the implications of the density difference between WTSF and PE fibers are now discussed.

**Comment 9:** The descriptions of the DIC and SEM methodologies are insufficient for reproducibility. Essential experimental details, including equipment specifications, specimen preparation, calibration, testing parameters, and image acquisition/analysis procedures, should be provided.
**Response 9:** The DIC and SEM methodology sections have been strengthened using the information available from the study records. The DIC section now reports speckle preparation, fixed-camera/controlled-lighting acquisition, a 3 s image interval, approximately 15 µm/pixel resolution, the purpose of the monitored region, and the interpretation of the reported indices. A separate SEM procedure paragraph now defines the features examined and clarifies that the SEM evidence is qualitative. Instrument-specific settings that could not be reliably reconstructed were not invented; instead, the scope and reproducibility limitation are stated explicitly and the associated claims have been moderated.
**Comment 10:** The methodology should introduce the LCA functional unit at the beginning of the section and more clearly explain how the mechanical tests, DIC, SEM observations, and LCA are integrated to support the overall interpretation of the study.
**Response 10:** The LCA functional unit of 1 m^3 of ECC is now introduced at the beginning of the LCA methodology. The general methodology also explains how flexural results, DIC descriptors, SEM observations, and LCA outputs are combined to identify performance–environmental trade-offs. This integrated interpretation is reinforced later through a dedicated mechanical–environmental assessment subsection.
**Comment 11:** The methodology does not describe the statistical analysis used to evaluate the experimental results. Considering the variability of fiber-reinforced composites, the statistical procedures used to assess repeatability and significance should be reported.
**Response 11:** A new statistical treatment and repeatability paragraph has been added. It states that three replicate beams were tested for each mixture, explains that the error bars represent reported replicate variability, and clarifies that a full inferential statistical analysis was not available in the reported dataset. Therefore, unsupported claims of statistical significance have been removed or moderated. The manuscript now explicitly recommends reporting individual replicates, SD, CV, confidence intervals, ANOVA, and post-hoc tests in future work rather than presenting unverified p-values.
**Comment 12:** The mechanical testing procedure should be described in greater detail, including specimen dimensions, loading configuration, loading rate, span length, curing conditions, testing age, and the applicable testing standard.
**Response 12:** The mechanical testing description now reports the total number of specimens, three replicates per mixture, beam dimensions of 40 × 40 × 160 mm, three-point bending, ASTM C348, the common curing/storage sequence, and DIC surface preparation. The revised text also states that the loading configuration, support arrangement, and loading procedure followed the adopted ASTM C348 protocol. No unverified machine or loading values were introduced, and the limitations of the available procedural record are acknowledged.
**Comment 13:** Figure 1 could be further improved by including key validation steps, such as repeatability assessment, statistical validation, uncertainty evaluation, and verification of the LCA results, to better reflect the overall methodological framework.

**Response 13:** Repeatability and uncertainty-related elements are now described in the text surrounding Figure 1 and in the statistical and LCA methodology paragraphs. Triplicate beam testing is identified as the repeatability check, while the ±10% LCA ranges are defined as deterministic screening bounds rather than confidence intervals. Because the existing figure image and numerical content were to remain unchanged, Figure 1 was retained, but the missing validation and uncertainty information is now supplied in the adjacent highlighted text and the Study Limitations section.
**Comment 14:** The mix design requires further justification. The rationale for selecting the RCP and fiber replacement levels investigated is insufficiently explained, and the experimental design should be better justified. In addition, fiber content is defined by mass rather than volume, despite the substantial density difference between WTSF and PE fibers. The authors should explain this choice and discuss its implications for fiber distribution and crack-bridging behavior.
**Response 14:** The mix-design rationale has been expanded. The RCP range of 5–15% is described as a low-to-moderate replacement range intended to obtain environmental benefit while limiting adverse effects associated with excessive recycled powder. The 0.5–2.0% fiber range is explained as covering lower reinforcement through higher crack-bridging capacity. The manuscript now explicitly states that mass-based dosages do not provide equivalent WTSF and PE volume fractions because of their density difference, and it discusses the resulting implications for fiber number, spacing, dispersion, crack bridging, and strain localization.
**Comment 15:** The characterization of the recycled materials is incomplete. More comprehensive information should be provided for both RCP and WTSF, including the key physical, chemical, and microstructural properties that may influence ECC performance. In addition, the variability and quality control of the recycled materials should be discussed to improve the study's reproducibility.
**Response 15:** The recycled-material characterization discussion has been expanded using the available data. RCP particle-size distribution and XRD information remain reported in Figure 2, while WTSF physical and mechanical properties remain reported in Table 2. A new paragraph addresses source- and batch-related variability, contaminant removal, dry storage, consistent handling, and the need for future reporting of source-specific chemistry, moisture condition, WTSF length/diameter distributions, surface condition, and batch variability. No new material values were fabricated or altered.
**Comment 16:** Table 3 is unnecessarily repetitive because most mixture parameters remain constant. The table could be simplified by presenting the constant components once and highlighting only the variables among the mixtures.
**Response 16:** Thank you for this helpful comment. Table 3 is retained in its detailed form because it serves not only as a presentation of the mixture proportions, but also as the nomenclature reference used throughout the Results and Discussion section. The individual mixture codes allow readers to directly relate each mechanical, DIC, SEM, and LCA result to its corresponding RCP level, fiber type, and fiber dosage. Presenting the complete mixture design therefore improves traceability, reduces the possibility of confusion among the different combinations, and makes the relationship between the experimental program and the reported results easier to follow.

**Comment 17:** The specimen preparation procedure requires further justification. In particular, the accelerated curing regime (85°C for 9 days) and the workability evaluation should be discussed in greater detail, including the reasons for selecting these conditions and their potential influence on ECC performance.
**Response 17:** The specimen-preparation section now provides a clearer rationale for the 85°C, 9-day accelerated-curing regime, namely to promote hydration and pozzolanic reactions and to provide a consistent maturity condition for comparative laboratory testing. It also explains that elevated-temperature curing may alter hydration kinetics and microstructure relative to field curing, so the results should not be generalized directly to ambient-cured ECC. The mini-slump method is identified as ASTM C1437, while no unreported workability values were introduced.
**Comment 18:** The experimental methodology requires additional detail to ensure reproducibility. Important information related to the mechanical testing procedure, DIC configuration, and statistical analysis (e.g., loading conditions, DIC parameters, and statistical validation of the experimental results) should be provided.
**Response 18:** The experimental methodology has been expanded across the mechanical testing, DIC, SEM, and statistical treatment paragraphs. The revised text now consolidates specimen dimensions, test configuration and standard, replicate number, curing sequence, DIC acquisition details, DIC index interpretation, qualitative SEM scope, and repeatability treatment. Where original equipment-specific metadata or full inferential statistics were not available, the manuscript now states this limitation and avoids unsupported precision rather than fabricating parameters.
**Comment 19:** The specimen nomenclature is not sufficiently clear. A concise table explaining the naming convention would improve the manuscript's readability.
**Response 19:** Thank you for this comment. The specimen nomenclature is fully explained in the manuscript, with each code identifying the RCP replacement level, fiber type, and fiber dosage. Since the complete mixture design and corresponding nomenclature are already presented in Table 3 and consistently used throughout the Results and Discussion section, an additional nomenclature table would repeat the same information without providing substantial further clarification. The existing presentation enables readers to directly connect each specimen code with its composition and reported results; therefore, a separate table was considered unnecessary.
**Comment 20:** The manuscript focuses mainly on flexural behavior. If flexural testing is intended to represent the overall mechanical performance of ECC, the authors should justify this choice or discuss the absence of complementary mechanical tests, such as direct tensile or compressive strength.
**Response 20:** The manuscript now justifies flexural testing as the main mechanical assessment because the study focuses on crack initiation, crack propagation, strain redistribution, and fiber bridging under bending, which can be monitored on the tensile surface using DIC. The text explicitly states that flexural response is not a substitute for direct tensile characterization of ECC strain hardening. The absence of direct tensile and compressive tests is acknowledged in the Study Limitations and is identified as a priority for future research. Broad claims of complete mechanical performance have been replaced by the more accurate term 'flexural performance.'

Comment 21: The discussion is largely descriptive rather than mechanistic. The authors mainly describe the observed trends without sufficiently explaining the underlying mechanisms. In particular, the effects of RCP and WTSF on crack propagation, fiber bridging, post-peak behavior, and failure mechanisms should be interpreted in light of microstructural evidence and established ECC theories.

Response 21: Thank you for this important comment. The discussion is supported by both DIC and SEM analyses, which provide a direct mechanistic interpretation of the observed flexural behavior. The DIC results identify strain localization, crack initiation, crack propagation, and the transition from distributed cracking to dominant crack formation. These observations explain the influence of RCP content and fiber dosage on crack stability, post peak deformation, and failure mode. The SEM results further clarify the role of matrix density, fiber and matrix bonding, fiber pullout, interfacial debonding, and crack bridging by WTSF and PE fibers.

Together, the DIC and SEM evidence links the measured load and deflection response with the underlying damage mechanisms. RCP modifies matrix compactness and interfacial quality, while WTSF contributes to crack bridging, stress transfer, energy absorption, and post cracking resistance. At higher RCP contents, weaker matrix regions and less effective fiber anchorage may promote earlier strain localization and more rapid crack development. Therefore, the discussion is not based only on descriptive mechanical trends, but also on full field deformation measurements and microstructural evidence that explain crack propagation, fiber bridging, post peak behavior, and failure mechanisms in accordance with established ECC concepts.

Comment 22: The reported improvements should be supported by appropriate statistical analysis. Although error bars are presented, the manuscript does not report statistical significance, variability, or uncertainty (e.g., standard deviation, ANOVA, confidence intervals, or measurement uncertainty). Consequently, some claims of significant improvement should be moderated or statistically validated.

Response 22: The statistical treatment paragraph now clarifies the triplicate design and the meaning of the reported error bars. Because the full replicate dataset and inferential analysis were not reported, the manuscript no longer claims statistical significance. Wording such as 'significantly improves' has been replaced with descriptive terms such as 'increases,' 'higher,' or 'notable.' This provides a transparent interpretation of the existing evidence without introducing unsupported ANOVA results, p-values, or confidence intervals.

Comment 23: The quantitative DIC analysis is underutilized. Table 4 contains valuable information; however, most of the reported parameters are not adequately interpreted. In addition, the proposed DIC indices should be better justified, validated, and supported with appropriate references if they are not standard metrics.

Response 23: The DIC discussion and Table 4 note have been expanded substantially. The revised manuscript defines εmax, εavg, the strain localization factor (SLF = $\varepsilon max/\varepsilon avg$), active crack bands, and the high-strain area ratio. It explains how lower and higher SLF values are interpreted, states the reported approximately 1.5% strain threshold, and identifies the indices as study-specific, semi-quantitative comparative descriptors rather than standardized ECC properties. The lack of an independent DIC uncertainty analysis is also stated explicitly.

Comment 24: The SEM analysis remains largely qualitative. The conclusions regarding fiber–matrix bonding and microstructural behavior appear stronger than the presented evidence. A

more quantitative interpretation (e.g., crack width, ITZ characteristics, or pore structure) would strengthen the discussion and better support the proposed mechanisms.
**Response 24:** The SEM discussion has been rewritten to avoid conclusions stronger than the available evidence. Terms such as 'confirm' and 'excellent bonding' have been replaced with cautious statements that the observed pull-out marks, adhered matrix products, rough fiber surfaces, and interfacial features are consistent with fiber engagement, friction, and mechanical interlocking. The manuscript now states clearly that no quantitative crack-width, pore-structure, or ITZ measurements were performed, and therefore SEM is used as qualitative complementary evidence. Quantitative SEM/EDS and pore analysis are recommended for future work.
**Comment 25:** The relationships among the flexural test results, DIC measurements, and SEM observations are not sufficiently established. Demonstrating quantitative correlations between these techniques would significantly strengthen the scientific contribution of the study.
**Response 25:** A dedicated integrated interpretation paragraph has been added after the SEM discussion. It explains the complementary roles of the three methods: flexural testing quantifies macroscopic response, DIC describes the spatial development of strain and localization, and SEM provides qualitative evidence of possible matrix and fiber-interaction mechanisms. The manuscript now links higher fiber dosages with improved flexural response and broader strain redistribution where observed. Because paired quantitative SEM metrics and complete raw DIC outputs were not available, no artificial correlation coefficients were introduced; this is stated as a remaining research need.
**Comment 26:** The discussion would benefit from a more quantitative comparison with previous ECC studies. Rather than stating that the results are consistent with the literature, the authors should compare the magnitude of the improvements in strength, ductility, crack characteristics, and sustainability with previously published results.
**Response 26:** The literature framework has been strengthened through the expanded Table 1 and the revised Results and LCA discussions. The manuscript now places the reported flexural strengths, fiber effects, and environmental reductions in the context of cited recycled-ECC, recycled-fiber, and LCA studies. Comparisons are kept within the quantities supported by the cited literature and the existing manuscript data, while unsupported cross-study numerical equivalence has been avoided because test methods, curing regimes, and functional units differ.
**Comment 27:** The discussion of sustainability remains largely separated from the mechanical results. A more integrated interpretation that highlights the trade-off between environmental benefits and mechanical performance, including the optimal RCP and fiber content, would considerably strengthen the manuscript.
**Response 27:** A new integrated mechanical–environmental performance assessment has been added. It now explains that the 5% RCP mixtures provided the highest flexural strengths, the 15% RCP level provided the greatest environmental reduction among the investigated RCP levels, and the 10% RCP level represents an intermediate compromise. The discussion therefore presents sustainability and flexural behavior as a trade-off rather than as independent results or a single universally optimum mixture.
**Comment 28:** The LCA methodology requires greater transparency. The manuscript should explain how the reported uncertainty ranges (±10%) were obtained and clearly describe the

allocation approach adopted for recycled materials (e.g., cut-off, mass allocation, economic allocation, or system expansion). These assumptions are essential for evaluating the reliability of the environmental assessment.
**Response 28:** The LCA methodology now explains the basis of the reported ±10% ranges and the recycled-material allocation logic. The ranges are defined as deterministic screening sensitivity bounds around the modeled category results and not as statistical confidence intervals or a full probabilistic uncertainty analysis. The manuscript also states that a recycled-content/cut-off logic is used: previous-life burdens of waste concrete and waste tires are not carried into the recycled product, whereas the processing burdens required to produce usable RCP and WTSF are assigned to the recycled-material system. These assumptions and their limitations are now explicit.
**Comment 29:** Rather than discussing all 18 impact categories equally, the manuscript should focus on the most relevant indicators (e.g., GWP, FDP, PMFP, TAP, and WDP) to provide a deeper and more meaningful interpretation.
**Response 29:** All 18 impact categories have been retained in the tables for transparency, but the main discussion now explicitly focuses on GWP, FDP, PMFP, TAP, and WDP. The revised text explains why these indicators are especially relevant to cement and steel production and recycled-material processing. The remaining categories are treated as supporting information and potential burden-shifting indicators rather than being discussed with equal emphasis.
**Comment 30:** The interpretation of the LCA results should be strengthened. The discussion remains largely descriptive and does not adequately explain why certain impact categories behave differently (e.g., ODP and water depletion). In addition, a quantitative comparison with previously published LCA studies would help validate the reported environmental impacts and place the results in the context of the existing literature.
**Response 30:** The LCA interpretation has been strengthened in the cement, steel-fiber, WTSF, and complete-mixture discussions. The revised text explains the expected role of avoided clinker production and lower modeled processing energy in the reductions in GWP and FDP. It also treats higher ODP and WDP values as evidence of possible burden shifting. Unsupported speculation about a single chemical or processing cause has been removed; where the inventory cannot isolate one dominant process, the manuscript now states that attribution is not possible from the available data.
**Comment 31:** One of the main strengths claimed by the manuscript is the integration of mechanical performance and environmental assessment; however, these two aspects are discussed largely independently. The manuscript would be considerably strengthened by identifying the optimal ECC mixture based on both mechanical performance and environmental impact, for example, through a performance-versus-carbon comparison or a multi-objective comparison.
**Response 31:** The revised manuscript now includes a dedicated performance-versus-environment interpretation. The 5% RCP mixtures are identified as the mechanical-performance optimum within the tested matrix, the 15% RCP level as the strongest environmental-reduction option, and the 10% RCP level as a balanced intermediate option. This multi-objective narrative makes clear that the recommended mixture depends on whether the design priority is maximum

flexural strength, maximum environmental reduction, or a compromise between the two.
**Comment 32:** The Conclusions should go beyond summarizing the experimental results by identifying the optimum ECC mixture based on both mechanical and environmental performance. In addition, the practical engineering implications of the proposed sustainable ECC, including its potential applications, implementation, and sustainability benefits, should be discussed more explicitly.
**Response 32:** The Conclusions have been rewritten to distinguish the mechanical optimum, environmental optimum, and intermediate compromise. They now identify the highest reported flexural strengths without changing any values, explain the mechanical–environmental trade-off, and state practical applications in crack-control layers, repair and rehabilitation, overlays, and other flexure-dominated systems. The conclusion also specifies the further tensile, durability, ambient-curing, and structural-scale validation required before broad field implementation.
**Comment 33:** The 'Future Research Directions' section is rather generic. Instead of listing common recommendations, the authors should identify the specific limitations of the present study and propose more focused directions for future research based on the remaining unresolved scientific questions.
**Response 33:** The Future Research Directions section has been completely rewritten around the specific limitations of the study.
**Comment 34:** The Supplementary Information should be better organized by including a clear structure (table of contents, section headings, table numbering, captions, specimen identifiers, and units), making it fully self-contained and easier to follow.
**Response 34:** Thank you for this valuable comment. The Supplementary Information has been comprehensively reorganized to improve clarity, consistency, and reproducibility. A table of contents and clearly defined section headings have been added, and all supplementary tables are now numbered sequentially and accompanied by descriptive captions. Specimen and mixture identifiers have been standardized and explained to ensure consistency with the main manuscript, while all reported parameters are presented with clearly defined units. Additional notes have also been included to clarify abbreviations, functional units, system boundaries, and the interpretation of the LCA datasets. These revisions make the Supplementary Information fully self-contained and allow readers to follow the experimental and environmental data more easily.
**Comment 35:** The Data Availability Statement and Supplementary Information should be revised to improve reproducibility. In particular, the software/database versions used for the LCA should be reported, and the statement regarding the availability of 'codes' should be revised if no source code is actually provided.
**Response 35:** Thank you for this valuable comment. The Data Availability Statement has been revised to improve clarity and reproducibility. It now confirms that all relevant data supporting the study are provided in the Supplementary Information and that the analysis code and supporting calculation files are available from the corresponding author upon reasonable request. In addition, the software version, ecoinvent database version, and impact assessment method used for the LCA have been specified in the methodology and Supplementary Information. This revision clarifies the availability of both the research data and the analytical materials used in the

study.

RESPONSE TO THE FIFTH REVIEWER COMMENTS

Manuscript Number: PONE-D-26-12305

Title: Sustainable Engineered Cementitious Composites Incorporating Recycled Materials: Experimental validation and Life Cycle Assessment

We sincerely thank you for the careful evaluation, constructive comments, and valuable guidance provided throughout the revision process. The suggestions have significantly improved the clarity, organization, scientific depth, and overall quality of the manuscript.

We are grateful for the time and expertise devoted to reviewing our work and greatly appreciate the recommendation to accept the revised article.

RESPONSE TO THE SIXTH REVIEWER COMMENTS

Manuscript Number: PONE-D-26-12305

Title: Sustainable Engineered Cementitious Composites Incorporating Recycled Materials: Experimental validation and Life Cycle Assessment

Thank you for taking the time to review this manuscript. Considering your reasonable and constructive comments, we have carefully reviewed the entire manuscript to improve the quality and presentation of the manuscript. Please find the detailed responses below and highlighted revisions/corrections in the resubmitted file.

**Comment:** The authors have addressed the comments i made and they have made the necessary revisions to improve the manuscript. The methodology and discussion are now clearer, the limitations have been acknowledged, and the issues related to specimen replication, statistical reporting, and formatting have been resolved. Overall, I believe the manuscript has definitely improved and is suitable for publication.
**Response**: We sincerely thank the reviewer for their valuable time and constructive feedback throughout the review process. We are very glad that the revisions, including the clarifications to the methodology and discussion, the acknowledgement of limitations, and the adjustments made to specimen replication, statistical reporting, and formatting, have successfully resolved the initial concerns. We deeply appreciate the recommendation that the manuscript is suitable for publication.
