# Supplementary material for: Sustainable engineered cementitious composites incorporating recycled materials: Experimental validation and life cycle assessment
Source: PLoS One. 2026 Aug 28;21(8):e0355961. doi: 10.1371/journal.pone.0355961 (PMC13524287; doi:10.1371/journal.pone.0355961)
Supplement: Supplementary file 4 [file pone.0355961.s002.docx]

**Dear Authors,**

Detailed comments for each section of the manuscript are presented below.

**Abstract section:**

**#1**

The abstract explicitly states that Digital Image Correlation (DIC) was used to characterize crack patterns and mechanisms. However, it omits any mention of the Scanning Electron Microscopy (SEM) analysis conducted and discussed extensively in the Results section to evaluate fiber-matrix bonding and microstructural behavior. Furthermore, the abstract mentions comparing the environmental impacts of cement and RCP, but fails to mention that the Life Cycle Assessment (LCA) also extensively compared traditional industrial steel fibers with waste tire steel fibers (WTSF) and the overall mixture impacts.

**#2**

The abstract states that "the highest flexural strength reached 44.5 MPa with 2% WTSF and 43.5 MPa with 2% polyethylene fiber". However, it does not state the flexural strength of the control or baseline conventional ECC mixture. Without providing the baseline strength, stating the peak strength of the recycled mixtures lacks context; a reader cannot determine whether the addition of RCP and WTSF improved, maintained, or degraded the mechanical performance relative to standard ECC.

**#3**

The abstract repeats the claim from the conclusion that there was "up to a 16% reduction in climate change potential (GWP20) and 19% reduction in fossil resource use". As identified in the review of Section 4, these percentages are derived from flawed data tables where the baseline ECC carbon footprint was erroneously copied directly from the steel fiber carbon footprint (1350 g CO₂-Eq). Because the foundational LCA data in Table 9 is incorrect, these specific percentage reductions highlighted in the abstract are invalid and must be recalculated once the mass balances and functional units in Section 4 are corrected.

**Section 1:**

**#1**

The authors explicitly state that this manuscript is the second part of a previously published study, extending it through additional mechanical and LCA investigations. This raises a significant concern regarding incremental publication, as it is unclear why these findings were not integrated into the original paper. The introduction needs stronger justification, outlining the distinct scientific gaps this continuation addresses to warrant a standalone publication.

**#2**

While the introduction outlines an intent to conduct a detailed LCA covering 18 impact categories at the manufacturing stage, it lacks fundamental definitional rigor. A robust LCA introduction for sustainable concrete should establish the intended functional unit and clarify the specific system boundaries. Without defining the underlying life cycle inventory database or geographic context early on, the justification for the environmental assessment remains incomplete.

**#3**

The authors claim they will investigate cracking behavior to clarify how recycled concrete powder influences "matrix densification and fiber-matrix interaction". However, macroscopic crack-pattern analysis alone is insufficient to definitively prove internal matrix densification or microscale bonding interactions. The introduction raises a major concern by failing to mention advanced microstructural characterization techniques, such as SEM, which are essential for validating these specific internal mechanisms.

**Section 2:**

**#1**

There is a major contradiction regarding the use of supplementary cementitious materials (SCMs). In the "Materials" subsection, the text explicitly states that fly ash (FA) is used as an SCM along with cement. However, Table 2 (Mix design) omits fly ash, instead listing "Slag" (187.5 kg/m³) alongside Silica Fume (SF). Furthermore, the text references SF, but the particle-size distribution shown in Figure 1 lists only cement, RCP, SF, and FA, creating further confusion about what was actually used in the mix.

**#2**

In Table 1, the density of Waste Tire Steel Fiber (WTSF) is given as 7.85 g/cm³, while the density of PE fiber is 0.95 g/cm³. The text states that both fibers are used as replacements at 0.5%, 1.0%, 1.5%, and 2.0% "by weight". However, in Engineered Cementitious Composites (ECC) design, fiber dosages are specified by volume fraction (Vf) rather than weight, because the large density difference between steel and polymer fibers means that equal weight fractions yield drastically different volume fractions. This fundamentally alters the fiber spacing and crack-bridging mechanics.

**#3**

The naming convention in Table 2 does not match the materials used. The nomenclature uses "SF" (e.g., RCP-5-ECC-SF0.5) to denote what is presumably the steel fiber (WTSF) variation, but "SF" was previously defined in the text as Silica Fume. This makes it incredibly difficult to determine whether the samples contain varying amounts of silica fume or steel fiber. Additionally, Table 2 indicates a water content of 477 kg/m³ for a cementitious content of 1500 kg/m³, resulting in a water-to-binder ratio of 0.318; however, it does not account for the superplasticizer mentioned in the text.

**#4**

There are several formatting issues with the figures and tables:

- Figure 1 is referenced as having parts (a), (b), and (c), but the layout and captions are disjointed, and the XRD patterns lack proper crystallographic indexing for the peaks.
- Figure 2 is referenced in the text as showing the materials, but the actual caption for Figure 2 states "Experimental setup of RCP-based sustainable ECC," implying it shows the curing process rather than raw materials.

**Section 3**

**#1**

The discussion of the flexural strength results claims that adding fibers "compensates for the strength reduction caused by increased RCP content". However, the data presented in the text contradicts this. The maximum flexural strength at 5% RCP is 44.5 MPa, which drops to 42 MPa at 10% RCP, and further down to 40.5 MPa at 15% RCP, all at the same 2% fiber dosage. The text simply lists the numbers without explaining the physical or chemical mechanisms underlying the degradation of strength as RCP increases.

**#2**

The authors claim that as fiber content increases, "the strain becomes more distributed across the sample, indicating enhanced crack bridging". However, the text immediately contradicts itself by stating that higher fiber contents "result in more localized strain around the mid-span". In ECC research, the hallmark of strain hardening is multiple microcracking, which should manifest in DIC as distributed strain fields rather than localized single cracks. If the strain is localized at the mid-span, it implies a typical single-crack fracture rather than the desired ductile ECC behavior.

**#3**

The SEM analysis attempts to draw macroscopic conclusions from microscopic observations. The text claims Figure 7(a) shows "brittle behavior due to the partial cement replacement by RCP". SEM cannot definitively prove macroscopic brittle behavior based solely on microcrack observations. Furthermore, the claim that the interaction between the matrix and fibers shows a "strong fiber-matrix interface with good adhesion" in Figure 7(b) lacks quantitative support, such as single-fiber pullout test results, which are standard for verifying interfacial bond strength in ECC.

**#4**

There are errors in the figure captions and labeling:

- In Figure 4, the caption lists parts (a), (b), (d), (d), (e), and (f), skipping (c) and repeating (d).
- Figure 5 is missing its caption title (it starts with ": Flexural strength results...") and has the exact same part labeling error as Figure 4 ((a), (b), (d), (d), (e), (f)).[1]
- In the DIC section, the text refers to sustainable "UHS-ECC", but this acronym is never defined in this section or the previous ones (it was just "sustainable ECC" before).

**Section 4:**

**#1**

The most critical concern in the LCA section is the absolute absence of a defined functional unit (FU). Without an FU (e.g., $1\text{ m}^{3}$ of concrete with a specific compressive strength, or $1\text{ kg}$ of material), comparing conventional ECC with sustainable ECC is fundamentally invalid. Table 9 presents environmental impacts for "Conventional UHS-ECC" and various "Sustainable UHS-ECC" mixes, but it does not state the volume or mass that these impacts represent. Furthermore, the system boundaries for the specific unit processes in Tables 3, 4, 6, and 7 are not explicitly defined with respect to the transportation distances of raw and recycled materials, which are often the largest contributors to the environmental impact of recycled aggregates and powders.

**#2**

The mass balances presented in the inventory tables are highly questionable and physically impossible in some cases:

- In Table 3 (Cement production), the inputs for $1\text{ kg}$ of cement sums up to roughly $1031\text{ g}$ (excluding electricity). While slight variations due to moisture or chemical transformations happen, $955\text{ g}$ of clinker plus $45\text{ g}$ of gypsum leaves only $0\text{ g}$ for other SCMs, which do not reflect modern blended Portland cements.
- In Table 6 (Steel fiber production), the text states $42.8\text{ g}$ of unalloyed steel is prepared. However, in the "Wire Drawing" step, the input becomes $1000\text{ g}$ of steel, and the output is $1000\text{ g}$ of drawn wire. You cannot draw $1000\text{ g}$ of wire from an initial raw material preparation of $42.8\text{ g}$.
- In Table 7 (Waste rubber tire extraction), shredding $1000\text{ g}$ of waste tires yields exactly $500\text{ g}$ of shredded rubber and steel wire. What happened to the other $500\text{ g}$? The mass balance is completely broken.

**#3**

There are contradictory numbers in the impact results:

- Table 8 compares "Steel Fiber" to "Waste Tire Steel Fiber" (WTSF). The Climate Change (GWP20) for traditional steel fiber is listed as $1350\text{ g CO}_{2}\text{-Eq}$. However, in Table 9, the GWP20 for the entire "Conventional UHS-ECC (0% RCP)" mixture is also exactly $1350\text{ g CO}_{2}\text{-Eq}$. It is impossible for an entire ECC mixture (containing cement, sand, silica fume, water, and fibers) to have the exact same carbon footprint as just the steel fibers.
- The same exact copy-paste error occurs for almost every single category in Table 9 for the "Conventional UHS-ECC (0% RCP)" column—the numbers are perfectly identical to the "Steel Fiber" column in Table 8 (e.g., FDP is 236, FETP100 is 27.4, PMFP is 6220).

**#4**

The text claims that in Table 9, the PMFP decreased from 220 to 200, but Table 9 shows the PMFP actually decreasing from 6220 to 5859.23. The text states "TAP20 showed a major reduction from 5110 to 309" for the sustainable mixture, but Table 9 shows it decreasing from 5110 to 4788.87. The textual discussion does not align with the data presented in the tables.

**Section 5**

**#1**

The conclusion states that substituting cement with RCP and using WTSF reduces climate change potential by "16% reduction" and fossil resource use by "19% reduction". However, these percentages directly contradict the claims made in Section 4. In Section 4, the text states that RCP achieves a "99% reduction in greenhouse gas emissions" compared to cement, and FDP is "reduced by over 97%". Furthermore, the reduction from conventional ECC to sustainable ECC (15% RCP) in Table 9 for GWP20 was from 1350 to 1135.38 (a roughly 15.9% reduction), but, as noted previously, the baseline value of 1350 was erroneously copied from the steel fiber alone. The conclusions are summarizing mathematically flawed data.

**#2**

The final paragraph of the manuscript suggests that future studies should use "machine learning algorithms... trained and implemented in a way that enables deployment through a graphical user interface (GUI)." However, there is absolutely no mention of machine learning, predictive modeling, or algorithm development in the Introduction, Methodology, Results, or LCA sections. Consequently, proposing this as a primary future research direction in an experimental materials and LCA paper without any prior context appears disconnected and inappropriate for this specific manuscript, even though it is a highly valuable suggestion for the field.

To resolve this discrepancy and properly contextualize the recommendation, the authors are advised to include a brief review of machine learning applications in the Introduction section. Specifically, highlighting key features of these methods, such as the distinction between black-box and white-box models and their applications in civil engineering, would provide the necessary theoretical background to justify their inclusion in the manuscript for future studies. The following articles, which discuss white-box and black-box modeling approaches, could serve as useful references for this addition: <https://doi.org/10.1016/j.engappai.2026.114277>; <https://doi.org/10.1007/s11709-025-1237-9>; <https://doi.org/10.1007/s10661-023-11462-9> and similar articles.

**#3**

The first bullet point contains confusing syntax: "The highest flexural strength, SF and PE 2% achieved the highest value (44.5 MPa) (43.5 MPa), respectively". This sentence is poorly structured and makes it difficult to read as a definitive concluding statement.
