# Supplementary material for: Sustainable engineered cementitious composites incorporating recycled materials: Experimental validation and life cycle assessment
Source: PLoS One. 2026 Aug 28;21(8):e0355961. doi: 10.1371/journal.pone.0355961 (PMC13524287; doi:10.1371/journal.pone.0355961)
Supplement: Peer review history

# Peer review: Decision Letter 0

**DOI:** 10.1371/journal.pone.0355961.r001

**Authors:** Badrinarayan Rath

17 May 2026

Dear Dr. Ahmad,

Thank you for submitting your manuscript to PLOS ONE. After careful consideration, we feel that it has merit but does not fully meet PLOS ONE’s publication criteria as it currently stands. Therefore, we invite you to submit a revised version of the manuscript that addresses the points raised during the review process.

The manuscript addresses an important topic related to sustainable engineered cementitious composites incorporating recycled concrete powder and waste tire steel fibers. The integration of experimental investigation with life cycle assessment is appreciable and the study demonstrates potential practical relevance in sustainable construction materials. However, after a detailed technical evaluation, several significant concerns were identified regarding the experimental methodology, ECC characterization, curing regime, statistical validation, and LCA framework. These issues affect the scientific rigor and reliability of the presented findings. Therefore, the following technical comments and suggestions are provided for the authors’ careful consideration and necessary revision.

Please submit your revised manuscript by Jul 01 2026 11:59PM. If you will need more time than this to complete your revisions, please reply to this message or contact the journal office at plosone@plos.org. When you're ready to submit your revision, log on to https://www.editorialmanager.com/pone/ and select the 'Submissions Needing Revision' folder to locate your manuscript file.

- A letter that responds to each point raised by the academic editor and reviewer(s). You should upload this letter as a separate file labeled 'Response to Reviewers'.
- A marked-up copy of your manuscript that highlights changes made to the original version. You should upload this as a separate file labeled 'Revised Manuscript with Track Changes'.
- An unmarked version of your revised paper without tracked changes. You should upload this as a separate file labeled 'Manuscript'.

If you would like to make changes to your financial disclosure, please include your updated statement in your cover letter. Guidelines for resubmitting your figure files are available below the reviewer comments at the end of this letter.

If applicable, we recommend that you deposit your laboratory protocols in protocols.io to enhance the reproducibility of your results. Protocols.io assigns your protocol its own identifier (DOI) so that it can be cited independently in the future. For instructions see: https://journals.plos.org/plosone/s/submission-guidelines#loc-laboratory-protocols. Additionally, PLOS ONE offers an option for publishing peer-reviewed Lab Protocol articles, which describe protocols hosted on protocols.io. Read more information on sharing protocols at https://plos.org/protocols?utm_medium=editorial-email&utm_source=authorletters&utm_campaign=protocols.

As the corresponding author, your ORCID iD is verified in the submission system and will appear in the published article. PLOS supports the use of ORCID, and we encourage all coauthors to register for an ORCID iD and use it as well. Please encourage your coauthors to verify their ORCID iD within the submission system before final acceptance, as unverified ORCID iDs will not appear in the published article. *Only*  the individual author can complete the verification step; PLOS staff *cannot*  verify ORCID iDs on behalf of authors.

We look forward to receiving your revised manuscript.

Kind regards,

Badrinarayan Rath, PhD

Academic Editor

PLOS One

Journal Requirements:

When submitting your revision, we need you to address these additional requirements.

1. Please ensure that your manuscript meets PLOS ONE's style requirements, including those for file naming. The PLOS ONE style templates can be found at

https://journals.plos.org/plosone/s/file?id=wjVg/PLOSOne_formatting_sample_main_body.pdf and

https://journals.plos.org/plosone/s/file?id=ba62/PLOSOne_formatting_sample_title_authors_affiliations.pdf

2. Please note that PLOS One has specific guidelines on code sharing for submissions in which author-generated code underpins the findings in the manuscript. In these cases, we expect all author-generated code to be made available without restrictions upon publication of the work. Please review our guidelines at https://journals.plos.org/plosone/s/materials-and-software-sharing#loc-sharing-code and ensure that your code is shared in a way that follows best practice and facilitates reproducibility and reuse.

3. We noted in your submission details that a portion of your manuscript may have been presented or published elsewhere. “As this manuscript presents a continuation of our previous research, Table 1 and Figure 3 were published in our earlier article.” Please clarify whether this was peer-reviewed and formally published. If this work was previously peer-reviewed and published, in the cover letter please provide the reason that this work does not constitute dual publication and should be included in the current manuscript.

4. We note that your Data Availability Statement is currently as follows: “All relevant data are within the manuscript and its Supporting Information files.”

Please confirm at this time whether or not your submission contains all raw data required to replicate the results of your study. Authors must share the “minimal data set” for their submission. PLOS defines the minimal data set to consist of the data required to replicate all study findings reported in the article, as well as related metadata and methods (https://journals.plos.org/plosone/s/data-availability#loc-minimal-data-set-definition).

For example, authors should submit the following data:

- The values behind the means, standard deviations and other measures reported;

- The values used to build graphs;

- The points extracted from images for analysis.

Authors do not need to submit their entire data set if only a portion of the data was used in the reported study.

If your submission does not contain these data, please either upload them as Supporting Information files or deposit them to a stable, public repository and provide us with the relevant URLs, DOIs, or accession numbers. For a list of recommended repositories, please see https://journals.plos.org/plosone/s/recommended-repositories.

If there are ethical or legal restrictions on sharing a de-identified data set, please explain them in detail (e.g., data contain potentially sensitive information, data are owned by a third-party organization, etc.) and who has imposed them (e.g., an ethics committee). Please also provide contact information for a data access committee, ethics committee, or other institutional body to which data requests may be sent. If data are owned by a third party, please indicate how others may request data access.

5. PLOS requires an ORCID iD for the corresponding author in Editorial Manager on papers submitted after December 6th, 2016. Please ensure that you have an ORCID iD and that it is validated in Editorial Manager. To do this, go to ‘Update my Information’ (in the upper left-hand corner of the main menu), and click on the Fetch/Validate link next to the ORCID field. This will take you to the ORCID site and allow you to create a new iD or authenticate a pre-existing iD in Editorial Manager.

6. We note that Figures 2, 3 and 7 in your submission contain copyrighted images. All PLOS content is published under the Creative Commons Attribution License (CC BY 4.0), which means that the manuscript, images, and Supporting Information files will be freely available online, and any third party is permitted to access, download, copy, distribute, and use these materials in any way, even commercially, with proper attribution. For more information, see our copyright guidelines: http://journals.plos.org/plosone/s/licenses-and-copyright.

We require you to either (1) present written permission from the copyright holder to publish these figures specifically under the CC BY 4.0 license, or (2) remove the figures from your submission:

1. You may seek permission from the original copyright holder of Figure(s) [#] to publish the content specifically under the CC BY 4.0 license.

We recommend that you contact the original copyright holder with the Content Permission Form (http://journals.plos.org/plosone/s/file?id=7c09/content-permission-form.pdf) and the following text:

“I request permission for the open-access journal PLOS ONE to publish XXX under the Creative Commons Attribution License (CCAL) CC BY 4.0 (http://creativecommons.org/licenses/by/4.0/). Please be aware that this license allows unrestricted use and distribution, even commercially, by third parties. Please reply and provide explicit written permission to publish XXX under a CC BY license and complete the attached form.”

Please upload the completed Content Permission Form or other proof of granted permissions as an "Other" file with your submission.

In the figure caption of the copyrighted figure, please include the following text: “Reprinted from [ref] under a CC BY license, with permission from [name of publisher], original copyright [original copyright year].”

2. If you are unable to obtain permission from the original copyright holder to publish these figures under the CC BY 4.0 license or if the copyright holder’s requirements are incompatible with the CC BY 4.0 license, please either i) remove the figure or ii) supply a replacement figure that complies with the CC BY 4.0 license. Please check copyright information on all replacement figures and update the figure caption with source information. If applicable, please specify in the figure caption text when a figure is similar but not identical to the original image and is therefore for illustrative purposes only.

7. Please ensure that you refer to Figure 10 in your text as, if accepted, production will need this reference to link the reader to the figure.

8. If the reviewer comments include a recommendation to cite specific previously published works, please review and evaluate these publications to determine whether they are relevant and should be cited. There is no requirement to cite these works unless the editor has indicated otherwise.

Additional Editor Comments :

1. The study used PE fiber = up to 2% and WTSF = up to 2%. Leading to approximately 4% total fiber volume fraction, which is extremely high for ECC and likely causes severe workability, fiber balling, and dispersion problems. ECC generally uses optimized micromechanics-based fiber content (~2% total). Excessive hybrid fiber dosage may invalidate uniform strain-hardening assumptions. The combined PE and WTSF content reaches unrealistically high fiber fractions without proper rheological or dispersion assessment. The manuscript lacks evidence regarding workability stability, fiber distribution, and avoidance of fiber agglomeration, raising concerns about practical feasibility and repeatability.

2. The manuscript claims ECC behavior; however, no uniaxial tensile test or compressive strength test has been conducted. Since ECC classification fundamentally depends on tensile strain-hardening characteristics and ductility, the presented experimental program is incomplete and technically insufficient.

3. The use of accelerated water curing at 85°C for 9 days is not representative of practical field conditions and may artificially enhance hydration and mechanical properties. The manuscript lacks justification for selecting this curing regime and does not compare results with standard curing conditions.

4. The LCA framework lacks a clearly defined functional unit and system boundary consistency. The reported environmental reductions appear overly optimistic and insufficiently justified. Important parameters such as transportation distance, energy consumption during recycling, and allocation assumptions are missing, which weakens the credibility of the sustainability assessment.

5. The manuscript lacks statistical validation of experimental data. No standard deviation, variance analysis, or significance testing is presented. Considering the inherent variability in fiber-reinforced composites, the reliability and reproducibility of the reported improvements remain uncertain.

6. The manuscript presents an interesting sustainability concept using RCP and WTSF in ECC; however, major shortcomings exist in: ECC characterization methodology, experimental validation, curing realism, statistical reliability, and LCA rigor. Substantial technical revision is required before the manuscript can be considered scientifically reliable for publication.

[Note: HTML markup is below. Please do not edit.]

Reviewers' comments:

Reviewer's Responses to Questions

**Comments to the Author**

1. Is the manuscript technically sound, and do the data support the conclusions?

Reviewer #1: Yes

Reviewer #2: Partly

Reviewer #3: Partly

Reviewer #4: Partly

Reviewer #5: Partly

Reviewer #6: Partly

**********

2. Has the statistical analysis been performed appropriately and rigorously?

Reviewer #1: Yes

Reviewer #2: Yes

Reviewer #3: I Don't Know

Reviewer #4: Yes

Reviewer #5: I Don't Know

Reviewer #6: No

**********

3. Have the authors made all data underlying the findings in their manuscript fully available?

The PLOS Data policy

Reviewer #1: Yes

Reviewer #2: Yes

Reviewer #3: No

Reviewer #4: No

Reviewer #5: No

Reviewer #6: Yes

**********

4. Is the manuscript presented in an intelligible fashion and written in standard English?

Reviewer #1: Yes

Reviewer #2: Yes

Reviewer #3: Yes

Reviewer #4: Yes

Reviewer #5: Yes

Reviewer #6: Yes

**********

Reviewer #1: The manuscript titled “Sustainable Engineered Cementitious Composites Incorporating Recycled Materials: Experimental validation and Life Cycle Assessment” addresses a pertinent research topic within the realm of structural engineering. To enhance the manuscript's clarity, it is essential to refine the articulation of key concepts, ensuring that the arguments are logically structured and effectively communicated. Additionally, the methodological approach should be strengthened by providing a more detailed explanation of the research design, data analysis techniques, and validation processes to ensure scientific rigor. Furthermore, a deeper discussion of the findings, including their broader implications and potential applications, would significantly enhance the manuscript’s impact. Addressing these aspects will improve the overall coherence, credibility, and contribution of the study, aligning it with the expected scholarly standards.

1- The abstract succinctly summarizes the key findings, emphasizing the influence of ECC. This clarity is appreciated, but further elaboration on the significance of these findings would enhance the abstract's impact.

2- The selection of keywords appears inadequate, as they fail to comprehensively encompass all facets of the research and accurately communicate the underlying concepts. A revision is recommended to ensure a more precise representation of the study’s scope and content.

3- The introduction needs a clearer structure and flow. The information needs to be more -cohesive, making it easier for the reader to follow the logical progression of ideas.

4- The introduction does not provide a clear conclusion or summary of the information presented. It is important to provide a concise summary of the key points discussed in the introduction.

5- The introduction section needs to expand. The literature review seems very limited.

6- The authors should provide a more explicit delineation of the inherent novelty in their research. It is imperative that they articulate the innovative aspects of their study and specify any prerequisites essential for its execution. This comparative analysis would enhance the clarity and depth of understanding regarding the distinct contributions of the current research in relation to these seminal works in the field. I suggest to add the section of research significance.

7- Highlighting the novelty and significance of research findings within the introduction would further engage readers.

9- The experimental phase of study was not presented suitable. It should improve.

10- The regulations and standards of the tests was not presented. All of the regulations for each experiment should provide.

11- More comparative analysis with experimental and other studies in the technical literature should be done.

12- The conclusion section could benefit from more contextual information about the significance of the research findings. It is helpful to know how the research findings could be applied in real-world situations or how they contribute to existing knowledge in the field.

13- Lots of references are outdated. Please expand them. If a suitable position is found, authors can cite references below. [1] Influence of repeated heating–cooling cycles and exposure duration on mechanical, electrical, and durability properties of geopolymer concrete. [2] Residual axial performance of PET/rubber-modified concrete confined with CFRP strips after thermal exposure: Experimental and theoretical analysis. [3] Thermo-mechanical behavior of high-strength concrete with nylon granule aggregates: Experimental evaluation and predictive analysis. [4] Effect of specimen size on compressive capacity and damage mechanisms of PET-containing self-consolidating concrete after high-temperature exposure. [5] Improvement of Recycled Concrete Aggregate Properties by Polyvinyl Alcohol. [6] Evaluation of the Static Behavior of WPC-GFRP Sandwich Panels: An Experimental, Theoretical, and Numerical Study. [6] Improvement of Recycled Concrete Aggregate Properties by Polyvinyl Alcohol

14- No in-depth conclusion was found.

15- Improve the quality of figures and charts. The quality if the presented figures are not suitable for publication.

16- All the commercial names should be deleted.

17- Check the article grammatically.

18- All the citations in the manuscript need to be double-checked to ensure their consistency with the reference list.

19- Remove the gray border of the presented curves.

Reviewer #2: 1. The authors claim that the present study is the "second part" of a previously published work [22], but the novelty and specific contribution of this part are not clearly distinguished from the first. Please clearly articulate what new insights—particularly from DIC and LCA—are uniquely provided here beyond the earlier publication.

2. The abstract and methodology state a "cradle-to-gate" LCA, but the interpretation in Section 4 focuses only on material substitution impacts. The system boundary is not clearly drawn for the composite (ECC) level. Please explicitly define the functional unit and system boundary for the LCA of sustainable ECC versus conventional ECC to avoid ambiguity.

3. Equation (15) uses "GWD" (likely a typo, should be GWP). Equation (20) is referenced but not shown clearly in the main text. Please correct the typo and ensure all equations are properly numbered and defined.

4. The LCA results in Tables 5, 8, and 9 show very high precision (e.g., 1293.5 g CO₂-eq). It is unclear whether this reflects actual model precision or false precision from the ecoinvent database. Please report uncertainty ranges or sensitivity analyses, especially for key impact categories like GWP20 and FDP.

5. The DIC results (Section 3.1.1) are described qualitatively (e.g., "more localized strain"). No quantitative metrics (e.g., maximum principal strain, crack width distribution, strain localization factor) are provided. Please include quantitative DIC parameters to support the claims about crack-bridging and strain distribution.

6. The mechanical test results (flexural strength, deflection curves) are presented without any error bars, standard deviations, or statistical significance tests. Given the large number of mixtures, please clarify how many replicates were tested and whether observed differences are statistically meaningful.

7. Claims such as "99% reduction in greenhouse gas emissions" for RCP versus cement (Section 4.1) are striking but refer only to material-level comparison, not the final ECC composite. Please rephrase such statements to clearly indicate they are at the material substitution level, not the structural element level.

8. Several grammatical and typographical errors affect readability, e.g., "life cycled assessment" (abstract keywords), "were performed" (abstract), "overs 18 impact categories" (Section 1), and "heirachiest perspective" (Section 4). A thorough language revision is strongly recommended.

Reviewer #3: 1. The abstract could benefit from specifying model performed best in terms of predictive accuracy. This would help clarify the most effective approach for the task.

2. The introduction can be enhanced by providing more context about the limitations or challenges of previous studies. This would help better justify the use of ML models in this study.

3. The literature review section could be expanded to cover more recent developments in machine learning applications to concrete properties.

4. Consider improving the labeling of figures and tables, especially the font size, to make them clearer and more readable.

5. A more detailed discussion of the study’s limitations and a comparison with previous research would strengthen the manuscript by providing a more balanced view.

6. Consider organizing key findings into bullet points in the conclusion to make them stand out more clearly for the reader.

7. Include study limitation and future direction after the conclusion.

8. Reviewing the grammar and sentence structure would help improve the clarity and flow of the manuscript, making it easier to follow.

9. Ensure all figures and tables are appropriately formatted and referenced in the text for consistency and clarity.

Reviewer #4: Please find my detailed comments in the attached Word document.

Dear Authors,

Detailed comments for each section of the manuscript are presented below.

Abstract section:

#1

The abstract explicitly states that Digital Image Correlation (DIC) was used to characterize crack patterns and mechanisms. However, it omits any mention of the Scanning Electron Microscopy (SEM) analysis conducted and discussed extensively in the Results section to evaluate fiber-matrix bonding and microstructural behavior. Furthermore, the abstract mentions comparing the environmental impacts of cement and RCP, but fails to mention that the Life Cycle Assessment (LCA) also extensively compared traditional industrial steel fibers with waste tire steel fibers (WTSF) and the overall mixture impacts.

#2

The abstract states that "the highest flexural strength reached 44.5 MPa with 2% WTSF and 43.5 MPa with 2% polyethylene fiber". However, it does not state the flexural strength of the control or baseline conventional ECC mixture. Without providing the baseline strength, stating the peak strength of the recycled mixtures lacks context; a reader cannot determine whether the addition of RCP and WTSF improved, maintained, or degraded the mechanical performance relative to standard ECC.

#3

The abstract repeats the claim from the conclusion that there was "up to a 16% reduction in climate change potential (GWP20) and 19% reduction in fossil resource use". As identified in the review of Section 4, these percentages are derived from flawed data tables where the baseline ECC carbon footprint was erroneously copied directly from the steel fiber carbon footprint (1350 g CO₂-Eq). Because the foundational LCA data in Table 9 is incorrect, these specific percentage reductions highlighted in the abstract are invalid and must be recalculated once the mass balances and functional units in Section 4 are corrected.

Section 1:

#1

The authors explicitly state that this manuscript is the second part of a previously published study, extending it through additional mechanical and LCA investigations. This raises a significant concern regarding incremental publication, as it is unclear why these findings were not integrated into the original paper. The introduction needs stronger justification, outlining the distinct scientific gaps this continuation addresses to warrant a standalone publication.

#2

While the introduction outlines an intent to conduct a detailed LCA covering 18 impact categories at the manufacturing stage, it lacks fundamental definitional rigor. A robust LCA introduction for sustainable concrete should establish the intended functional unit and clarify the specific system boundaries. Without defining the underlying life cycle inventory database or geographic context early on, the justification for the environmental assessment remains incomplete.

#3

The authors claim they will investigate cracking behavior to clarify how recycled concrete powder influences "matrix densification and fiber-matrix interaction". However, macroscopic crack-pattern analysis alone is insufficient to definitively prove internal matrix densification or microscale bonding interactions. The introduction raises a major concern by failing to mention advanced microstructural characterization techniques, such as SEM, which are essential for validating these specific internal mechanisms.

Section 2:

#1

There is a major contradiction regarding the use of supplementary cementitious materials (SCMs). In the "Materials" subsection, the text explicitly states that fly ash (FA) is used as an SCM along with cement. However, Table 2 (Mix design) omits fly ash, instead listing "Slag" (187.5 kg/m³) alongside Silica Fume (SF). Furthermore, the text references SF, but the particle-size distribution shown in Figure 1 lists only cement, RCP, SF, and FA, creating further confusion about what was actually used in the mix.

#2

In Table 1, the density of Waste Tire Steel Fiber (WTSF) is given as 7.85 g/cm³, while the density of PE fiber is 0.95 g/cm³. The text states that both fibers are used as replacements at 0.5%, 1.0%, 1.5%, and 2.0% "by weight". However, in Engineered Cementitious Composites (ECC) design, fiber dosages are specified by volume fraction (Vf) rather than weight, because the large density difference between steel and polymer fibers means that equal weight fractions yield drastically different volume fractions. This fundamentally alters the fiber spacing and crack-bridging mechanics.

#3

The naming convention in Table 2 does not match the materials used. The nomenclature uses "SF" (e.g., RCP-5-ECC-SF0.5) to denote what is presumably the steel fiber (WTSF) variation, but "SF" was previously defined in the text as Silica Fume. This makes it incredibly difficult to determine whether the samples contain varying amounts of silica fume or steel fiber. Additionally, Table 2 indicates a water content of 477 kg/m³ for a cementitious content of 1500 kg/m³, resulting in a water-to-binder ratio of 0.318; however, it does not account for the superplasticizer mentioned in the text.

#4

There are several formatting issues with the figures and tables:

Figure 1 is referenced as having parts (a), (b), and (c), but the layout and captions are disjointed, and the XRD patterns lack proper crystallographic indexing for the peaks.

Figure 2 is referenced in the text as showing the materials, but the actual caption for Figure 2 states "Experimental setup of RCP-based sustainable ECC," implying it shows the curing process rather than raw materials.

Section 3

#1

The discussion of the flexural strength results claims that adding fibers "compensates for the strength reduction caused by increased RCP content". However, the data presented in the text contradicts this. The maximum flexural strength at 5% RCP is 44.5 MPa, which drops to 42 MPa at 10% RCP, and further down to 40.5 MPa at 15% RCP, all at the same 2% fiber dosage. The text simply lists the numbers without explaining the physical or chemical mechanisms underlying the degradation of strength as RCP increases.

#2

The authors claim that as fiber content increases, "the strain becomes more distributed across the sample, indicating enhanced crack bridging". However, the text immediately contradicts itself by stating that higher fiber contents "result in more localized strain around the mid-span". In ECC research, the hallmark of strain hardening is multiple microcracking, which should manifest in DIC as distributed strain fields rather than localized single cracks. If the strain is localized at the mid-span, it implies a typical single-crack fracture rather than the desired ductile ECC behavior.

#3

The SEM analysis attempts to draw macroscopic conclusions from microscopic observations. The text claims Figure 7(a) shows "brittle behavior due to the partial cement replacement by RCP". SEM cannot definitively prove macroscopic brittle behavior based solely on microcrack observations. Furthermore, the claim that the interaction between the matrix and fibers shows a "strong fiber-matrix interface with good adhesion" in Figure 7(b) lacks quantitative support, such as single-fiber pullout test results, which are standard for verifying interfacial bond strength in ECC.

#4

There are errors in the figure captions and labeling:

In Figure 4, the caption lists parts (a), (b), (d), (d), (e), and (f), skipping (c) and repeating (d).

Figure 5 is missing its caption title (it starts with ": Flexural strength results...") and has the exact same part labeling error as Figure 4 ((a), (b), (d), (d), (e), (f)).[1]

In the DIC section, the text refers to sustainable "UHS-ECC", but this acronym is never defined in this section or the previous ones (it was just "sustainable ECC" before).

Section 4:

#1

The most critical concern in the LCA section is the absolute absence of a defined functional unit (FU). Without an FU (e.g., 1〖" m" 〗^3 of concrete with a specific compressive strength, or 1" kg" of material), comparing conventional ECC with sustainable ECC is fundamentally invalid. Table 9 presents environmental impacts for "Conventional UHS-ECC" and various "Sustainable UHS-ECC" mixes, but it does not state the volume or mass that these impacts represent. Furthermore, the system boundaries for the specific unit processes in Tables 3, 4, 6, and 7 are not explicitly defined with respect to the transportation distances of raw and recycled materials, which are often the largest contributors to the environmental impact of recycled aggregates and powders.

#2

The mass balances presented in the inventory tables are highly questionable and physically impossible in some cases:

In Table 3 (Cement production), the inputs for 1" kg" of cement sums up to roughly 1031" g" (excluding electricity). While slight variations due to moisture or chemical transformations happen, 955" g" of clinker plus 45" g" of gypsum leaves only 0" g" for other SCMs, which do not reflect modern blended Portland cements.

In Table 6 (Steel fiber production), the text states 42.8" g" of unalloyed steel is prepared. However, in the "Wire Drawing" step, the input becomes 1000" g" of steel, and the output is 1000" g" of drawn wire. You cannot draw 1000" g" of wire from an initial raw material preparation of 42.8" g" .

In Table 7 (Waste rubber tire extraction), shredding 1000" g" of waste tires yields exactly 500" g" of shredded rubber and steel wire. What happened to the other 500" g" ? The mass balance is completely broken.

#3

There are contradictory numbers in the impact results:

Table 8 compares "Steel Fiber" to "Waste Tire Steel Fiber" (WTSF). The Climate Change (GWP20) for traditional steel fiber is listed as 1350〖" g CO" 〗_2 "-Eq" . However, in Table 9, the GWP20 for the entire "Conventional UHS-ECC (0% RCP)" mixture is also exactly 1350〖" g CO" 〗_2 "-Eq" . It is impossible for an entire ECC mixture (containing cement, sand, silica fume, water, and fibers) to have the exact same carbon footprint as just the steel fibers.

The same exact copy-paste error occurs for almost every single category in Table 9 for the "Conventional UHS-ECC (0% RCP)" column—the numbers are perfectly identical to the "Steel Fiber" column in Table 8 (e.g., FDP is 236, FETP100 is 27.4, PMFP is 6220).

#4

The text claims that in Table 9, the PMFP decreased from 220 to 200, but Table 9 shows the PMFP actually decreasing from 6220 to 5859.23. The text states "TAP20 showed a major reduction from 5110 to 309" for the sustainable mixture, but Table 9 shows it decreasing from 5110 to 4788.87. The textual discussion does not align with the data presented in the tables.

Section 5

#1

The conclusion states that substituting cement with RCP and using WTSF reduces climate change potential by "16% reduction" and fossil resource use by "19% reduction". However, these percentages directly contradict the claims made in Section 4. In Section 4, the text states that RCP achieves a "99% reduction in greenhouse gas emissions" compared to cement, and FDP is "reduced by over 97%". Furthermore, the reduction from conventional ECC to sustainable ECC (15% RCP) in Table 9 for GWP20 was from 1350 to 1135.38 (a roughly 15.9% reduction), but, as noted previously, the baseline value of 1350 was erroneously copied from the steel fiber alone. The conclusions are summarizing mathematically flawed data.

#2

The final paragraph of the manuscript suggests that future studies should use "machine learning algorithms... trained and implemented in a way that enables deployment through a graphical user interface (GUI)." However, there is absolutely no mention of machine learning, predictive modeling, or algorithm development in the Introduction, Methodology, Results, or LCA sections. Consequently, proposing this as a primary future research direction in an experimental materials and LCA paper without any prior context appears disconnected and inappropriate for this specific manuscript, even though it is a highly valuable suggestion for the field.

To resolve this discrepancy and properly contextualize the recommendation, the authors are advised to include a brief review of machine learning applications in the Introduction section. Specifically, highlighting key features of these methods, such as the distinction between black-box and white-box models and their applications in civil engineering, would provide the necessary theoretical background to justify their inclusion in the manuscript for future studies. The following articles, which discuss white-box and black-box modeling approaches, could serve as useful references for this addition: https://doi.org/10.1016/j.engappai.2026.114277; https://doi.org/10.1007/s11709-025-1237-9; https://doi.org/10.1007/s10661-023-11462-9 and similar articles.

#3

The first bullet point contains confusing syntax: "The highest flexural strength, SF and PE 2% achieved the highest value (44.5 MPa) (43.5 MPa), respectively". This sentence is poorly structured and makes it difficult to read as a definitive concluding statement.

Reviewer #5: After reviewing the manuscript, I consider the topic addressed to be relevant and current, and the results presented to be consistent with the objectives proposed by the study.

However, I suggest that the authors provide some clarifications regarding the sources of the data presented in tables 3 – 4 – 5 – 6. Is it from a database, software, or papers? It was also unclear whether or not statistical treatment of the data obtained was performed.

Therefore, I recommend publishing the article after a review addressing the points mentioned.

Reviewer #6: This manuscript investigates the incorporation of Recycled Concrete Powder (RCP) and Waste Tire Steel Fibers (WTSF) into Engineered Cementitious Composites (ECC). The use of DIC and SEM to visualize crack propagation is of significant value, and the flexural strength results are also impressive demonstrating the recycled materials' performance. However, there are some concerns on fresh-state properties, LCA datas and proof readings.

1. The manuscript lacks fresh-state property data. Fiber inclusion, particularly WTSF, often reduces workability; thus, slump values are critical for practical application and must be reported for all mixtures.

2. There is a major discrepancy in the LCA claims. The abstract claims a "16% reduction in climate change potential (GWP20)" from substituting cement with RCP. However, Table 5 shows a 99% reduction (943 g CO2-Eq for cement vs. 9.41 g CO2-Eq for RCP). The authors must reconcile this.

3. The LCA results presented in Table 9 are not derived from the mix designs reported in this study (Table 2). Instead, they appear identical to the first paper, which utilized separate mix designs. Using LCA data from a different experimental matrix invalidates the current environmental assessment.

4. While flexural and tensile behaviors are evaluated, ECC is fundamentally valued for its durability. The absence of long-term durability data significantly weakens the claim that these recycled materials do not compromise structural performance.

5. The conclusion section must be expanded to explicitly state the limitations and gaps in the current study, acknowledging the practical challenges of these recycled materials.

6. Some formatting and typographical errors need correction. Notably, Figure 4 is labeled "(d)" twice, and the equation numbering starts from [15]. A thorough proof reading is essential.

7. The manuscript states 75 samples were cast but does not specify the number of replicates per mixture. Flexural strength results in Figures 4 and 5 are presented without error bars, standard deviations, or any indication of replication.

**********

what does this mean?). If published, this will include your full peer review and any attached files.

If you choose “no”, your identity will remain anonymous but your review may still be made public.

**Do you want your identity to be public for this peer review?**  For information about this choice, including consent withdrawal, please see our Privacy Policy

Reviewer #1: No

Reviewer #2: No

Reviewer #3: No

Reviewer #4: No

Reviewer #5: No

Reviewer #6: No

**********

[NOTE: If reviewer comments were submitted as an attachment file, they will be attached to this email and accessible via the submission site. Please log into your account, locate the manuscript record, and check for the action link "View Attachments". If this link does not appear, there are no attachment files.]

To ensure your figures meet our technical requirements, please review our figure guidelines: https://journals.plos.org/plosone/s/figures

You may also use PLOS’s free figure tool, NAAS, to help you prepare publication quality figures: https://journals.plos.org/plosone/s/figures#loc-tools-for-figure-preparation.

NAAS will assess whether your figures meet our technical requirements by comparing each figure against our figure specifications.

Attachment Submitted filename: Final commnet.docx

# Author comment: Author response to Decision Letter 1

**DOI:** 10.1371/journal.pone.0355961.r002

5 Jun 2026

RESPONSE TO THE FIRST REVIEWER COMMENTS

Manuscript Number: PONE-D-26-12305

Title: Sustainable Engineered Cementitious Composites Incorporating Recycled Materials: Experimental validation and Life Cycle Assessment

Thank you for taking the time to review this manuscript. Considering your reasonable and constructive comments, we have carefully reviewed the entire manuscript to improve the quality and presentation of the manuscript. Please find the detailed responses below and highlighted revisions/corrections in the resubmitted file.

Comment 1: The abstract succinctly summarizes the key findings, emphasizing the influence of ECC. This clarity is appreciated, but further elaboration on the significance of these findings would enhance the abstract's impact.

Response 1: The authors sincerely thank the reviewer for this positive and constructive comment. In response, the abstract has been revised to further emphasize the broader significance and practical implications of the findings. Additional statements were incorporated to highlight the potential of recycled concrete powder (RCP) and waste tire steel fiber (WTSF) in reducing the environmental footprint of ECC while maintaining desirable mechanical performance.

Comment 2: The selection of keywords appears inadequate, as they fail to comprehensively encompass all facets of the research and accurately communicate the underlying concepts. A revision is recommended to ensure a more precise representation of the study’s scope and content.

Response 2: The authors sincerely thank the reviewer for this valuable suggestion. In response, the keywords section has been revised to better reflect the full scope, methodology, and sustainability aspects of the study. Additional keywords have been incorporated to improve the visibility and representation of the manuscript’s core contributions.

Comment 3: The introduction needs a clearer structure and flow. The information needs to be more -cohesive, making it easier for the reader to follow the logical progression of ideas.

Response 3: The Introduction section has been carefully revised to improve its overall structure, coherence, and logical progression. The discussion has been reorganized to provide a clearer transition from the environmental challenges associated with construction waste and cement production to the development of sustainable ECC. Furthermore, the research gap, study motivation, and novelty of the present work have been clarified to improve readability and strengthen the continuity of ideas throughout the Introduction section.

Comment 4: The introduction does not provide a clear conclusion or summary of the information presented. It is important to provide a concise summary of the key points discussed in the introduction.

Response 4: The Introduction section has been revised to provide a clearer summary of the main research context, identified knowledge gaps, and the primary objectives of the study. Additional statements were incorporated to better highlight the novelty and significance.

Comment 5: The introduction section needs to expand. The literature review seems very limited.

Response 5: The authors sincerely thank the reviewer for this important observation. In response, the Introduction and literature review sections have been substantially expanded by incorporating additional recent and relevant studies related to engineered cementitious composites.

Comment 6: The authors should provide a more explicit delineation of the inherent novelty in their research. It is imperative that they articulate the innovative aspects of their study and specify any prerequisites essential for its execution. This comparative analysis would enhance the clarity and depth of understanding regarding the distinct contributions of the current research in relation to these seminal works in the field. I suggest to add the section of research significance.

Response 6: The authors sincerely thank the reviewer for this insightful and valuable suggestion. In response, a dedicated “Research Significance and Novelty” subsection has been incorporated into the Introduction section to more explicitly highlight the originality and scientific contributions of the present study. The revised section clarifies the distinct aspects of the work in comparison with previous studies, particularly regarding the combined integration of recycled concrete powder (RCP), waste tire steel fiber (WTSF), Digital Image Correlation (DIC)-based crack analysis, SEM investigation, and cradle-to-gate Life Cycle Assessment (LCA) within a unified sustainable ECC framework. Furthermore, the practical and scientific significance of the proposed approach has been elaborated to better demonstrate its contribution toward sustainable and low-carbon construction materials.

Comment 7: Highlighting the novelty and significance of research findings within the introduction would further engage readers.

Response 7: The Introduction section has been revised to more clearly emphasize the novelty, significance, and scientific contribution of the present study. Additionally, a dedicated subsection entitled “Research Significance and Novelty” has been incorporated to explicitly highlight the innovative. The revised discussion now better clarifies the distinct contribution of the present work in comparison with previous studies.

Comment 8: The experimental phase of study was not presented suitable. It should improve.

Response 8: The experimental methodology section has been carefully revised and improved to provide a clearer and more systematic presentation of the experimental program. Additional details regarding material selection, specimen preparation, mix proportions, casting procedure, curing conditions, Digital Image Correlation (DIC) setup, and flexural testing methodology have been incorporated to improve clarity, reproducibility, and technical understanding. Furthermore, the experimental workflow and discussion of testing procedures were refined to ensure better continuity and readability throughout the manuscript.

Comment 9: The regulations and standards of the tests was not presented. All of the regulations for each experiment should provide.

Response 9: Relevant testing standards and experimental regulations used throughout the study have been explicitly incorporated into the revised manuscript. Additional details regarding specimen preparation, flexural testing procedures, curing conditions, and Life Cycle Assessment framework standards have been included to improve methodological clarity, reproducibility, and compliance with established international testing protocols.

Comment 10: More comparative analysis with experimental and other studies in the technical literature should be done.

Response 10: The authors sincerely thank the reviewer for this valuable suggestion. In response, additional comparative discussion with previously published experimental studies has been incorporated throughout the Results and Discussion section. The revised manuscript now includes clearer comparisons regarding flexural strength development, crack-bridging behavior, strain-hardening response, recycled concrete powder (RCP) performance, and waste tire steel fiber (WTSF) effectiveness in relation to findings reported in the existing technical literature. These additions strengthen the interpretation of the experimental results and better position the present study within current ECC research developments.

Comment 11: The conclusion section could benefit from more contextual information about the significance of the research findings. It is helpful to know how the research findings could be applied in real-world situations or how they contribute to existing knowledge in the field.

Response 11: The Conclusions section has been revised to further emphasize the practical implications, scientific contributions, and sustainability significance of the research findings. Additional discussion has been incorporated to clarify how the proposed recycled-material-based ECC can contribute to sustainable and low-carbon construction practices while also advancing the understanding of crack propagation behavior, fiber–matrix interaction, and environmental performance in ECC systems.

Comment 12: Lots of references are outdated. Please expand them. If a suitable position is found, authors can cite references below. [1] Influence of repeated heating–cooling cycles and exposure duration on mechanical, electrical, and durability properties of geopolymer concrete. [2] Residual axial performance of PET/rubber-modified concrete confined with CFRP strips after thermal exposure: Experimental and theoretical analysis. [3] Thermo-mechanical behavior of high-strength concrete with nylon granule aggregates: Experimental evaluation and predictive analysis. [4] Effect of specimen size on compressive capacity and damage mechanisms of PET-containing self-consolidating concrete after high-temperature exposure. [5] Improvement of Recycled Concrete Aggregate Properties by Polyvinyl Alcohol. [6] Evaluation of the Static Behavior of WPC-GFRP Sandwich Panels: An Experimental, Theoretical, and Numerical Study. [6] Improvement of Recycled Concrete Aggregate Properties by Polyvinyl Alcohol

Response 12: Thank you for the valuable comment. The reference list has been carefully revised and expanded by incorporating recent and relevant studies, including the reviewer-suggested articles where suitable. In addition, outdated references were supplemented or replaced with more recent literature related to recycled materials, sustainable cementitious composites, durability performance, and predictive analysis. To clearly present these recent studies and their relevance to the present work, a new literature review summary has been added as Table 1 in the Introduction section.

Comment 13: No in-depth conclusion was found.

Response 13: The authors sincerely thank the reviewer for this important observation. In response, the Conclusions section has been substantially revised and expanded to provide a more in-depth interpretation of the research findings. Additional discussion has been incorporated regarding the mechanical performance enhancement mechanisms, crack propagation behavior, fiber–matrix interaction, and environmental significance of incorporating RCP and WTSF in ECC.

Comment 14: Improve the quality of figures and charts. The quality if the presented figures are not suitable for publication.

Response 14: The quality of all figures and charts has been improved. Low-resolution figures were replaced with high-resolution versions, and the charts were revised to enhance clarity, readability, axis labeling

Comment 15: All the commercial names should be deleted.

Response 15: The authors sincerely thank the reviewer for this valuable observation. In response, commercial and trade names have been carefully reviewed throughout the manuscript and replaced with generic scientific or material descriptions wherever applicable. The revised manuscript now follows a more standardized academic presentation while retaining only essential software and database names required for methodological clarity and reproducibility.

Comment 16: Check the article grammatically.

Response 16: Thank you for the comment. The manuscript has been carefully checked and revised for grammar, sentence structure, clarity, and overall language quality.

Comment 17: All the citations in the manuscript need to be double-checked to ensure their consistency with the reference list.

Response 17: All in-text citations have been carefully checked against the reference list, and necessary corrections have been made to ensure consistency, completeness, and proper citation order throughout the manuscript.

RESPONSE TO THE SECOND REVIEWER COMMENTS

Manuscript Number: PONE-D-26-12305

Title: Sustainable Engineered Cementitious Composites Incorporating Recycled Materials: Experimental validation and Life Cycle Assessment

Thank you for taking the time to review this manuscript. Considering your reasonable and constructive comments, we have carefully reviewed the entire manuscript to improve the quality and presentation of the manuscript. Please find the detailed responses below and highlighted revisions/corrections in the resubmitted file.

Comment 1: The authors claim that the present study is the "second part" of a previously published work [22], but the novelty and specific contribution of this part are not clearly distinguished from the first. Please clearly articulate what new insights—particularly from DIC and LCA—are uniquely provided here beyond the earlier publication.

Response 1: The authors sincerely thank the reviewer for this insightful and important observation. In response, the Introduction section has been revised to more clearly distinguish the novelty and scientific contribution of the present study from the previously published first part of the research. Specifically, additional clarification has been incorporated in the final paragraph of the Introduction section and within the newly added “Research significance” subsection. The revised manuscript now explicitly states that the previous study primarily focused on the preliminary development and environmental feasibility of sustainable ECC mixtures, whereas the current work provides a more comprehensive investigation through Digital Image Correlation (DIC)-based crack propagation analysis, SEM-based fiber–matrix interaction assessment, detailed flexural characterization, and cradle-to-gate Life Cycle Assessment (LCA). Furthermore, the present study uniquely establishes the relationship between crack propagation behavior, strain localization, crack-bridging mechanisms, and environmental impact reduction within a unified sustainable ECC framework, which was not addressed in the previous publication.

Section 1.1. Research significance (Line (140 to 150)

Comment 2: The abstract and methodology state a "cradle-to-gate" LCA, but the interpretation in Section 4 focuses only on material substitution impacts. The system boundary is not clearly drawn for the composite (ECC) level. Please explicitly define the functional unit and system boundary for the LCA of sustainable ECC versus conventional ECC to avoid ambiguity.

Response 2: The authors sincerely thank the reviewer for this valuable and technically important observation. In response, additional clarification regarding the functional unit and system boundary of the Life Cycle Assessment (LCA) has been incorporated into the revised manuscript within the “Life Cycle Assessment (LCA)” subsection. The revised text now explicitly defines the functional unit as 1 m³ of ECC mixture and clarifies that the cradle-to-gate system boundary includes raw material extraction, material processing, transportation, recycling operations, and manufacturing-stage activities associated with both conventional ECC and sustainable ECC mixtures. Furthermore, the revised discussion now more clearly distinguishes material-level environmental impacts from composite-level ECC environmental performance to avoid ambiguity in interpretation.

Section 4. Life cycled assessment (LCA) (Line (330 to 339)

Comment 3: Equation (15) uses "GWD" (likely a typo, should be GWP). Equation (20) is referenced but not shown clearly in the main text. Please correct the typo and ensure all equations are properly numbered and defined.

Response 3: The authors sincerely thank the reviewer for this careful observation. In response, the typographical inconsistency in Equation (15) has been corrected by replacing “GWD” with the correct term “GWP” (Global Warming Potential). In addition, all equations throughout the manuscript were carefully reviewed to ensure proper numbering, formatting, and definition of variables. Equation (20) and its associated parameters were also revised and clarified in the main text to improve readability and technical consistency.

Section : 4.1. LCA of cement vs RCP (Table 5, 8, 9) new (Table 6, 9, 10)

Comment 4: The LCA results in Tables 5, 8, and 9 show very high precision (e.g., 1293.5 g CO₂-eq). It is unclear whether this reflects actual model precision or false precision from the ecoinvent database. Please report uncertainty ranges or sensitivity analyses, especially for key impact categories like GWP

Attachment Submitted filename: Response to Eidtor and Reviewers comments.pdf

# Peer review: Decision Letter 1

**DOI:** 10.1371/journal.pone.0355961.r003

**Authors:** Badrinarayan Rath

15 Jul 2026

Dear Dr. Ahmad,

Thank you for submitting your manuscript to PLOS ONE. After careful consideration, we feel that it has merit but does not fully meet PLOS ONE’s publication criteria as it currently stands. Therefore, we invite you to submit a revised version of the manuscript that addresses the points raised during the review process.

Please submit your revised manuscript by Aug 29 2026 11:59PM. If you will need more time than this to complete your revisions, please reply to this message or contact the journal office at plosone@plos.org. When you're ready to submit your revision, log on to https://www.editorialmanager.com/pone/ and select the 'Submissions Needing Revision' folder to locate your manuscript file.

- A letter that responds to each point raised by the academic editor and reviewer(s). You should upload this letter as a separate file labeled 'Response to Reviewers'.
- A marked-up copy of your manuscript that highlights changes made to the original version. You should upload this as a separate file labeled 'Revised Manuscript with Track Changes'.
- An unmarked version of your revised paper without tracked changes. You should upload this as a separate file labeled 'Manuscript'.

If you would like to make changes to your financial disclosure, please include your updated statement in your cover letter. Guidelines for resubmitting your figure files are available below the reviewer comments at the end of this letter.

If applicable, we recommend that you deposit your laboratory protocols in protocols.io to enhance the reproducibility of your results. Protocols.io assigns your protocol its own identifier (DOI) so that it can be cited independently in the future. For instructions see: https://journals.plos.org/plosone/s/submission-guidelines#loc-laboratory-protocols. Additionally, PLOS ONE offers an option for publishing peer-reviewed Lab Protocol articles, which describe protocols hosted on protocols.io. Read more information on sharing protocols at https://plos.org/protocols?utm_medium=editorial-email&utm_source=authorletters&utm_campaign=protocols.

As the corresponding author, your ORCID iD is verified in the submission system and will appear in the published article. PLOS supports the use of ORCID, and we encourage all coauthors to register for an ORCID iD and use it as well. Please encourage your coauthors to verify their ORCID iD within the submission system before final acceptance, as unverified ORCID iDs will not appear in the published article. *Only*  the individual author can complete the verification step; PLOS staff *cannot*  verify ORCID iDs on behalf of authors.

We look forward to receiving your revised manuscript.

Kind regards,

Badrinarayan Rath, PhD

Academic Editor

PLOS One

Journal Requirements:

If the reviewer comments include a recommendation to cite specific previously published works, please review and evaluate these publications to determine whether they are relevant and should be cited. There is no requirement to cite these works unless the editor has indicated otherwise.

[Note: HTML markup is below. Please do not edit.]

Reviewers' comments:

Reviewer's Responses to Questions

**Comments to the Author**

Reviewer #3: (No Response)

Reviewer #4: (No Response)

Reviewer #5: All comments have been addressed

Reviewer #6: All comments have been addressed

**********

2. Is the manuscript technically sound, and do the data support the conclusions?

Reviewer #3: (No Response)

Reviewer #4: Partly

Reviewer #5: Yes

Reviewer #6: Yes

**********

3. Has the statistical analysis been performed appropriately and rigorously?

Reviewer #3: (No Response)

Reviewer #4: No

Reviewer #5: Yes

Reviewer #6: Yes

**********

4. Have the authors made all data underlying the findings in their manuscript fully available?

The PLOS Data policy

Reviewer #3: (No Response)

Reviewer #4: No

Reviewer #5: Yes

Reviewer #6: Yes

**********

5. Is the manuscript presented in an intelligible fashion and written in standard English?

Reviewer #3: (No Response)

Reviewer #4: Yes

Reviewer #5: Yes

Reviewer #6: Yes

**********

Reviewer #3: Interesting research. The author has revised the manuscript and it has met the requirements for publication.

Reviewer #4: Dear Authors,

Thank you for your considerable efforts in revising the manuscript and addressing the previous reviewers' comments. The revised version has improved substantially. However, several important issues still require clarification or further revision to strengthen the manuscript's scientific quality and clarity. Please consider the following comments.

1. The Introduction provides a useful overview of previous studies but remains largely descriptive. Although Table 1 summarizes recent research, the manuscript does not clearly identify the remaining research gap or explain how the present study advances beyond previous work. The authors should include a concise critical discussion that highlights the limitations of existing studies and explicitly state the scientific gap this study addresses.

2. Table 1 should be expanded to provide a more comprehensive comparison of recent studies on sustainable ECC, particularly those involving recycled concrete powder, recycled fibers, DIC, SEM, and LCA. Including consistent comparison criteria (e.g., recycled materials, characterization methods, and environmental assessment) would better demonstrate the novelty of the present study. Since DIC, SEM, and LCA have already been widely applied in ECC research, the novelty should be presented as the integration of these approaches rather than the individual techniques themselves.

3. The research objectives are scattered throughout the Introduction. They should be summarized into 3–5 clear, specific objectives to improve the manuscript's readability and focus.

4. The Introduction would benefit from a clearer scientific rationale for using RCP. Important factors influencing RCP performance (e.g., particle size, fineness, chemical composition, and residual hydration products) are not discussed. In addition, the expected mechanisms by which RCP and WTSF improve ECC performance should be briefly explained, preferably through a clear scientific hypothesis.

5. The Introduction should briefly justify the selection of a cradle-to-gate LCA and explain why this system boundary was chosen instead of other common approaches (e.g., cradle-to-grave or cradle-to-cradle). A short explanation of the selected impact categories would also strengthen the environmental motivation.

6. The "Research Significance" section overlaps considerably with the Introduction. Instead of repeating the study objectives, it should focus on the main scientific contribution, engineering relevance, and practical significance of the work.

7. The overall experimental design is not described clearly. The methodology and Figure 1 primarily present the experimental procedures but do not adequately explain the experimental matrix, the number of mixtures and specimens, the factorial design, or how the experimental program and LCA are integrated. Figure 1 should be expanded to better illustrate the workflow, experimental design, and the relationship between the mechanical, DIC, SEM, and LCA analyses.

8. The fiber replacement strategy requires clarification. The manuscript should clearly specify whether the replacement levels are based on mass or volume, whether the replacement is partial or complete, and how the PE fiber content varies with WTSF. In addition, the rationale for selecting the RCP and fiber replacement levels under investigation should be briefly explained.

9. The descriptions of the DIC and SEM methodologies are insufficient for reproducibility. Essential experimental details, including equipment specifications, specimen preparation, calibration, testing parameters, and image acquisition/analysis procedures, should be provided.

10. The methodology should introduce the LCA functional unit at the beginning of the section and more clearly explain how the mechanical tests, DIC, SEM observations, and LCA are integrated to support the overall interpretation of the study.

11. The methodology does not describe the statistical analysis used to evaluate the experimental results. Considering the variability of fiber-reinforced composites, the statistical procedures used to assess repeatability and significance should be reported.

12. The mechanical testing procedure should be described in greater detail, including specimen dimensions, loading configuration, loading rate, span length, curing conditions, testing age, and the applicable testing standard.

13. Figure 1 could be further improved by including key validation steps, such as repeatability assessment, statistical validation, uncertainty evaluation, and verification of the LCA results, to better reflect the overall methodological framework.

14. The mix design requires further justification. The rationale for selecting the RCP and fiber replacement levels investigated is insufficiently explained, and the experimental design should be better justified. In addition, fiber content is defined by mass rather than volume, despite the substantial density difference between WTSF and PE fibers. The authors should explain this choice and discuss its implications for fiber distribution and crack-bridging behavior.

15. The characterization of the recycled materials is incomplete. More comprehensive information should be provided for both RCP and WTSF, including the key physical, chemical, and microstructural properties that may influence ECC performance. In addition, the variability and quality control of the recycled materials should be discussed to improve the study's reproducibility.

16. Table 3 is unnecessarily repetitive because most mixture parameters remain constant. The table could be simplified by presenting the constant components once and highlighting only the variables among the mixtures.

17. The specimen preparation procedure requires further justification. In particular, the accelerated curing regime (85°C for 9 days) and the workability evaluation should be discussed in greater detail, including the reasons for selecting these conditions and their potential influence on ECC performance.

18. The experimental methodology requires additional detail to ensure reproducibility. Important information related to the mechanical testing procedure, DIC configuration, and statistical analysis (e.g., loading conditions, DIC parameters, and statistical validation of the experimental results) should be provided.

19. The specimen nomenclature is not sufficiently clear. A concise table explaining the naming convention would improve the manuscript's readability.

20. The manuscript focuses mainly on flexural behavior. If flexural testing is intended to represent the overall mechanical performance of ECC, the authors should justify this choice or discuss the absence of complementary mechanical tests, such as direct tensile or compressive strength.

21. The discussion is largely descriptive rather than mechanistic. The authors mainly describe the observed trends without sufficiently explaining the underlying mechanisms. In particular, the effects of RCP and WTSF on crack propagation, fiber bridging, post-peak behavior, and failure mechanisms should be interpreted in light of microstructural evidence and established ECC theories.

22. The reported improvements should be supported by appropriate statistical analysis. Although error bars are presented, the manuscript does not report statistical significance, variability, or uncertainty (e.g., standard deviation, ANOVA, confidence intervals, or measurement uncertainty). Consequently, some claims of significant improvement should be moderated or statistically validated.

23. The quantitative DIC analysis is underutilized. Table 4 contains valuable information; however, most of the reported parameters are not adequately interpreted. In addition, the proposed DIC indices should be better justified, validated, and supported with appropriate references if they are not standard metrics.

24. The SEM analysis remains largely qualitative. The conclusions regarding fiber–matrix bonding and microstructural behavior appear stronger than the presented evidence. A more quantitative interpretation (e.g., crack width, ITZ characteristics, or pore structure) would strengthen the discussion and better support the proposed mechanisms.

25. The relationships among the flexural test results, DIC measurements, and SEM observations are not sufficiently established. Demonstrating quantitative correlations between these techniques would significantly strengthen the scientific contribution of the study.

26. The discussion would benefit from a more quantitative comparison with previous ECC studies. Rather than stating that the results are consistent with the literature, the authors should compare the magnitude of the improvements in strength, ductility, crack characteristics, and sustainability with previously published results.

27. The discussion of sustainability remains largely separated from the mechanical results. A more integrated interpretation that highlights the trade-off between environmental benefits and mechanical performance, including the optimal RCP and fiber content, would considerably strengthen the manuscript.

28. The LCA methodology requires greater transparency. The manuscript should explain how the reported uncertainty ranges (±10%) were obtained and clearly describe the allocation approach adopted for recycled materials (e.g., cut-off, mass allocation, economic allocation, or system expansion). These assumptions are essential for evaluating the reliability of the environmental assessment.

29. Rather than discussing all 18 impact categories equally, the manuscript should focus on the most relevant indicators (e.g., GWP, FDP, PMFP, TAP, and WDP) to provide a deeper and more meaningful interpretation.

30. The interpretation of the LCA results should be strengthened. The discussion remains largely descriptive and does not adequately explain why certain impact categories behave differently (e.g., ODP and water depletion). In addition, a quantitative comparison with previously published LCA studies would help validate the reported environmental impacts and place the results in the context of the existing literature.

31. One of the main strengths claimed by the manuscript is the integration of mechanical performance and environmental assessment; however, these two aspects are discussed largely independently. The manuscript would be considerably strengthened by identifying the optimal ECC mixture based on both mechanical performance and environmental impact, for example, through a performance-versus-carbon comparison or a multi-objective comparison.

32. The Conclusions should go beyond summarizing the experimental results by identifying the optimum ECC mixture based on both mechanical and environmental performance. In addition, the practical engineering implications of the proposed sustainable ECC, including its potential applications, implementation, and sustainability benefits, should be discussed more explicitly.

33. The "Future Research Directions" section is rather generic. Instead of listing common recommendations, the authors should identify the specific limitations of the present study and propose more focused directions for future research based on the remaining unresolved scientific questions.

34. The Supplementary Information should be better organized by including a clear structure (table of contents, section headings, table numbering, captions, specimen identifiers, and units), making it fully self-contained and easier to follow.

35. The Data Availability Statement and Supplementary Information should be revised to improve reproducibility. In particular, the software/database versions used for the LCA should be reported, and the statement regarding the availability of "codes" should be revised if no source code is actually provided.

Reviewer #5: (No Response)

Reviewer #6: The authors have addressed the comments i made and they have made the necessary revisions to improve the manuscript. The methodology and discussion are now clearer, the limitations have been acknowledged, and the issues related to specimen replication, statistical reporting, and formatting have been resolved. Overall, I believe the manuscript has definitely improved and is suitable for publication.

**********

what does this mean?). If published, this will include your full peer review and any attached files.

If you choose “no”, your identity will remain anonymous but your review may still be made public.

**Do you want your identity to be public for this peer review?**  For information about this choice, including consent withdrawal, please see our Privacy Policy

Reviewer #3: No

Reviewer #4: No

Reviewer #5: No

Reviewer #6: No

**********

[NOTE: If reviewer comments were submitted as an attachment file, they will be attached to this email and accessible via the submission site. Please log into your account, locate the manuscript record, and check for the action link "View Attachments". If this link does not appear, there are no attachment files.]

To ensure your figures meet our technical requirements, please review our figure guidelines: https://journals.plos.org/plosone/s/figures

You may also use PLOS’s free figure tool, NAAS, to help you prepare publication quality figures: https://journals.plos.org/plosone/s/figures#loc-tools-for-figure-preparation.

NAAS will assess whether your figures meet our technical requirements by comparing each figure against our figure specifications.

# Author comment: Author response to Decision Letter 2

**DOI:** 10.1371/journal.pone.0355961.r004

20 Jul 2026

Response to Reviewer #1

We sincerely thank the reviewer for reviewing the revised manuscript and confirming that the previous comments have been adequately addressed. We greatly appreciate the reviewer’s valuable suggestions, which contributed significantly to improving the quality and clarity of the manuscript.

Response to Reviewer #2

We sincerely thank the reviewer for the careful assessment of our revised manuscript and for considering it acceptable for publication. The reviewer’s constructive feedback was highly valuable and enabled us to strengthen the methodology, presentation of the results, and scientific discussion.

Response to Reviewer #3

We sincerely thank the reviewer for the positive assessment of our research and for confirming that the revised manuscript meets the requirements for publication. We greatly appreciate the reviewer’s time, encouraging comments, and valuable contribution to improving the manuscript.

Response to Reviewer #4

Comment 1: The Introduction provides a useful overview of previous studies but remains largely descriptive. Although Table 1 summarizes recent research, the manuscript does not clearly identify the remaining research gap or explain how the present study advances beyond previous work. The authors should include a concise critical discussion that highlights the limitations of existing studies and explicitly state the scientific gap this study addresses.

Response 1: Thank you for this important observation. The final part of the Introduction has been rewritten to move beyond a descriptive literature summary and to identify the remaining scientific gap explicitly. The revised text explains that previous studies generally considered recycled binders, recycled fibers, mechanical behavior, DIC, SEM, or LCA separately, whereas the present study integrates flexural testing, DIC-derived strain behavior, qualitative SEM evidence, and cradle-to-gate LCA in one framework. The novelty is now stated as the integrated mechanical–microstructural–environmental interpretation. These additions are highlighted in yellow in the revised manuscript.

Comment 2: Table 1 should be expanded to provide a more comprehensive comparison of recent studies on sustainable ECC, particularly those involving recycled concrete powder, recycled fibers, DIC, SEM, and LCA. Including consistent comparison criteria (e.g., recycled materials, characterization methods, and environmental assessment) would better demonstrate the novelty of the present study. Since DIC, SEM, and LCA have already been widely applied in ECC research, the novelty should be presented as the integration of these approaches rather than the individual techniques themselves.

Response 2: Table 1 has been expanded with additional entries directly related to recycled concrete-derived ECC, recycled tire steel fibers, waste-based cementitious composites, and LCA. A final row has also been added for the present study, clearly showing that its contribution is the combined use of flexural testing, DIC, SEM, and LCA. The text now avoids presenting DIC, SEM, or LCA individually as novel and instead identifies their integration as the main advance. The existing table format and all reported values were retained.

Comment 3: The research objectives are scattered throughout the Introduction. They should be summarized into 3–5 clear, specific objectives to improve the manuscript's readability and focus.

Response 3: Thank you for this constructive comment. The Introduction has been revised to improve its organization, readability, and focus. The research objectives, which were previously distributed across different parts of the Introduction, are now consolidated and clearly presented in the subsection entitled “Research significance.” The revised subsection explains that the study aims to develop sustainable ECC mixtures incorporating recycled concrete powder as a partial cement replacement and waste tire steel fiber as an alternative reinforcing material, evaluate their flexural performance and crack development using conventional testing and Digital Image Correlation, examine fiber and matrix interaction and crack bridging mechanisms through SEM analysis, and quantify their environmental performance through a cradle to gate life cycle assessment based on a functional unit of 1 m³ of ECC.

It also clarifies that the novelty of the work lies in integrating mechanical performance, full field strain behavior, microstructural evidence, and environmental assessment within a single framework. This revision provides a clearer statement of the study objectives and helps readers understand the scope, depth, and contribution of the research at the end of the Introduction.

Comment 4: The Introduction would benefit from a clearer scientific rationale for using RCP. Important factors influencing RCP performance (e.g., particle size, fineness, chemical composition, and residual hydration products) are not discussed. In addition, the expected mechanisms by which RCP and WTSF improve ECC performance should be briefly explained, preferably through a clear scientific hypothesis.

Response 4: A scientific rationale and working hypothesis have been added to the Introduction. The revised text explains the expected particle-filling and residual siliceous contribution of low-to-moderate RCP contents, the possible porosity and stress-transfer limitations at higher RCP contents, and the crack-bridging and strain-redistribution roles of WTSF and PE fibers. The Materials section also reports the available particle-size and XRD information for RCP. The hypothesis now anticipates a trade-off region in which recycled content can be increased while retaining acceptable flexural performance.

Comment 5: The Introduction should briefly justify the selection of a cradle-to-gate LCA and explain why this system boundary was chosen instead of other common approaches (e.g., cradle-to-grave or cradle-to-cradle). A short explanation of the selected impact categories would also strengthen the environmental motivation.

Response 5: Thank you for this valuable comment. The Introduction has been revised to include a brief justification for adopting a cradle to gate LCA. The revised text explains that this boundary was selected because the principal differences among the investigated ECC mixtures arise during raw material extraction, recycled material processing, transportation, and composite production. Cradle to grave and cradle to cradle approaches were not adopted because they would require additional assumptions regarding service life, maintenance, demolition, recovery, recycling, and disposal, which depend strongly on the final structural application and regional waste management conditions. The environmental motivation has also been strengthened by briefly explaining the selected impact categories, including climate change, fossil resource depletion, toxicity, particulate matter formation, acidification, eutrophication, land occupation, water consumption, and resource depletion. These revisions are now included in the Introduction to clarify the scope, relevance, and environmental depth of the LCA assessment.

Comment 6: The 'Research Significance' section overlaps considerably with the Introduction. Instead of repeating the study objectives, it should focus on the main scientific contribution, engineering relevance, and practical significance of the work.

Response 6: Thank you for this constructive comment. The “Research Significance” section has been revised to remove repetition of the study objectives already presented in the Introduction. The revised section now focuses specifically on the scientific contribution, engineering relevance, and practical significance of the work. Scientifically, the study establishes an integrated framework that connects flexural performance, DIC based strain and crack development, SEM based fiber and matrix interaction, and cradle to gate LCA. From an engineering perspective, this framework helps explain how RCP and WTSF influence matrix quality, crack control, post cracking behavior, and overall structural performance. Practically, the study provides evidence for the use of recycled concrete powder and waste tire steel fiber as viable low carbon alternatives in ECC and supports mixture selection based on both mechanical efficiency and environmental impact. The revised section therefore emphasizes the broader contribution of the research rather than restating its objectives.

Comment 7: The overall experimental design is not described clearly. The methodology and Figure 1 primarily present the experimental procedures but do not adequately explain the experimental matrix, the number of mixtures and specimens, the factorial design, or how the experimental program and LCA are integrated. Figure 1 should be expanded to better illustrate the workflow, experimental design, and the relationship between the mechanical, DIC, SEM, and LCA analyses.

Response 7: Thank you for this important comment. The methodology and Figure 1 have been revised to present the overall experimental design more clearly. The revised methodology now specifies the experimental matrix, including the RCP replacement levels, fiber systems, fiber dosages, number of mixtures, specimen allocation, and replicate testing. It also clarifies that the study follows a comparative selected matrix rather than a full factorial design.

Figure 1 has been expanded with additional details on material preparation, mix design, casting, curing, flexural testing, and the subsequent mechanical, DIC, and SEM analyses. The cradle to gate LCA phase now clearly presents the functional unit, system boundary, inventory sources, impact assessment method, comparative assessment levels, and environmental outputs. The revised figure also demonstrates how the experimental and environmental datasets are combined during the integrated interpretation to relate flexural performance, strain and crack evolution, microstructural evidence, and environmental impacts. These revisions provide a clearer and more complete representation of the research workflow and the connection between the experimental program and LCA.

Comment 8: The fiber replacement strategy requires clarification. The manuscript should clearly specify whether the replacement levels are based on mass or volume, whether the replacement is partial or complete, and how the PE fiber content varies with WTSF. In addition, the rationale for selecting the RCP and fiber replacement levels under investigation should be briefly explained.

Response 8: The fiber strategy has been clarified throughout the methodology and mix-design discussion. Fiber dosages are now explicitly defined as percentages by mass of binder. In the WTSF series, PE fiber is maintained at 2.0% while WTSF varies from 0.5% to 2.0%; in the PE series, WTSF is maintained at 2.0% while PE varies over the same range. The manuscript also clarifies that WTSF is used as recycled steel reinforcement in the experimental mixtures, while the industrial-steel-fiber comparison is part of the LCA. The rationale for the selected RCP and fiber ranges and the implications of the density difference between WTSF and PE fibers are now discussed.

Comment 9: The descriptions of the DIC and SEM methodologies are insufficient for reproducibility. Essential experimental details, including equipment specifications, specimen preparation, calibration, testing parameters, and image acquisition/analysis procedures, should be provided.

Response 9: The DIC and SEM methodology sections have been strengthened using the information available from the study records. The DIC section now reports speckle preparation, fixed-camera/controlled-lighting acquisition, a 3 s image interval, approximately 15 µm/pixel resolution, the purpose of the monitored region, and the interpretation of the reported indices. A separate SEM procedure paragraph now defines the features examined and clarifies that the SEM evidence is qualitative. Instrument-specific settings that could not be reliably reconstructed were not invented; instead, the scope and reproducibility limitation are stated explicitly and the associated claims have been moderated.

Comment 10: The methodology should introduce the LCA functional unit at the beginning of the section and more clearly explain how the mechanical tests, DIC, SEM observations, and LCA are integrated to support the overall interpretation of the study.

Response 10: The LCA functional unit of 1 m³ of ECC is now introduced at the beginning of the LCA methodology. The general methodology also explains how flexural results, DIC descriptors, SEM observations, and LCA outputs are combined to identify performance–environmental trade-offs. This integrated interpretation is reinforced later through a dedicated mechanical–environmental assessment subsection.

Comment 11: The methodology does not describe the statistical analysis used to evaluate the experimental results. Considering the variability of fiber-reinforced composites, the statistical procedures used to assess repeatability and significance should be reported.

Response 11: A new statistical treatment and repeatability paragraph has been added. It states that three replicate beams were tested for each mixture, explains that the error bars represent reported replicate variability, and clarifies that a full inferential statistical analysis was not available in the reported dataset. Therefore, unsupported claims of statistical significance have been removed or moderated. The manuscript now explicitly recommends reporting individual replicates, SD, CV, confidence intervals, ANOVA, and post-hoc tests in future work rather than presenting unverified p-values.

Comment 12: The mechanical testing procedure should be described in greater detail, including specimen dimensions, loading configuration, loading rate, span length, curing conditions, testing age, and the applicable testing standard.

Response 12: The mechanical testing description now reports the total number of specimens, three replicates per mixture, beam dimensions of 40 × 40 × 160 mm, three-point bending, ASTM C348, the common curing/storage sequence, and DIC surface preparation. The revised text also states that the loading configuration, support arrangement, and loading procedure followed the adopted ASTM C348 protocol. No unverified machine or loading values were introduced, and the limitations of the available procedural record are acknowledged.

Comment 13: Figure 1 could be further improved by including key validation steps, such as repeatability assessment, statistical validation, uncertainty evaluation, and verification of the LCA results, to better reflect the overall methodological framework.

Response 13: Repeatability and uncertainty-related elements are now described in the text surrounding Figure 1 and in the statistical and LCA methodology paragraphs. Triplicate beam testing is identified as the repeatability check, while the ±10% LCA ranges are defined as deterministic screening bounds rather than confidence intervals. Because the existing figure image and numerical content were to remain unchanged, Figure 1 was retained, but the missing validation and uncertainty information is now supplied in the adjacent highlighted text and the Study Limitations section.

Comment 14: The mix design requires further justification. The rationale for selecting the RCP and fiber replacement levels investigated is insufficiently explained, and the experimental design should be better justified. In addition, fiber content is defined by mass rather than volume, despite the substantial density difference between WTSF and PE fibers. The authors should explain this choice and discuss its implications for fiber distribution and crack-bridging behavior.

Response 14: The mix-design rationale has been expanded. The RCP range of 5–15% is described as a low-to-moderate replacement range intended to obtain environmental benefit while limiting adverse effects associated with excessive recycled powder. The 0.5–2.0% fiber range is explained as covering lower reinforcement through higher crack-bridging capacity. The manuscript now explicitly states that mass-based dosages do not provide equivalent WTSF and PE volume fractions because of their density differ

Attachment Submitted filename: Response to Reviewers comments.pdf

# Peer review: Decision Letter 2

**DOI:** 10.1371/journal.pone.0355961.r005

**Authors:** Badrinarayan Rath

28 Jul 2026

Sustainable Engineered Cementitious Composites Incorporating Recycled Materials: Experimental validation and Life Cycle Assessment

PONE-D-26-12305R2

Dear Dr. Ahmad,

We’re pleased to inform you that your manuscript has been judged scientifically suitable for publication and will be formally accepted for publication once it meets all outstanding technical requirements.

Within one week, you’ll receive an e-mail detailing the required amendments. When these have been addressed, you’ll receive a formal acceptance letter and your manuscript will be scheduled for publication.

An invoice will be generated when your article is formally accepted. Please note, if your institution has a publishing partnership with PLOS and your article meets the relevant criteria, all or part of your publication costs will be covered. Please make sure your user information is up-to-date by logging into Editorial Manager at Editorial Manager® and clicking the ‘Update My Information' link at the top of the page. For questions related to billing, please contact billing support.

If your institution or institutions have a press office, please notify them about your upcoming paper to help maximize its impact. If they’ll be preparing press materials, please inform our press team as soon as possible -- no later than 48 hours after receiving the formal acceptance. Your manuscript will remain under strict press embargo until 2 pm Eastern Time on the date of publication. For more information, please contact onepress@plos.org.

Kind regards,

Badrinarayan Rath, PhD

Academic Editor

PLOS One

Additional Editor Comments (optional):

Reviewers' comments:

Reviewer's Responses to Questions

**Comments to the Author**

Reviewer #4: (No Response)

**********

2. Is the manuscript technically sound, and do the data support the conclusions?

Reviewer #4: (No Response)

**********

3. Has the statistical analysis been performed appropriately and rigorously?

Reviewer #4: (No Response)

**********

4. Have the authors made all data underlying the findings in their manuscript fully available?

The PLOS Data policy

Reviewer #4: (No Response)

**********

5. Is the manuscript presented in an intelligible fashion and written in standard English?

Reviewer #4: (No Response)

**********

Reviewer #4: Dear Authors,

Thank you for your efforts in improving the manuscript.

I believe that the manuscript has been significantly improved and is now suitable for acceptance.

**********

what does this mean?). If published, this will include your full peer review and any attached files.

If you choose “no”, your identity will remain anonymous but your review may still be made public.

**Do you want your identity to be public for this peer review?**  For information about this choice, including consent withdrawal, please see our Privacy Policy

Reviewer #4: No

**********

# Editor report: Acceptance letter

**DOI:** 10.1371/journal.pone.0355961.r006

**Authors:** Badrinarayan Rath

PONE-D-26-12305R2

PLOS One

Dear Dr. Ahmad,

I'm pleased to inform you that your manuscript has been deemed suitable for publication in PLOS One. Congratulations! Your manuscript is now being handed over to our production team.

At this stage, our production department will prepare your paper for publication. This includes ensuring the following:

* All references, tables, and figures are properly cited

* All relevant supporting information is included in the manuscript submission,

* There are no issues that prevent the paper from being properly typeset

You will receive further instructions from the production team, including instructions on how to review your proof when it is ready. Please keep in mind that we are working through a large volume of accepted articles, so please give us a few days to review your paper and let you know the next and final steps.

Lastly, if your institution or institutions have a press office, please let them know about your upcoming paper now to help maximize its impact. If they'll be preparing press materials, please inform our press team within the next 48 hours. Your manuscript will remain under strict press embargo until 2 pm Eastern Time on the date of publication. For more information, please contact onepress@plos.org.

You will receive an invoice from PLOS for your publication fee after your manuscript has reached the completed accept phase. If you receive an email requesting payment before acceptance or for any other service, this may be a phishing scheme. Learn how to identify phishing emails and protect your accounts at https://explore.plos.org/phishing.

If we can help with anything else, please email us at customercare@plos.org.

Thank you for submitting your work to PLOS One and supporting open access.

Kind regards,

PLOS ONE Editorial Office Staff

on behalf of

Dr. Badrinarayan Rath

Academic Editor

PLOS One
